# Supplementary material for: Local area public sector spending and nutritional anaemia hospital admissions in England: a longitudinal ecological study
Source: BMJ Open. 2022 Sep 28;12(9):e059739. doi: 10.1136/bmjopen-2021-059739 (PMC9528630; doi:10.1136/bmjopen-2021-059739)
Supplement: Supplementary data [file bmjopen-2021-059739supp001.pdf]

Appendix 1: ICD-10 codes for conditions included in this study

**ICD-10 code**	**Diagnosis**
Principal and total nutritional anaemia hospital admissions:	
D50	Iron deficiency anaemia
D51	Vitamin B12 deficiency anaemia
D52	Folate deficiency anaemia
D53	Other nutritional anaemias
Excluding admissions alongside a principal or secondary diagnosis of:	
D50.0	Iron deficiency anaemia secondary to blood loss
C00-C96	Malignancies
F50	Eating disorders
R13	Difficulty swallowing
K91.2	Postoperative malabsorption
R64	Cachexia
E70-90	Metabolic disorders
K50.90, K51.90	GI disease including Crohn’s disease and ulcerative colitis
K63.2	Fistula
Z99.2, N17-19	Kidney disease and dialysis
K70-77	Liver disease
E08-E11, O24.4	Diabetes mellitus (due to underlying condition, drug or chemical induced, type I, type II, gestational)

Appendix 2: Changes in mean total LA spending over time

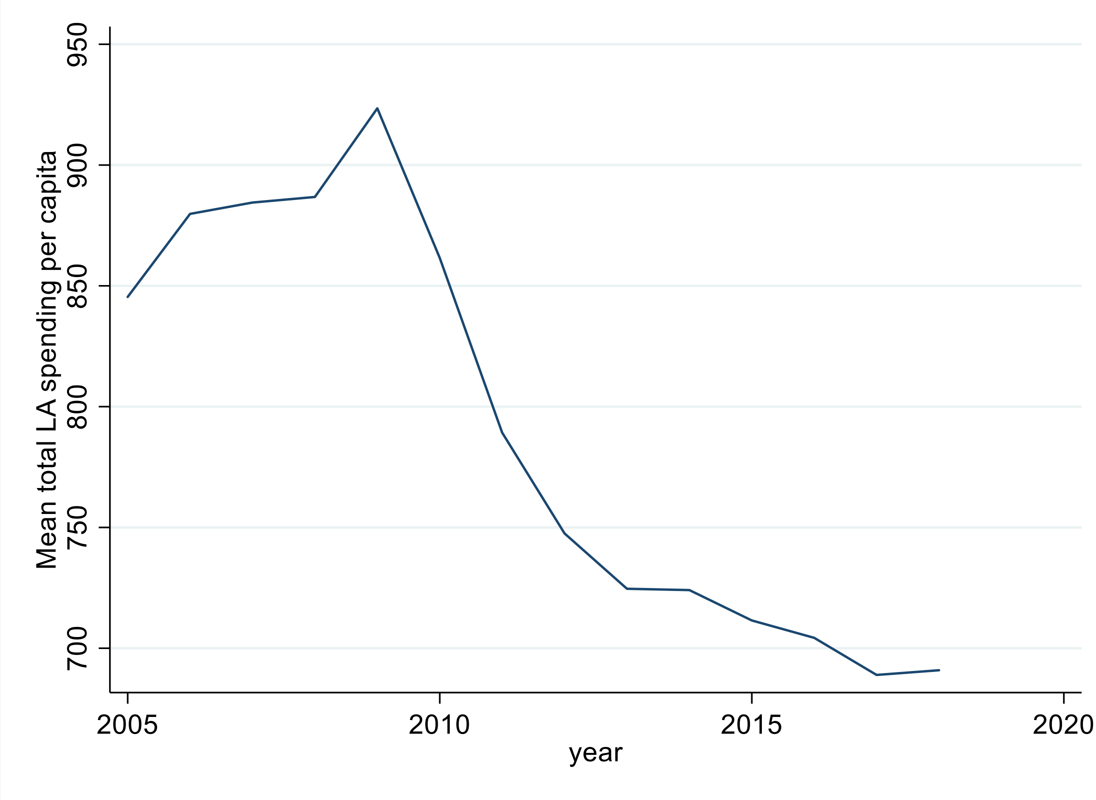

Appendix 3: Impact of LA service spending on total nutritional anaemia hospital admissions, shown as the Incident Rate Ratio and stratified by gender (95% Confidence Intervals in brackets)

	Incidence Rate Ratio for total nutritional anaemia hospital admissions by LA with an £100 increase in total LA service spending		
	All Ages	Male	Female
Full Sample[1]	0.981 (0.964, 0.999) p=0.038	0.975 (0.957, 0.993) p=0.007	0.992 (0.974, 1.010) p=0.358
IMD[2]			
1 (most deprived)	0.974 (0.951, 0.998) p=0.032	0.968 (0.944, 0.993) p=0.012	0.980 (0.956, 1.005) p=0.118
2	0.991 (0.949, 1.035) p=0.678	0.988 (0.947, 1.030) p=0.563	1.005 (0.962, 1.050) p=0.818
3	1.016 (0.976, 1.058) p=0.431	1.009 (0.962, 1.058) p=0.707	1.032 (0.989, 1.076) p=0.151
4	0.883 (0.831, 0.939) p<0.001	0.872 (0.817, 0.932) p<0.001	0.902 (0.851, 0.956) p<0.001
5 (least deprived)	1.011 (0.955, 1.070) p=0.712	1.001 (0.936, 1.071) p=0.967	1.029 (0.967, 1.094) p=0.367
Level of reductions to working age benefits per capita			
1 (lowest reductions)	1.011 (0.961, 1.063) p=0.680	1.013 (0.962, 1.067) p=0.623	1.020 (0.962, 1.083) p=0.506
2	0.963 (0.935, 0.991) p=0.010	0.955 (0.926, 0.985) p=0.004	0.981 (0.951, 1.011) p=0.210
3	0.992 (0.952, 1.032) p=0.671	0.983 (0.944, 1.024) p=0.419	0.999 (0.960, 1.040) p=0.960
4 (greatest reductions)	0.975 (0.949, 1.003) p=0.077	0.970 (0.942, 1.000) p=0.048	0.986 (0.957, 1.015) p=0.330

[1] Adjusted by percentage working age, percentage male, GDHI and unemployment rate.

[2] Index of Multiple Deprivation, quintiles based on relative ranking of LAs

Appendix 4: Association between LA service spending and principal nutritional anaemia hospital admissions, shown as the Incident Rate Ratio and stratified by age (95% Confidence Intervals in brackets).

	Incidence Rate Ratio for principal nutritional anaemia hospital admissions by LA with an £100 increase in total LA service spending			
	All Ages	0-14	15-64	65+
Full sample[1]	0.987 (0.963, 1.011) p=0.293	0.996 (0.945, 1.051) p=0.896	0.986 (0.957, 1.015) p=0.332	0.983 (0.957, 1.009) p=0.190
IMD[2]				
1 (most deprived)	0.985 (0.950, 1.022) p=0.435	0.969 (0.899, 1.043) p=0.400	0.989 (0.945, 1.035) p=0.634	0.978 (0.941, 1.016) p=0.247
2	0.988 (0.942, 1.036) p=0.611	0.984 (0.866, 1.119) p=0.806	1.000 (0.950, 1.053) p=0.999	0.975 (0.928, 1.024) p=0.311
3	1.031 (0.973, 1.092) p=0.308	1.086 (0.895, 1.319) p=0.402	1.051 (0.989, 1.116) p=0.107	1.002 (0.929, 1.082) p=0.951
4	0.838 (0.759, 0.925) p<0.001	1.255 (1.009, 1.560) p=0.041	0.798 (0.715, 0.892) p<0.001	0.853 (0.769, 0.946) p=0.003
5 (least deprived)	1.040 (0.957, 1.130) p=0.357	1.135 (0.943, 1.365) p=0.180	1.013 (0.929, 1.104) p=0.778	1.053 (0.968, 1.146) p=0.226
Level of reductions to working age benefits per capita				
1 (lowest reductions)	1.041 (0.972, 1.115) p=0.252	1.205 (1.001, 1.451) p=0.049	1.019 (0.949, 1.095) p=0.601	1.052 (0.977, 1.132) p=0.180
2	0.967 (0.927, 1.009) p=0.125	1.002 (0.835, 1.203) p=0.983	0.959 (0.910, 1.010) p=0.113	0.968 (0.927, 1.012) p=0.148
3	1.000 (0.949, 1.054) p=0.995	1.030 (0.919, 1.153) p=0.616	1.016 (0.965, 1.070) p=0.548	0.978 (0.920, 1.040) p=0.479
4 (greatest reductions)	0.980 (0.947, 1.014) p=0.242	0.975 (0.916, 1.038) p=0.430	0.979 (0.937, 1.022) p=0.333	0.975 (0.943, 1.008) p=0.140

[1] Adjusted by percentage working age, percentage male, GDHI and unemployment rate.

[2] Index of Multiple Deprivation, quintiles based on relative ranking of LAs

Appendix 5: Rates of principal and total nutritional anaemia hospital admissions over time, excluding those alongside other conditions which may cause nutritional deficiencies, stratified by age (2005-2018)

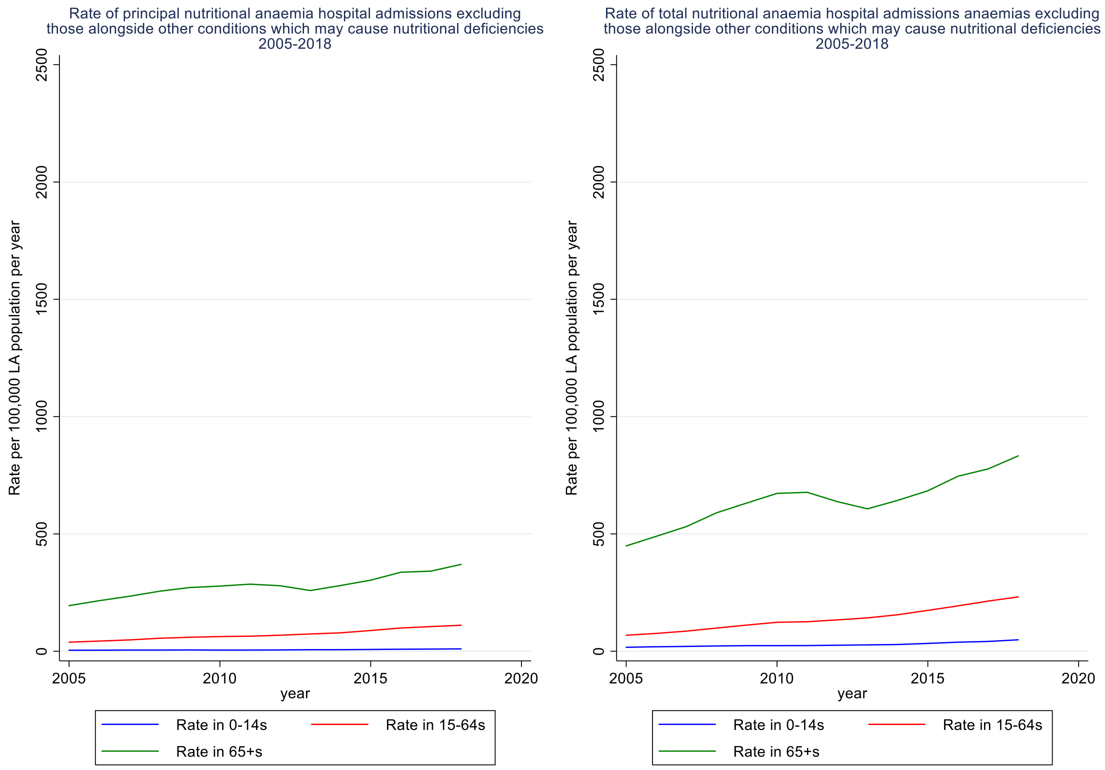

Appendix 6: Association between LA service spending and total nutritional anaemia hospital admissions excluding those alongside a principal or secondary diagnosis of another condition which may cause a nutritional deficiency, shown as the Incident Rate Ratio and stratified by age (95% Confidence Intervals in brackets)

	Incidence Rate Ratio for total nutritional anaemia hospital admissions by LA with an £100 increase in total LA service spending			
	All Ages	0-14	15-64	65+
Full sample[1]	0.991 (0.974, 1.009) p=0.312	0.967 (0.894, 1.047) p=0.408	0.989 (0.969, 1.009) p=0.279	0.998 (0.980, 1.017) p=0.829
IMD[2]				
1 (most deprived)	0.985 (0.962, 1.009) p=0.208	0.933 (0.823, 1.058) p=0.279	0.981 (0.952, 1.011) p=0.207	0.994 (0.969, 1.020) p=0.658
2	1.001 (0.961, 1.044) p=0.950	1.047 (0.989, 1.109) p=0.118	1.009 (0.967, 1.052) p=0.692	1.002 (0.955, 1.050) p=0.945
3	1.034 (0.998, 1.072) p=0.067	1.025 (0.934, 1.125) p=0.599	1.041 (0.991, 0.093) p=0.110	1.029 (0.992, 1.066) p=0.128
4	0.876 (0.818, 0.938) p<0.001	0.931 (0.789, 1.098) p=0.396	0.850 (0.786, 0.920) p<0.001	0.888 (0.828, 0.953) p=0.001
5 (least deprived)	1.030 (0.968, 1.095) p=0.357	0.977 (0.818, 1.167) p=0.799	1.024 (0.959, 1.094) p=0.472	1.038 (0.976, 1.104) p=0.237
Level of reductions to working age benefits per capita				
1 (lowest reductions)	1.026 (0.968, 1.087) p=0.390	0.979 (0.834, 1.149) p=0.792	1.031 (0.976, 1.088) p=0.279	1.027 (0.963, 1.096) p=0.412
2	0.976 (0.947, 1.005) p=0.104	0.973 (0.906, 1.045) p=0.446	0.960 (0.924, 0.997) p=0.035	0.993 (0.964, 1.023) p=0.656
3	1.004 (0.964, 1.047) p=0.839	1.030 (0.919, 1.154) p=0.616	1.012 (0.970, 1.055) p=0.590	1.002 (0.956, 1.050) p=0.946
4 (greatest reductions)	0.981 (0.956, 1.007) p=0.150	0.925 (0.813, 1.052) p=0.234	0.978 (0.946, 1.012) p=0.198	0.988 (0.963, 1.013) p=0.326

[1] Adjusted by percentage working age, percentage male, GDHI and unemployment rate.

[2] Index of Multiple Deprivation, quintiles based on relative ranking of LAs

Appendix 7: Association between LA service spending excluding social care spending and total nutritional anaemia hospital admissions, shown as the Incident Rate Ratio and stratified by age (95% Confidence Intervals in brackets)

	Incidence Rate Ratio for total nutritional anaemia hospital admissions by LA with an £100 increase in total LA service spending			
	All Ages	0-14	15-64	65+
Full Sample[1]	0.960 (0.939, 0.982) p<0.001	0.960 (0.869, 1.061) p=0.425	0.958 (0.934, 0.983) p=0.001	0.958 (0.936, 0.980) p<0.001
IMD[2]				
1 (most deprived)	0.957 (0.927, 0.987) p=0.006	0.903 (0.769, 1.060) p=0.213	0.950 (0.916, 0.986) p=0.007	0.962 (0.931, 0.995) p=0.025
2	0.976 (0.935, 1.020) p=0.278	1.074 (1.020, 1.131) p=0.006	0.989 (0.944, 1.037) p=0.652	0.962 (0.919, 1.008) p=0.104
3	0.988 (0.934, 1.045) p=0.670	1.087 (0.973, 1.214) p=0.141	0.990 (0.927, 1.056) p=0.750	0.973 (0.919, 1.030) p=0.349
4	0.847 (0.796, 0.901) p<0.001	0.959 (0.803, 1.146) p=0.645	0.822 (0.760, 0.888) p<0.001	0.849 (0.799, 0.901) p<0.001
5 (least deprived)	1.003 (0.931, 1.081) p=0.934	1.031 (0.889, 1.195) p=0.687	1.005 (0.924, 1.092) p=0.916	0.997 (0.933, 1.065) p=0.934
Level of reductions to working age benefits per capita				
1 (lowest reductions)	0.982 (0.914, 1.055) p=0.612	0.997 (0.845, 1.177) p=0.974	0.984 (0.910, 1.065) p=0.695	0.975 (0.912, 1.042) p=0.457
2	0.940 (0.903, 0.979) p=0.003	0.957 (0.874, 1.047) p=0.339	0.936 (0.891, 0.982) p=0.007	0.937 (0.900, 0.975) p=0.001
3	0.961 (0.922, 1.002) p=0.062	1.025 (0.912, 1.152) p=0.685	0.966 (0.924, 1.009) p=0.123	0.951 (0.909, 0.994) p=0.027
4 (greatest reductions)	0.963 (0.930, 0.997) p=0.031	0.911 (0.774, 1.072) p=0.261	0.959 (0.921, 0.999) p=0.043	0.965 (0.932, 1.000) p=0.047

[1] Adjusted by percentage working age, percentage male, GDHI and unemployment rate.

[2] Index of Multiple Deprivation, quintiles based on relative ranking of LAs
