# Supplementary material for: Local area public sector spending and nutritional anaemia hospital admissions in England: a longitudinal ecological study
Source: BMJ Open. 2022 Sep 28;12(9):e059739. doi: 10.1136/bmjopen-2021-059739 (PMC9528630; doi:10.1136/bmjopen-2021-059739)
Supplement: Author's manuscript [file bmjopen-2021-059739.draft_revisions.pdf]

BMJ Open

BMJ Open is committed to open peer review. As part of this commitment we make the peer review history of every article we publish publicly available.

When an article is published we post the peer reviewers' comments and the authors' responses online. We also post the versions of the paper that were used during peer review. These are the versions that the peer review comments apply to.

The versions of the paper that follow are the versions that were submitted during the peer review process. They are not the versions of record or the final published versions. They should not be cited or distributed as the published version of this manuscript.

BMJ Open is an open access journal and the full, final, typeset and author-corrected version of record of the manuscript is available on our site with no access controls, subscription charges or pay-per-view fees (http://bmjopen.bmj.com).

If you have any questions on BMJ Open's open peer review process please email info.bmjopen@bmj.com

BMJ Open

Local area public sector spending and nutritional anaemia hospital admissions in England: a longitudinal ecological study

Journal:	*BMJ Open*
Manuscript ID	bmjopen-2021-059739
Article Type:	Original research
Date Submitted by the Author:	02-Dec-2021

Complete List of Authors:	Jenkins, Rosemary; Imperial College London<br>Vamos, Eszter; Imperial College London, School of Public Health<br>Mason, Kate; University of Liverpool, Public Health & Policy<br>Daras, Konstantinos ; University of Liverpool, Public Health, Policy and Systems<br>Taylor-Robinson, David; University of Liverpool,<br>Bambra, Clare; Newcastle University<br>Millett, Christopher; Imperial College, Primary Care and Social Medicine<br>Laverty, Anthony; Imperial College London, Primary Care and Public Health

Keywords:	SOCIAL MEDICINE, PUBLIC HEALTH, Health policy < HEALTH SERVICES ADMINISTRATION & MANAGEMENT

SCHOLARONE™
Manuscripts

BMJ

I, the Submitting Author has the right to grant and does grant on behalf of all authors of the Work (as defined in the below author licence), an exclusive licence and/or a non-exclusive licence for contributions from authors who are: i) UK Crown employees; ii) where BMJ has agreed a CC-BY licence shall apply, and/or iii) in accordance with the terms applicable for US Federal Government officers or employees acting as part of their official duties; on a worldwide, perpetual, irrevocable, royalty-free basis to BMJ Publishing Group Ltd ("BMJ") its licensees and where the relevant Journal is co-owned by BMJ to the co-owners of the Journal, to publish the Work in this journal and any other BMJ products and to exploit all rights, as set out in our licence.

The Submitting Author accepts and understands that any supply made under these terms is made by BMJ to the Submitting Author unless you are acting as an employee on behalf of your employer or a postgraduate student of an affiliated institution which is paying any applicable article publishing charge ("APC") for Open Access articles. Where the Submitting Author wishes to make the Work available on an Open Access basis (and intends to pay the relevant APC), the terms of reuse of such Open Access shall be governed by a Creative Commons licence – details of these licences and which Creative Commons licence will apply to this Work are set out in our licence referred to above.

Other than as permitted in any relevant BMJ Author's Self Archiving Policies, I confirm this Work has not been accepted for publication elsewhere, is not being considered for publication elsewhere and does not duplicate material already published. I confirm all authors consent to publication of this Work and authorise the granting of this licence.

For peer review only

Local area public sector spending and nutritional anaemia hospital admissions in England: a longitudinal ecological study

Rosemary H. Jenkins[1], Eszter P Vamos[1], Kate E. Mason[2], Konstantinos Daras[2], David Taylor-Robinson[2], Clare Bambra[3], Christopher Millett[1], Anthony A. Laverty[1]

[1] Department of Primary Care and Public Health, School of Public Health, Imperial College London, Charing Cross Campus; The Reynolds Building; St Dunstan's Road; London, UK; W6 8RP.

[2] Department of Public Health, Policy and Systems, Institute of Population Health, University of Liverpool; Waterhouse Building Block F, 2nd Floor, Liverpool, UK; L69 3BX

[3] Population Health Sciences Institute, Faculty of Medical Sciences, Newcastle University, UK, NE1 4LE

Correspondence to: Dr Anthony Laverty, Public Health Policy Evaluation Unit, Department of Primary Care and Public Health, School of Public Health, Imperial College London, Charing Cross Campus; The Reynolds Building; St Dunstan's Road; London, UK; W6 8RP. a.laverty@imperial.ac.uk

ABSTRACT

Introduction: Reductions in local government spending may have impacts on diets and health which increase the risk of hospital admissions for nutritional anaemias. Mechanisms include potential impacts of changes to local authority (LA) services (e.g. housing services) on personal resources and food access, availability, and provision. We therefore investigated the association between changes in LA spending and nutritional anaemia related hospital admissions.

Design: Longitudinal analysis of LA panel data using Poisson fixed effects regression models.

Setting: 312 LAs in England (2005-2018).

Main exposure: Total LA service expenditure per capita per year.

Main outcome: Principal and total nutritional anaemia hospital admissions, for all ages and stratified by age (0-14, 15-64, 65+).

Results: LA service expenditure increased by 9% between 2005 and 2009 then decreased by 20% between 2010 and 2018. Total nutritional anaemia hospital admissions increased between 2005 and 2018 from 173 to 633 admissions per 100,000 population. A £100 higher LA service spending was associated with a 1.9% decrease in total nutritional anaemia hospital admissions (adjusted Incidence Rate Ratio: 0.98, 95% CI 0.96 to 0.99). When stratified by age, this was seen only in adults. A £100 higher LA service spending was associated with a 2.6% decrease in total nutritional anaemia hospital admissions in the most deprived LAs (aIRR: 0.97, 95% CI: 0.95 to 1.0).

Conclusion: Increased LA spending was associated with reduced hospital admissions for nutritional anaemia. Austerity related reductions had the opposite effect, increasing admissions, with greater

For peer review only

impacts in more deprived areas. This adds further evidence to the potential negative impacts of austerity policies on health and health inequalities. Re-investing in LA services may prevent hospital admissions associated with nutritional anaemias.

Strengths and limitations of this study

- This study uses national data to assess relationships between public sector spending and hospital admissions
- A fixed effect panel design allows us to examine within local authority changes in both exposures and outcomes
- However, we examined hospitalisations only, which does not give a full picture of burden of disease, as measures such as GP diagnoses would
- The natural experimental design means that we cannot infer causality and research at the individual level would assist with this in the future

For peer review only

INTRODUCTION

Nutritional anaemias related to poor diet are an important public health issue, increasing in prevalence over recent years. Nutritional anaemias involve reductions in red blood cells or their haemoglobin content due to a low nutritional intake of certain micronutrients, particularly iron, vitamin B-12, and folate(1). Nutritional anaemias are associated with a range of adverse health outcomes – for example, iron deficiency anaemia can lead to poor outcomes in those with coronary heart disease(2). Furthermore, nutrient deficiencies, which cause nutritional anaemias, are important risk factors for diseases through multiple physiological mechanisms(3, 4). For example, hospitalisations for iron deficiency anaemias increased from 67,592 in 2008-09 to 131,064 in 2016-17(5). A 2019 report by the UK House of Commons Environmental Audit Committee described the presence of a double burden of malnutrition in the UK, with overweight and obesity coexisting with undernutrition, both leading to micronutrient deficiencies(6).

The increase in hospital admissions for nutritional anaemias has occurred at the same time as large cuts to public services in the UK(5). In 2010, the UK government introduced austerity policies which led to considerable changes to the UK benefits system and reductions in local authority (LA) funding and service expenditure(7, 8). Reductions in LA funding had greater impacts in more deprived and urban councils more, especially in the north of England, leading to differential impacts on the wide range of services that LAs provide(9-11). These services include social care, housing, highways and transport, environment and regulatory, and planning and development services(11). Systematic review evidence demonstrates that these services may be important in improving the social determinants of health and health inequalities(12). Research reports have found that reductions to LA service spending may have had negative impacts on health and widened health inequalities(13). For example, a £100 decrease in annual LA funding per person has been associated with a decrease in life expectancy at birth of 1.2-1.3 months between 2013-17(14).

The relationship between changes in local government spending and risk of nutritional anaemia is unclear. Much of the extant research on nutritional impacts of austerity policies in the UK has focused on foodbank use and not other potential outcomes such as hospital admissions(15). Risk of nutritional anaemias caused by poor diets may be plausibly impacted by changes to public sector spending through a range of different mechanisms (Figure 1). For example, reductions in social care spending may affect financial security and food acquisition in vulnerable people and may change the quality of food provision in domiciliary care and through services such as Meals on Wheels and lunch clubs(16, 17). Reductions in housing service expenditure may also reduce personal resources(18, 19). We therefore aimed to investigate the association between changes to LA service spending and

For peer review only

principal and total nutritional anaemia hospital admissions in LAs in England. Given the role of LAs in addressing the social determinants of health, we hypothesise that effects may be stronger in more deprived LAs and those experiencing greater reductions in working age benefits.

For peer review only

METHODS

Data

Exposure variables

Annual LA service expenditure is reported by the Ministry of Housing, Communities & Local Government, compiled and freely available from the Place-based Longitudinal Data Resource (PLDR) for 326 LAs (20). We used net expenditure data for lower tier LAs, which excludes income from services. The Islands of Scilly and the City of London were excluded due to small populations, and an additional 12 LAs were excluded due to missing covariate data. We calculated total net LA expenditure excluding police services and fire and rescue services (as these are funded by separate grants and precepts and provided by separate authorities), education services (due to changes in schooling provision over the study period), public health services (due to data not being available for all years due to changes in LA responsibilities) and court services (due to data not being available for all years)(21). All data were adjusted for inflation using the Consumer Price Index with 2015 as the reference year (22).

Outcome variables

Hospital Episode Statistics (HES) is a database of all NHS hospitals' admissions, A&E attendances, and outpatient appointments in England(23). For this study, we used yearly LA-level counts of inpatient or day-case admissions of nutritional anaemias in NHS hospitals available as safeguarded datasets from the PLDR (20). These were iron deficiency anaemia (excluding iron deficiency anaemia due to blood loss), vitamin B-12 deficiency anaemia, folate deficiency anaemia and other nutritional anaemias by LA. We obtained principal nutritional anaemia hospital admissions data where the nutritional anaemia was the "condition established after study to be chiefly responsible for occasioning the admission of the patient to the hospital for care"(24). We also obtained data for hospital admissions where nutritional anaemias are a secondary diagnosis, an additional condition that an individual may have during a hospital admission for a different principal diagnosis. These were given by each age (0-14, 15-64, 65+) or sex group individually. We aggregated these data to obtain principal nutritional anaemia hospital admissions and total nutritional anaemia hospital admissions (consisting of both principal and secondary diagnoses). Because principal nutritional anaemia hospital admissions are relatively uncommon, total nutritional anaemia hospital admissions are more reflective of the burden of nutritional anaemias in the population. We used annual LA population estimates from the Office for National Statistics (ONS) to generate admission rates per 100,000 population(25).

For peer review only

For sensitivity analyses, we also obtained counts of nutritional anaemia admissions excluding those alongside a principal or secondary diagnosis of another condition that may cause nutritional deficiencies, such as cachexia, malabsorption, malignancies and diabetes (full list in Appendix 1). As with the main outcome variable above, these data were obtained stratified by age or gender. These data were also aggregated to obtain principal and total (ie. principal and secondary) nutritional anaemia hospital admissions.

Covariates & Effect Modifiers

In the regression models we adjusted for the proportion of the LA population that is working age, proportion of LA population that is male, Gross Disposable Household Income (GDHI) and unemployment rate. The proportion of LA population that was working age (aged 15-64) and male were calculated from ONS data (25). These covariates were included to account for differences in changing LA demographics. We obtained GDHI data, an area-level measure of individuals' available money for spending or saving following payment of taxes and receipt of benefits, adjusted for inflation, from the ONS(26). LA unemployment rates were also obtained from the ONS, who calculate them based on a model utilising estimates from the Labour Force Survey and counts of people claiming unemployment benefits(27). GDHI and unemployment rate were used as markers of available household resources at the LA level. Time invariant factors were not adjusted for as they were removed by the fixed effects model, however, we also obtained Index of Multiple Deprivation (IMD) and level of reductions in working-age benefits as they were identified a priori as potential effect modifiers. IMD 2015 was obtained from the Ministry of Housing, Communities & Local Government(28). We used the population-weighted rank of average rank of LA IMD to make quintiles of relative deprivation. We also used the Uneven Impact of Welfare Reform dataset (provided by the authors) which estimated the cumulative reductions in working-age people's benefits for each LA due to welfare reforms since 2010(29). Previous research has found strong associations between reductions in welfare benefits and clinical outcomes(30). We derived quartiles of this variable to assess level of reductions to working-age benefits as a potential effect modifier.

Analyses

We descriptively examined total LA service expenditure in 2005, 2010, and 2018, including differences by covariates and proposed effect modifiers. We also calculated percentage changes before (2005-2009) and after (2010-2018) the introduction of austerity measures. We tabulated mean principal and total nutritional anaemia hospital admissions for the full sample and stratified by age group, presented as rates per 100,000 population, and plotted their change over time. We also

For peer review only

assessed the relationship between change in LA service spending and change in principal and total nutritional anaemia hospital admissions between 2005-10 and 2010-18 using scatter plots.

We used a Poisson fixed effects panel regression approach for the main analysis. Panel regression models can be used to investigate LA units over time while taking data clustering over time into account(31). Poisson fixed effects regression was used as a robust approach to modelling count panel data(32). We used cluster robust standard errors to adjust for potential autocorrelation and heteroskedasticity(33). We also created dummy variables for each year and used them to account for England-wide time effects. LA population was used as an offset variable to account for population size. For our primary analysis, we undertook a Poisson fixed effects analysis to investigate the relationship between total LA service spending and total nutritional anaemia hospital admissions. We present adjusted models (with covariates as proportion of the LA population that is working age, proportion of the LA population that is male, Gross Disposable Household Income (GDHI) and unemployment rate). We also subsequently stratified by IMD quintiles and level of benefit reductions quartiles to test a priori hypotheses regarding effect modification. We undertook analyses stratified by age and gender. We separately analysed principal nutritional anaemia hospital admissions as the outcome, with adjusted and stratified Poisson models as above.

We also undertook two sensitivity analyses. In the first, we used as the outcome variable total nutritional anaemia hospital admissions excluding admissions alongside a diagnosis of another condition which may cause a nutritional deficiency. While public sector spending reductions could cause or exacerbate this wider set of conditions, this sensitivity analysis focuses on diagnoses of being admitted *for* nutritional anaemia rather than *with* it. In the second sensitivity analysis, we excluded social care expenditure from the exposure variable. Unlike most areas of LA service expenditure, some LAs had to increase their social care expenditure after 2010 due to demand. Thus, LAs with higher spending on social care may have higher levels of nutritional anaemias, which may lead to issues of reverse causality when social care is included in the exposure variable(34). Our sensitivity analysis investigated the relationship between LA spending excluding social care and total nutritional anaemia hospital admissions.

Patient and Public Involvement

Plans for this research were presented to a committee of three public experts in November 2020. The public experts provided feedback on the research plans and appropriate changes were made following the meeting.

For peer review only

RESULTS

On average, LA service spending increased by 9% between 2005 and 2009, and then decreased by 20% between 2010 and 2018 (Table 1). Mean total LA spending per year is plotted in Appendix 2. Between 2005 and 2009, LAs in Yorkshire and the Humber (16%), the East Midlands (15%), and the North West (14%) increased expenditure the most, while London (4%) and the South East (5%) increased expenditure the least. However, LAs with the highest unemployment rates decreased expenditure between 2005-2009. Following 2010, London had the greatest decrease of 31%, followed by the Northern regions (a 27% decrease for the North East and 21% for both the North West and Yorkshire and the Humber). More deprived LAs experienced greater cuts (28% in the most deprived quintile compared to 12% for the least deprived quintile). Greater reductions were also seen in areas with higher unemployment rates and larger reductions to working age benefits.

Total nutritional anaemia hospital admissions have increased since 2005, from 173 per 100,000 population in 2005 to 632 per 100,000 population in 2018 (Fig. 2). Total nutritional anaemia hospital admissions in 65+ adults increased between 2005-2010, with the rate of increase slowing between 2010-2013, and then increasing more steeply between 2014 and 2018. Rate of admissions in 65+s was 684 per 100,000 population in 2005 and 2,103 per 100,000 in 2018. There was also an increase throughout the study period for 15-64-year olds, from 88 per 100,000 population in 2005 to 364 per 100,000 population. Total nutritional anaemia hospital admissions also increased slightly in 0-14s between 2005 and 2018, from 19 to 57 admissions per 100,000 population.

In 2018, the rate of principal nutritional anaemia hospital admissions was 236 per 100,000 population (Table 2). Rates increased with age (11 per 100,000 population for 0-14s, 154 for 15-64s, and 717 for 65+s). The rate of total nutritional anaemia hospital admissions in 2018 was 632 per 100,000 population. Rates of total nutritional anaemia hospital admissions also increased with age – rates were relatively low in children (57 per 100,000 population) but were 364 and 2,102 per 100,000 population in adults (15-64 and 65+ respectively). Principal and total nutritional anaemia hospital admissions increased with deprivation for all age groups. Rates of admissions increased with unemployment and level of benefit reductions and decreased with GDHI.

For peer review only - http://bmjopen.bmj.com/site/about/guidelines.xhtml

Figure 3 shows change in LA service spending before and after austerity measures were announced, plotted with changes in principal and total nutritional anaemia hospital admissions. The x axes represent change in admissions and the y axes represent change in LA spending. For both principal and total nutritional anaemia hospital admissions, this relationship was flat between 2005 and 2009. There was a slight negative relationship for change in LA spending and change in principal nutritional anaemia hospital admissions, such that a greater decrease in LA service spending was related to a greater increase in nutritional anaemias. This relationship was steeper for total nutritional anaemia hospital admissions especially for the 15-64 and 65+ age groups.

Poisson fixed effects analysis of the association between total LA service spending and principal and total nutritional anaemia hospital admissions

A £100 per capita higher LA service spending was associated with 1.9% lower total nutritional anaemia hospital admissions (adjusted IRR: 0.981, 95% CI: 0.964, 0.999) (Table 3). This relationship was only statistically significant in adults aged ≥15 years. A £100 higher LA service spend per capita was associated with 2.2% lower total nutritional anaemia hospital admissions in the adjusted model for people aged 15-64, and 2.1% lower rates in 65+ respectively (IRR for 15-64: 0.978, 95% CI: 0.959, 0.998; IRR for 65+: 0.979, 95% CI: 0.960, 0.997).

When stratified by IMD, this relationship was statistically significant in the 1st (most deprived) (adjusted IRR for all age groups: 0.974, 95% CI: 0.951, 0.998) and 4th (adjusted IRR for all age groups: 0.883, 95% CI: 0.831, 0.939) quintiles. This persisted in adults. When stratified by level of reductions to working age benefits, this relationship was present in the 2nd quartile (adjusted IRR for all age groups: 0.963, 95% CI: 0.935, 0.991) and 4th quartile for 65+ (adjusted IRR for 65+: 0.973, 95% CI: 0.947, 0.999). Similar results were found when stratified by gender, although results were only statistically significant for males (Appendix 3).

When we investigated this relationship with principal rather than total nutritional anaemia hospital admissions as the outcome, this relationship was no longer statistically significant (adjusted IRR: 0.987, 95% CI: 0.963, 1.011) (Appendix 4). LA spending was not statistically significantly associated with principal nutritional anaemia hospital admissions in any of the age groups: 0-14 (adjusted IRR: 0.996, 95% CI: 0.945, 1.051), 15-64 (adjusted IRR: 0.986, 95% CI: 0.957, 1.015), and 65+ (adjusted IRR: 0.983, 95% CI: 0.957, 1.009). When stratified by IMD, a statistically significant relationship was present for the 4th quintile only (eg. IRR for all ages: 0.838, 95% CI: 0.759, 0.925).

Sensitivity analysis: excluding diagnoses which may cause a nutritional deficiency

For peer review only

The increase in nutritional anaemias over time (Fig. 2) was much less steep when nutritional anaemia admissions alongside a diagnosis of a condition which may cause a nutritional deficiency were excluded (Appendix 5). When nutritional anaemia hospital admissions that were alongside a principal or secondary diagnosis of another condition which may cause a nutritional deficiency were excluded in the Poisson regression analysis, generally the relationship between LA spending and total nutritional anaemia hospital admissions was not statistically significant (for example, adjusted IRR for all age groups: 0.991, 95% CI: 0.974, 1.009) (Appendix 6). There were some significant relationships in the middle IMD and level of reductions to working age benefits (eg. adjusted IRR for quintile 2 of reductions to working age benefits for 15-64: 0.960, 95% CI: 0.924, 0.997).

Sensitivity analysis: total LA service expenditure excluding social care

When social care was excluded from the exposure variable, a £100 increase in LA service spending was associated with a 4.0% decrease in total nutritional anaemia hospital admissions (adjusted IRR: 0.960, 95% CI: 0.939, 0.982) (Appendix 7). This relationship remained in the 15-64 age group (adjusted IRR for 15-64: 0.958, 95% CI: 0.934, 0.983) and the 65+ age group (adjusted IRR for 65+: 0.958, 95% CI: 0.936, 0.980) when stratified by age. Statistically significantly greater reductions in nutritional anaemias were seen in the first and fourth IMD quintiles, suggesting effect modification by deprivation. Little effect modification was seen by level of reductions to working age benefits.

DISCUSSION

We assessed the relationship between LA service spending and risk of hospital admissions related to nutritional anaemias in England and found that reductions in spending related to austerity may have increased nutritional anaemia hospital admissions. LA service spending increased by 9% between 2005 and 2009 then decreased by 20% between 2010 and 2018, following the introduction of austerity policies in 2010. Total nutritional anaemia hospital admissions also increased between 2005 and 2018. We found that a £100 higher LA service spending was associated with 1.9% lower total nutritional anaemia hospital admissions. This was driven by admissions in adults. A 2.6% lower admission rate was seen with a £100 higher expenditure in the most deprived LAs, suggesting greater impacts by deprivation, especially since these LAs experienced greater service spending reductions overall (a 28% reduction was seen in the most deprived quintile compared to a 12% reduction in the least deprived quintile between 2010 and 2018). However, we also found that a £100 higher expenditure was statistically significantly associated with a reduction in admissions in quintile 4 (one of the least deprived quintiles), suggesting that there may be impacts across the spectrum of LAs in terms of deprivation. Regardless of the relative effects of spending across levels

For peer review only

of deprivation, the far greater cuts to spending in more deprived areas mean the absolute impact on nutritional anaemias will have been felt disproportionately in these areas.

We found that total nutritional anaemia hospital admissions increased between 2005 and 2018. This aligns with previous research suggesting decreases in micronutrient consumption and corresponding increases in nutritional anaemias in the UK(5, 35, 36). We identified an overall increase in total nutritional anaemia hospital admissions which plateaued between 2010-2013, and then increased again. Research has found that between 2007 and 2011 the percentage of patients known to have had nutritional screening increased from 67-78% to 86-95%(37). Therefore, the increase in nutritional anaemia hospital admissions between 2005-2010 may be an artefact of increased screening over this period, with the plateau over the following years representing nutritional screening reaching near-saturation of hospital admissions. Given that nutritional screening was approaching saturation as austerity was being rolled out, an increase in nutritional anaemias after 2010 - during austerity - may be in part explained by changes in LA spending as per our hypothesis.

Our study suggests that these decreases in LA service spending may have health impacts. This supports other studies which suggest that decreases in LA service spending may adversely affect health and mortality(14, 38). Our research adds new evidence that there may be associations with nutritional anaemias in addition to other health outcomes previously examined. As nutritional anaemias are important risk factors for diseases, this may be one pathway through which reductions in LA service expenditure may be associated with health. More deprived areas also experienced greater reductions in LA service spending, meaning the absolute increase in nutritional deficiencies attributable to service spending cuts will have been greater in more deprived areas, which already have greater health needs. This aligns with other evidence that austerity measures had a greater impact in more deprived areas and may have widened health inequalities due to the role of LA services in improving the social determinants of health(9, 10, 12, 13).

This research has several strengths. We are the first to examine associations between austerity policies and nutritional anaemia hospital admissions. We used a large panel dataset with coverage of NHS hospitals for 312 LAs in England, over a long time period (2005-2018). We used a fixed effects modelling strategy and were able to account for all time-invariant factors that differ between LAs and changing population sizes, allowing us to assess within-LA change in hospital admissions over time. Furthermore, our sensitivity analysis found a stronger relationship between total LA service spending and total nutritional anaemia hospital admissions when social care spending was excluded. This suggests that social care spending may be blunting the relationship between LA service spending and total nutritional anaemia hospital admissions, likely due to higher levels of nutritional

For peer review only

anaemias requiring higher levels of social care spending(34). This further supports our hypothesis that austerity measures may have led to increased nutritional anaemia hospital admissions.

Our study also has some limitations. As previously described, we used counts of nutritional anaemia hospital admissions, which may be an artefact of hospital processes such as more screening leading to more diagnoses. As we used data on admissions, we did not examine diagnoses by GPs, which may give a more complete picture of potential impacts on nutritional anaemias at the population level. Furthermore, the relationship we have described was only statistically significant when total nutritional anaemia hospital admissions were included as the outcome measure and not when principal nutritional anaemia hospital admissions were the outcome. Therefore, it is possible that the relationship seen may be a result of LA service spending being associated with increases in people accessing hospitals for other health conditions and nutritional anaemias also being present, leading to more total nutritional anaemia hospital admissions which would otherwise have been in the community and undiagnosed. One possibility is that such increases in hospital access may be due to more general impacts of austerity policies on health outcomes. Additionally, our exposure data were per financial year and our outcome data were per calendar year, representing a potential mismatch in time periods assessed.

Sensitivity analyses excluding hospital admissions alongside a medical condition which may cause a nutritional deficiency did not identify a relationship between LA service spending and total nutritional anaemia hospital admissions. This suggests that the relationship between LA spending and nutritional anaemia hospital admissions is driven by changes in the prevalence of these other conditions. The degree to which nutritional anaemias may be caused by other medical conditions is difficult to assess in routine data without more detailed clinical parameters and we excluded a range of common conditions such as cancers and type 2 diabetes where nutritional deficiencies are common. These diseases have a nutritional component, particularly with regards to their association with obesity, and thus may represent wide-ranging impacts of austerity policies on nutrition in terms of both nutritional anaemias and chronic disease. Together our analyses suggest that the burden of nutritional anaemias is increasing over time and that this is more apparent in areas with greater levels of deprivation, greater levels of medical comorbidity, and potentially in areas with more severe reductions in LA spending(39).

Our panel study suggests that reductions in LA service spending due to UK austerity policies may be associated with an increase in nutritional anaemias. This adds further evidence to the growing evidence base regarding potential impacts of these policies on health and health inequalities. Nutritional anaemias have wide-ranging impacts on health. Thus, policy-makers should consider

For peer review only

associations between fiscal policies and health prior to their introduction, particularly given the budget deficit – and possible further reductions in LA budgets alongside tax rises - after the COVID-19 pandemic. In the context of the proposed levelling-up agenda in England, this study adds more evidence to support the need for reinvestment in LA services as a means to improve population health outcomes and reduce health inequalities(40). Nutritional anaemias fell between 2005 and 2009, a period of a national health inequalities strategy in England which entailed sustained investment in LAs, the NHS, prevention and the welfare system (including tax credits, supplemental funds for pregnant women, and increases in the national pension (41). Policy-makers should draw on this past success and consider including health outcomes in the allocation criteria for levelling-up investments (42). Indeed, a national health inequalities strategy is once more needed to level up health across the country. However, individual-level research is needed to elucidate pathways between changes to LA service spending and nutritional anaemias, particularly with regards to different areas of LA service spending.

DATA AVAILABILITY STATEMENT

All data are available on the PLDR resource (pldr.org)

ETHICS STATEMENT

Patient Consent for Publication

Not required.

Ethics Approval

Ethical approval was granted for this study by Imperial College Research Ethics Committee (ICREC).

ACKNOWLEDGEMENTS

We are grateful to Christina Beatty and Steve Fothergill for sharing their data from The Uneven Impact of Welfare Reform project.

COMPETING INTERESTS

We have no competing interests to declare.

FUNDING

This study is funded by the National Institute for Health Research (NIHR) School for Public Health Research (SPHR), Grant Reference Number PD-SPH-2015. DTR is funded by the MRC on a Clinician Scientist Fellowship (MR/P008577/1). KD is supported by the National Institute for Health Research (NIHR) Applied Research Collaboration North West Coast (ARC NWC, NIHR200182).

Contributions

Study conception and design by RJ, EV, KM, AL. Data analysis was by RJ with input from RJ, EV, KM and AL. RJ wrote the first draft and all other authors made significant contributions to the final version.

For peer review only

Figure legends

Figure 1: Logic model of potential pathways through which changes to LA spending may affect nutritional anaemias

Figure 2: Rates of principal and total nutritional anaemia hospital admissions by year, stratified by age.

Figure 3: Scatter plots showing change in LA service spending in relation to changes in nutritional anaemia hospital admissions, prior to and following austerity respectively.

For peer review only

Table 1: Mean LA service expenditure in 2005, 2010, and 2018 and percentage change, stratified by socio-demographic variables.

	Total per capita LA spending 2005	Total per capita LA spending 2010	Total per capita LA spending 2018	Change 2005-2009	Change 2010-2018	Change 2005-2018
**Total**	845.4 (237.4)	861.6 (202.7)	690.9 (103.4)	+9.2%	-19.8%	-18.3%
**Region**						
North East	1007.0 (153.9)	1060.7 (166.8)	776.7 (78.6)	+11.5%	-26.8%	-22.9%
North West	882.7 (192.5)	943.9 (187.0)	741.7 (82.9)	+14.4%	-21.4%	-16.0%
Yorkshire & the Humber	773.1 (106.5)	839.5 (119.4)	661.7 (77.1)	+16.1%	-21.2%	-14.4%
East Midlands	705.0 (64.3)	776.7 (111.0)	620.0 (72.0)	+14.5%	-20.1%	-12.1%
West Midlands	770.2 (98.7)	768.7 (120.3)	655.8 (73.1)	+5.5%	-14.7%	-14.9%
London	1399.2 (272.7)	1263.1 (236.8)	870.0 (128.1)	+3.6%	-31.1%	-37.8%
South West	767.9 (64.2)	805.0 (74.5)	678.9 (52.0)	+10.0%	-15.7%	-11.6%
East of England	764.0 (48.9)	801.7 (49.9)	658.3 (42.1)	+11.1	-17.9%	-13.8%
South East	754.0 (81.3)	742.9 (75.1)	651.6 (61.9)	+4.7%	-12.3%	-13.6%
**Percentage male**						
<48.7%	825.5 (169.8)	851.5 (172.3)	687.6 (86.2)	+10.0%	-19.3%	-16.7%
48.7%-49.1%	855.1 (240.0)	859.5 (192.8)	658.8 (71.0)	+8.0%	-23.4%	-23.0%
49.1%-49.5%	811.2 (201.6)	834.0 (173.9)	674.7 (85.1)	+9.6%	-19.1%	-16.8%
>49.5%	907.9 (350.1)	906.6 (262.9)	725.5 (131.7)	+8.2%	-20.0%	-20.1%
**Percentage working age**						
<62.6%	761.2 (60.2)	786.8 (73.6)	659.6 (69.7)	+8.4%	-16.2%	-13.4%
62.6%-64.5%	782.8 (141.0)	771.7 (111.8)	687.7 (84.2)	+4.0%	-10.9%	-12.2%
64.5%-66.3%	786.7 (117.9)	838.9 (153.9)	700.7 (93.1)	+13.2%	-14.5%	-10.9%
>66.3%	985.5 (347.5)	995.5 (275.0)	812.2 (151.8)	+7.6%	-18.4%	-17.6%
**GDHI per capita[1]**						
<£15,892	884.8 (198.6)	956.1 (174.3)	740.0 (94.0)	+13.7%	-22.6%	-16.4%
£15,892-£18,346	782.9 (154.5)	795.8 (94.7)	659.9 (72.3)	+11.7%	-17.1%	-15.7%
£18,346-£21,422	824.6 (245.3)	816.3 (173.0)	656.8 (67.0)	+6.0%	-19.5%	-20.4%
>£21,422	893.9 (320.5)	891.9 (297.1)	708.5 (123.8)	+7.2%	-20.6%	-20.7%
**Unemployment rate**						
<3.8%	722.9 (48.7)	785.9 (75.8)	658.2 (70.2)	+19.9%	-16.3%	-9.0%
3-8%-5.1%	802.5 (117.0)	732.2 (58.6)	690.2 (92.8)	-2.1%	-5.7%	-14.0%
5.1%-6.8%	926.7 (177.4)	761.0 (83.8)	801.8 (141.5)	-12.3%	+5.4%	-13.5%
>6.8%	1352.3 (335.9)	972.6 (227.9)	842.4 (64.0)	-22.8%	-13.4%	-37.7%
**IMD**						
1 (most deprived)	1068.2 (326.0)	1084.2 (242.4)	782.5 (121.7)	+10.6%	-27.8%	-26.8%
2	883.2 (246.4)	914.2 (201.6)	713.0 (116.2)	+10.5%	-22.0%	-19.3%
3	798.0 (136.5)	809.0 (97.1)	664.0 (54.3)	+7.6%	-17.9%	-16.8%
4	746.4 (120.1)	769.5 (102.1)	647.3 (74.5)	+10.1%	-15.5%	-13.3%
5 (least deprived)	732.1 (89.6)	733.1 (82.6)	648.0 (62.0)	+6.8%	-11.6%	-11.5%
**Level of reductions to working age benefits per capita**						
1 (<£457)	726.6 (84.4)	731.3 (82.1)	643.6 (65.9)	+7.4%	-12.0%	-11.4%
2 (£457-£575)	805.8 (222.8)	819.3 (186.4)	675.5 (96.0)	+8.6%	-17.6%	-16.2%
3 (£575-£690)	868.8 (261.0)	885.8 (197.0)	694.2 (107.6)	+9.2%	-21.6%	-20.1%
4 (>£690)	985.3 (262.5)	1013.4 (208.1)	751.7 (108.8)	+11.3%	-25.8%	-23.7%

[1] GDHI is Gross Disposable Household Income per capita per year, an area-level measure of individuals' available money for spending or saving following payment of taxes and receipt of benefits

[2] Index of Multiple Deprivation, quintiles based on relative ranking of LAs

For peer review only

Table 2: Mean rate of principal and total nutritional anaemia hospital admissions per 100,000 LA population in 2018, stratified by socio-demographic variables.

	Mean rate of nutritional anaemia hospital admissions per 100,000 LA population in 2018 Mean (SE in brackets)							
	All ages		0-14		15-64		65+	
	Principal nutritional anaemia hospital admissions	Total nutritional anaemia hospital admissions	Principal nutritional anaemia hospital admissions	Total nutritional anaemia hospital admissions	Principal nutritional anaemia hospital admissions	Total nutritional anaemia hospital admissions	Principal nutritional anaemia hospital admissions	Total nutritional anaemia hospital admissions
**Total**	236.4 (95.8)	632.4 (213.7)	11.1 (16.9)	56.8 (41.1)	154.1 (65.0)	363.6 (145.8)	717.1 (278.3)	2101.8 (765.5)
**Region**								
North East	306.7 (106.1)	938.4 (268.3)	13.5 (6.9)	67.7 (26.2)	208.6 (79.3)	553.2 (210.4)	898.1 (329.1)	2997.9 (851.1)
North West	284.5 (88.6)	836.4 (200.5)	22.4 (35.4)	83.9 (62.5)	189.6 (72.8)	492.8 (172.2)	854.7 (308.1)	2688.0 (753.8)
Yorkshire & the Humber	278.3 (73.7)	698.0 (149.8)	7.9 (9.9)	55.2 (36.5)	181.9 (55.4)	404.8 (127.4)	804.8 (214.0)	2154.9 (374.2)
East Midlands	206.7 (86.1)	531.5 (179.1)	6.7 (8.8)	35.7 (29.0)	136.7 (61.1)	299.9 (113.1)	596.0 (261.4)	1677.8 (557.2)
West Midlands	211.0 (70.6)	584.5 (155.0)	11.1 (10.7)	61.9 (42.6)	146.5 (52.8)	347.9 (113.2)	585.8 (199.5)	1788.7 (503.3)
London	183.2 (62.5)	644.2 (140.2)	13.7 (9.1)	85.4 (29.3)	137.5 (49.4)	415.1 (109.8)	719.9 (209.0)	2956.2 (763.2)
South West	271.2 (119.0)	628.3 (204.4)	6.8 (8.4)	43.2 (28.7)	164.0 (69.0)	332.6 (108.1)	730.1 (313.1)	1825.1 (592.2)
East of England	255.2 (115.1)	620.2 (217.7)	9.8 (14.3)	47.7 (32.1)	157.2 (72.1)	354.6 (127.1)	786.0 (304.0)	1985.4 (582.0)
South East	205.8 (71.6)	515.4 (150.5)	9.5 (14.2)	48.1 (34.5)	127.7 (48.1)	276.6 (90.3)	651.3 (232.8)	1758.6 (571.3)
**Percentage male**								
<48.7%	238.0 (99.8)	614.5 (216.4)	9.7 (13.4)	48.9 (32.3)	144.9 (60.9)	332.2 (127.0)	662.1 (249.0)	1834.3 (581.6)
48.7%-49.1%	250.4 (100.3)	646.5 (206.5)	8.1 (9.6)	47.3 (31.1)	153.4 (61.9)	351.9 (128.1)	725.0 (298.2)	1981.2 (625.1)
49.1%-49.5%	240.4 (104.9)	633.1 (248.4)	12.3 (16.5)	57.9 (43.3)	159.7 (76.5)	364.8 (170.2)	725.3 (305.2)	2082.4 (844.3)
>49.5%	223.8 (81.3)	635.5 (186.0)	12.9 (22.0)	66.9 (47.5)	156.0 (59.4)	390.7 (142.7)	742.7 (260.1)	2372.5 (805.6)
**Percentage working age**								
<62.6%	241.5 (103.4)	624.5 (223.7)	9.2 (14.3)	47.8 (37.5)	148.4 (65.3)	337.0 (139.1)	662.6 (275.8)	1826.9 (616.5)
62.6%-64.5%	256.9 (85.8)	681.2 (223.6)	12.0 (11.2)	60.9 (38.5)	174.3 (67.9)	411.4 (167.4)	801.5 (259.2)	2278.9 (690.9)
64.5%-66.3%	223.0 (85.1)	591.4 (175.7)	17.8 (37.8)	75.1 (59.1)	163.7 (66.6)	381.9 (137.9)	763.8 (298.2)	2230.1 (704.6)
>66.3%	191.5 (71.8)	609.0 (168.0)	12.6 (9.5)	72.8 (35.2)	136.1 (49.1)	375.1 (115.6)	757.2 (266.2)	2799.7 (907.6)
**GDHI**[1]								

<£15,892	301.8 (102.0)	817.8 (244.4)	19.4 (30.4)	86.8 (59.3)	208.7 (69.3)	507.4 (180.5)	889.2 (281.3)	2628.6 (746.9)
£15,892-£18,346	241.0 (103.8)	649.8 (221.2)	10.1 (9.4)	52.1 (31.5)	156.2 (67.1)	368.4 (135.5)	704.1 (288.3)	2066.1 (692.3)
£18,346-£21,422	235.7 (90.7)	598.8 (172.8)	9.3 (12.9)	45.7 (30.2)	151.3 (57.2)	331.2 (103.6)	692.8 (295.8)	1911.2 (630.6)
>£21,422	200.6 (70.5)	549.8 (155.5)	9.1 (12.6)	53.2 (36.1)	126.9 (48.3)	310.9 (109.1)	656.3 (219.9)	1995.6 (804.9)
**Unemployment rate**								
<3.8%	230.6 (96.5)	579.0 (191.7)	8.3 (13.1)	44.1 (31.3)	140.3 (61.3)	310.7 (111.1)	673.5 (278.9)	1793.7 (579.8)
3-8%-5.1%	242.8 (92.5)	656.6 (203.7)	12.1 (21.6)	63.4 (47.2)	164.3 (60.5)	387.9 (131.7)	750.3 (276.9)	2226.3 (670.2)
5.1%-6.8%	237.5 (102.4)	752.4 (242.7)	18.9 (16.1)	84.0 (39.0)	175.0 (76.7)	482.5 (179.4)	786.8 (256.0)	2911.8 (887.9)
>6.8%	279.4 (84.1)	856.6 (256.7)	19.7 (8.8)	117.9 (28.0)	221.2 (60.6)	602.4 (184.1)	867.9 (297.3)	2873.1 (706.2)
**IMD[1]**								
1 (most deprived)	254.9 (99.1)	764.9 (230.5)	20.2 (28.0)	90.5 (51.6)	186.5 (75.1)	493.9 (173.0)	817.3 (267.4)	2781.7 (775.2)
2	246.6 (113.1)	670.4 (234.7)	11.1 (9.3)	61.4 (36.6)	165.0 (66.7)	390.3 (138.7)	742.9 (321.4)	2252.2 (847.3)
3	241.1 (89.3)	622.9 (177.6)	11.0 (13.5)	51.9 (32.2)	156.2 (57.2)	350.2 (103.7)	710.8 (275.3)	1993.3 (602.7)
4	238.8 (94.4)	595.1 (180.2)	7.9 (13.6)	41.9 (30.8)	142.8 (59.6)	314.4 (106.7)	692.2 (277.3)	1829.7 (541.6)
5 (least deprived)	202.3 (73.3)	512.3 (153.8)	5.5 (8.9)	38.5 (28.1)	120.9 (45.6)	271.2 (82.4)	625.1 (214.7)	1665.2 (467.5)
**Level of reductions to working age benefits per capita**								
1 (<£457)	206.2 (75.7)	521.9 (150.1)	5.3 (8.5)	39.4 (30.0)	123.0 (46.8)	275.7 (82.5)	630.4 (231.7)	1679.5 (484.8)
2 (£457-£575)	238.8 (100.4)	610.6 (196.9)	11.5 (16.1)	50.2 (33.5)	145.3 (60.7)	327.4 (111.3)	703.0 (284.4)	1962.2 (717.5)
3 (£575-£690)	239.5 (105.0)	639.8 (201.6)	9.3 (8.6)	51.6 (30.9)	158.2 (62.7)	366.4 (115.2)	744.6 (305.4)	2208.3 (748.8)
4 (>£690)	263.3 (92.7)	759.8 (233.6)	18.9 (26.3)	88.0 (51.4)	192.4 (70.0)	489.5 (172.6)	795.5 (263.7)	2571.1 (801.0)

[1] GDHI is Gross Disposable Household Income per capita per year, an area-level measure of individuals' available money for spending or saving following payment of taxes and receipt of benefits

[2] Index of Multiple Deprivation, quintiles based on relative ranking of LAs

For peer review only

Table 3: Association between LA service spending and total nutritional anaemia hospital admissions, shown as the Incident Rate Ratio and stratified by age (95% Confidence Intervals in brackets)

	Incidence Rate Ratio for total nutritional anaemia hospital admissions by LA with an £100 increase in total LA service spending			
	All ages	0-14	15-64	65+
Full Sample[1]	0.981 (0.964, 0.999) p=0.038	0.974 (0.904, 1.050) p=0.488	0.978 (0.959, 0.998) p=0.033	0.979 (0.960, 0.997) p=0.024
IMD[2]				
1 (most deprived)	0.974 (0.951, 0.998) p=0.032	0.943 (0.834, 1.066) p=0.348	0.971 (0.943, 0.999) p=0.043	0.974 (0.950, 0.999) p=0.038
2	0.991 (0.949, 1.035) p=0.678	1.030 (0.980, 1.082) p=0.251	0.999 (0.957, 1.043) p=0.960	0.982 (0.936, 1.030) p=0.452
3	1.016 (0.976, 1.058) p=0.431	1.032 (0.938, 1.135) p=0.524	1.019 (0.968, 1.073) p=0.472	1.006 (0.966, 1.047) p=0.779
4	0.883 (0.831, 0.939) p<0.001	0.929 (0.790, 1.092) p=0.371	0.851 (0.792, 0.915) p<0.001	0.891 (0.838, 0.946) p<0.001
5 (least deprived)	1.011 (0.955, 1.070) p=0.712	1.014 (0.860, 1.195) p=0.870	0.989 (0.922, 1.063) p=0.768	1.018 (0.966, 1.072) p=0.505
Level of reductions to working age benefits per capita				
1 (lowest reductions)	1.011 (0.961, 1.063) p=0.680	1.013 (0.877, 1.170) p=0.864	1.000 (0.946, 1.056) p=0.985	1.013 (0.962, 1.067) p=0.616
2	0.963 (0.935, 0.991) p=0.010	0.955 (0.889, 1.026) p=0.208	0.954 (0.920, 0.989) p=0.010	0.963 (0.935, 0.992) p=0.012
3	0.992 (0.952, 1.032) p=0.671	1.029 (0.928, 1.141) p=0.587	0.995 (0.955, 1.036) p=0.803	0.985 (0.942, 1.030) p=0.500
4 (greatest reductions)	0.975 (0.949, 1.003) p=0.077	0.935 (0.824, 1.061) p=0.294	0.973 (0.941, 1.006) p=0.104	0.973 (0.947, 0.999) p=0.043

[1] Adjusted by percentage working age, percentage male, GDHI and unemployment rate.
[2] Index of Multiple Deprivation, quintiles based on relative ranking of LAs.

For peer review only

REFERENCES

1. World Health Organisation. Anaemia 2021 [Available from: https://www.who.int/health-topics/anaemia#tab=tab_2.
2. von Haehling S, Jankowska EA, van Veldhuisen DJ, Ponikowski P, Anker SD. Iron deficiency and cardiovascular disease. Nature Reviews Cardiology. 2015;12(11):659-69.
3. Darnton-Hill I, Webb P, Harvey PWJ, Hunt JM, Dalmiya N, Chopra M, et al. Micronutrient deficiencies and gender: social and economic costs. The American Journal of Clinical Nutrition. 2005;81(5):1198S-205S.
4. Lim SS, Vos T, Flaxman AD, Danaei G, Shibuya K, Adair-Rohani H, et al. A comparative risk assessment of burden of disease and injury attributable to 67 risk factors and risk factor clusters in 21 regions, 1990-2010: a systematic analysis for the Global Burden of Disease Study 2010. Lancet. 2012;380(9859):2224-60.
5. NHS Digital. Hospital Admitted Patient Care Activity 2020 [Available from: https://digital.nhs.uk/data-and-information/publications/statistical/hospital-admitted-patient-care-activity.
6. House of Commons Environmental Audit Committee. Sustainable Development Goals in the UK follow up: hunger, malnutrition and food insecurity in the UK. London: House of Commons; 2019.
7. HM Treasury. Budget 2010. 2010.
8. HM Treasury. Chancellor announces reforms to the welfare system 2010 [Available from: https://www.gov.uk/government/news/chancellor-announces-reforms-to-the-welfare-system.
9. Smith NA, Phillips D, Simpson P, Eiser D, Trickey M. A time of revolution? British local government finance in the 2010s. Institute for Fiscal Studies; 2016.
10. Centre for Cities. Cities Outlook 2019: A Decade of Austerity 2019.
11. Ministry of Housing Communities & Local Government. General Fund Revenue Account Outturn 2019-20 Specific guidance notes for completing the REVENUE OUTTURN (RO) suite of forms. 2020.
12. Bambra C, Gibson M, Sowden A, Wright K, Whitehead M, Petticrew M. Tackling the wider social determinants of health and health inequalities: evidence from systematic reviews. Journal of Epidemiology and Community Health. 2010;64(4):284.
13. Marmot M, Allen, J, Boyce, T, et al. Health Equity in England: The Marmot Review 10 Years On. 2020.
14. Alexiou A, Fahy K, Mason K, Bennett D, Brown H, Bambra C, et al. Local government funding and life expectancy in England: a longitudinal ecological study. The Lancet Public Health. 2021;6:e641-47.
15. Jenkins RH, Aliabadi S, Vamos EP, et al. The relationship between austerity and food insecurity in the UK: A systematic review. EClinicalMedicine. 2021;33:100781.
16. Penny JS, J;. Surviving Austerity. Local voices and local action in England's poorest neighbourhoods. New Economics Foundation; 2013.
17. Humphries RH, P; Charles, A; Thorlby, R; Holder, H. Social care for older people: Home truths. The Kings Fund 2016. Report No.: 978 1 909029 66 8.
18. Office for National Statistics. Family spending in the UK: April 2018 to March 2019 2020 [Available from: https://www.ons.gov.uk/peoplepopulationandcommunity/personalandhouseholdfinances/expenditure/bulletins/familyspendingintheuk/april2018tomarch2019
19. Pollack CE, Griffin BA, Lynch J. Housing Affordability and Health Among Homeowners and Renters. American Journal of Preventive Medicine. 2010;39(6):515-21.
20. https://pldr.org/ PBLR. [
21. Innes D, Tetlow G. Delivering Fiscal Squeeze by Cutting Local Government Spending. Fiscal Studies. 2015;36(3):303-25.
22. Office for National Statistics. CPIH ANNUAL RATE 00: ALL ITEMS 2015=100 2021 [Available from: https://www.ons.gov.uk/economy/inflationandpriceindices/timeseries/l55o/mm23.

For peer review only

23. NHS Digital. Hospital Episode Statistics (HES) 2021 [Available from: https://digital.nhs.uk/data-and-information/data-tools-and-services/data-services/hospital-episode-statistics.
24. U.S. Federal Government's Department of Health and Human Services. ICD-10-CM Official Guidelines for Coding and Reporting. 2020.
25. Office for National Statistics. Lower layer Super Output Area population estimates 2020 [Available from: https://www.ons.gov.uk/peoplepopulationandcommunity/populationandmigration/populationestimates/datasets/lowersuperoutputareamidyearpopulationestimates.
26. Office for National Statistics. Regional gross disposable household income QMI 2018 [Available from: https://www.ons.gov.uk/economy/regionalaccounts/grossdisposablehouseholdincome/methodologies/regionalgrossdisposablehouseholdincomeqmi.
27. Office for National Statistics. Model-Based Estimates of ILO Unemployment for LAD/UAs in Great Britain: Guide for Users. 2006.
28. Ministry of Housing Communities & Local Government. English indices of deprivation 2015 2015 [Available from: https://www.gov.uk/government/statistics/english-indices-of-deprivation-2015].
29. Beatty C, Fothergill, S. The uneven impact of welfare reform: the financial losses to places and people. Sheffield Hallam University Centre for Regional Economic and Social Research; 2016.
30. Cherrie M, Curtis S, Baranyi G, Cunningham N, Dibben C, Bambra C, et al. A data linkage study of the effects of the Great Recession and austerity on antidepressant prescription usage. European Journal of Public Health. 2021;31(2):297-303.
31. Wooldridge JM. Econometric analysis of cross section and panel data: MIT press; 2010.
32. Wooldridge JM. Distribution-free estimation of some nonlinear panel data models. Journal of Econometrics. 1999;90(1):77-97.
33. Bertrand M, Duflo E, Mullainathan S. How Much Should We Trust Differences-In-Differences Estimates? The Quarterly Journal of Economics. 2004;119(1):249-75.
34. Elia M. The cost of malnutrition in England and potential cost savings from nutritional interventions (short version): A report on the cost of disease-related malnutrition in England and a budget impact analysis of implementing the NICE clinical guidelines/quality standard on nutritional support in adults. BAPEN; 2015.
35. Public Health England. National Diet and Nutrition Survey. Years 1 to 9 of the Rolling Programme (2008/2009 – 2016/2017): Time trend and income analyses. 2019.
36. Public Health England. National Diet and Nutrition Survey. Rolling programme Years 9 to 11 (2016/2017 to 2018/2019). 2020.
37. Russell CA, Elia M. NUTRITION SCREENING SURVEYS IN HOSPITALS IN THE UK, 2007-2011. BAPEN 2014.
38. Watkins J, Wulaningsih W, Da Zhou C, Marshall DC, Sylianteng GDC, Dela Rosa PG, et al. Effects of health and social care spending constraints on mortality in England: a time trend analysis. BMJ Open. 2017;7(11):e017722.
39. McLean G, Gunn J, Wyke S, Guthrie B, Watt GC, Blane DN, et al. The influence of socioeconomic deprivation on multimorbidity at different ages: a cross-sectional study. British Journal of General Practice. 2014;64(624):e440-e7.
40. The Rt Hon Boris Johnson MP. The Prime Minister's Levelling Up speech: 15 July 2021 2021 [Available from: https://www.gov.uk/government/speeches/the-prime-ministers-levelling-up-speech-15-july-2021.
41. Robinson T, Brown H, Norman PD, Fraser LK, Barr B, Bambra C. The impact of New Labour's English health inequalities strategy on geographical inequalities in infant mortality: a time-trend analysis. Journal of Epidemiology and Community Health. 2019;73(6):564-8.
42. Dixon J, Everest G. Briefing: The government's levelling up agenda The Health Foundation; 2021 [Available from: https://reader.health.org.uk/governments-levelling-up-agenda.

For peer review only

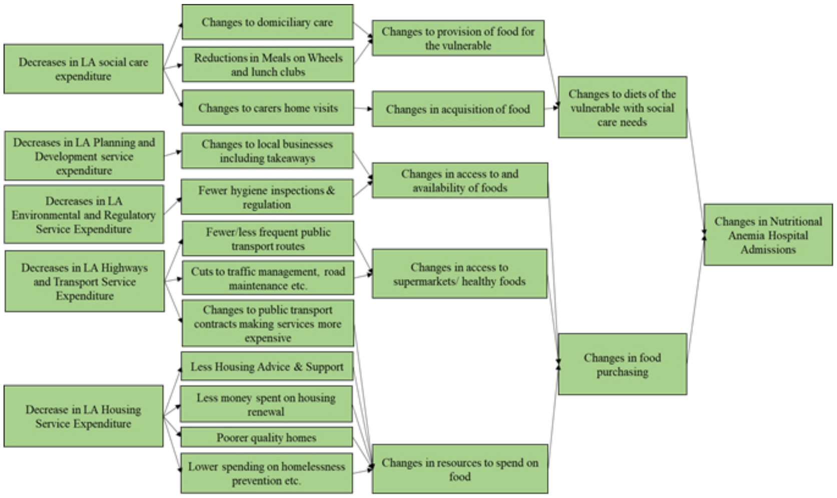

Figure 1: Logic model of potential pathways through which changes to LA spending may affect nutritional anaemias

216x121mm (96 x 96 DPI)

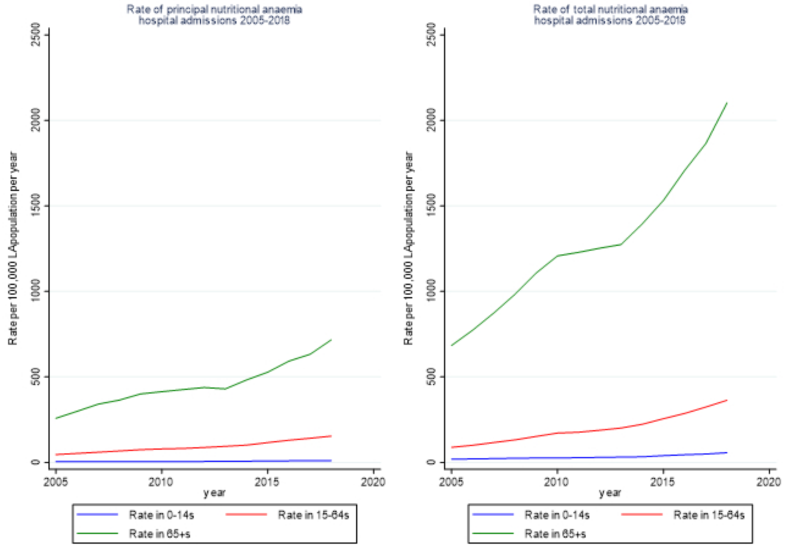

Figure 2: Rates of principal and total nutritional anaemia hospital admissions by year, stratified by age.

216x128mm (96 x 96 DPI)

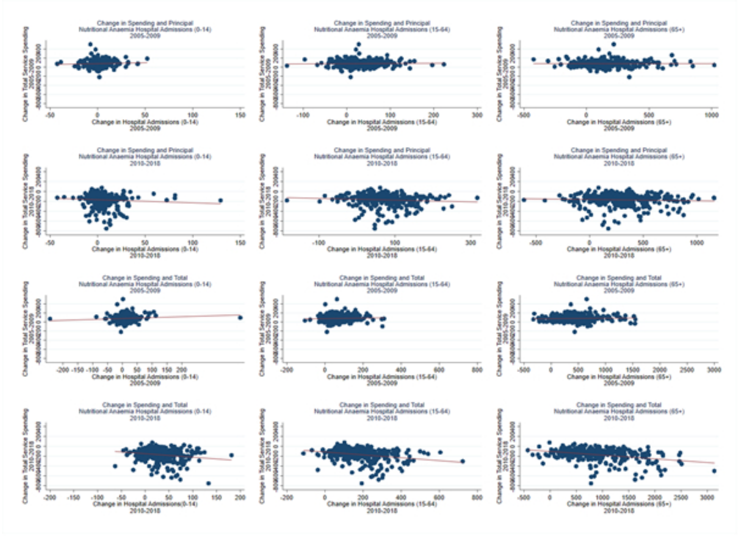

Figure 3: Scatter plots showing change in LA service spending in relation to changes in nutritional anaemia hospital admissions, prior to and following austerity respectively.

216x121mm (96 x 96 DPI)

Appendix 1: ICD-10 codes for conditions included in this study

**ICD-10 code**	**Diagnosis**
Principal and total nutritional anaemia hospital admissions:	
D50	Iron deficiency anaemia
D51	Vitamin B12 deficiency anaemia
D52	Folate deficiency anaemia
D53	Other nutritional anaemias
Excluding admissions alongside a principal or secondary diagnosis of:	
D50.0	Iron deficiency anaemia secondary to blood loss
C00-C96	Malignancies
F50	Eating disorders
R13	Difficulty swallowing
K91.2	Postoperative malabsorption
R64	Cachexia
E70-90	Metabolic disorders
K50.90, K51.90	GI disease including Crohn's disease and ulcerative colitis
K63.2	Fistula
Z99.2, N17-19	Kidney disease and dialysis
K70-77	Liver disease
E08-E11, O24.4	Diabetes mellitus (due to underlying condition, drug or chemical induced, type I, type II, gestational)

For peer review only

Appendix 2: Changes in mean total LA spending over time

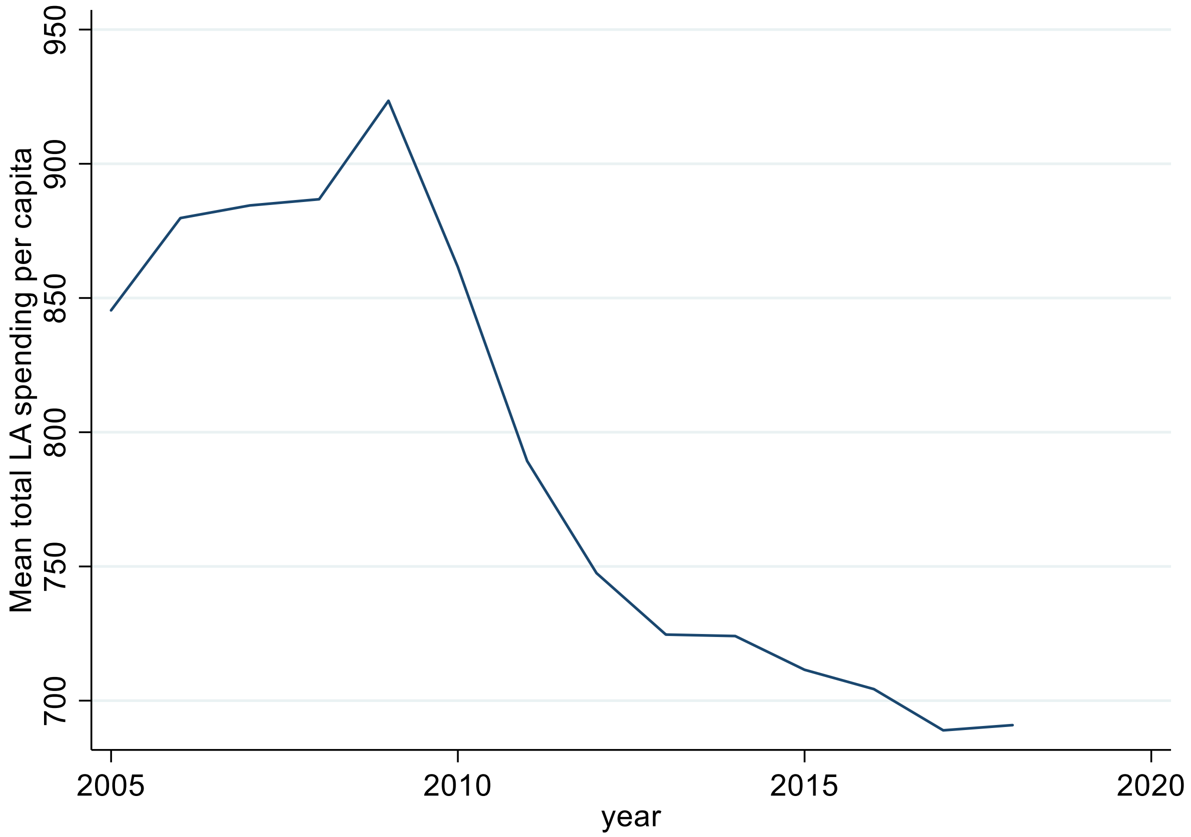

For peer review only

Appendix 3: Impact of LA service spending on total nutritional anaemia hospital admissions, shown as the Incident Rate Ratio and stratified by gender (95% Confidence Intervals in brackets)

	Incidence Rate Ratio for total nutritional anaemia hospital admissions by LA with an £100 increase in total LA service spending		
	All Ages	Male	Female
Full Sample[1]	0.981 (0.964, 0.999) p=0.038	0.975 (0.957, 0.993) p=0.007	0.992 (0.974, 1.010) p=0.358
IMD[2]			
1 (most deprived)	0.974 (0.951, 0.998) p=0.032	0.968 (0.944, 0.993) p=0.012	0.980 (0.956, 1.005) p=0.118
2	0.991 (0.949, 1.035) p=0.678	0.988 (0.947, 1.030) p=0.563	1.005 (0.962, 1.050) p=0.818
3	1.016 (0.976, 1.058) p=0.431	1.009 (0.962, 1.058) p=0.707	1.032 (0.989, 1.076) p=0.151
4	0.883 (0.831, 0.939) p<0.001	0.872 (0.817, 0.932) p<0.001	0.902 (0.851, 0.956) p<0.001
5 (least deprived)	1.011 (0.955, 1.070) p=0.712	1.001 (0.936, 1.071) p=0.967	1.029 (0.967, 1.094) p=0.367
Level of reductions to working age benefits per capita			
1 (lowest reductions)	1.011 (0.961, 1.063) p=0.680	1.013 (0.962, 1.067) p=0.623	1.020 (0.962, 1.083) p=0.506
2	0.963 (0.935, 0.991) p=0.010	0.955 (0.926, 0.985) p=0.004	0.981 (0.951, 1.011) p=0.210
3	0.992 (0.952, 1.032) p=0.671	0.983 (0.944, 1.024) p=0.419	0.999 (0.960, 1.040) p=0.960
4 (greatest reductions)	0.975 (0.949, 1.003) p=0.077	0.970 (0.942, 1.000) p=0.048	0.986 (0.957, 1.015) p=0.330

[1] Adjusted by percentage working age, percentage male, GDHI and unemployment rate.
[2] Index of Multiple Deprivation, quintiles based on relative ranking of LAs

For peer review only

Appendix 4: Association between LA service spending and principal nutritional anaemia hospital admissions, shown as the Incident Rate Ratio and stratified by age (95% Confidence Intervals in brackets).

	Incidence Rate Ratio for principal nutritional anaemia hospital admissions by LA with an £100 increase in total LA service spending			
	All Ages	0-14	15-64	65+
Full sample[1]	0.987 (0.963, 1.011) p=0.293	0.996 (0.945, 1.051) p=0.896	0.986 (0.957, 1.015) p=0.332	0.983 (0.957, 1.009) p=0.190
IMD[2]				
1 (most deprived)	0.985 (0.950, 1.022) p=0.435	0.969 (0.899, 1.043) p=0.400	0.989 (0.945, 1.035) p=0.634	0.978 (0.941, 1.016) p=0.247
2	0.988 (0.942, 1.036) p=0.611	0.984 (0.866, 1.119) p=0.806	1.000 (0.950, 1.053) p=0.999	0.975 (0.928, 1.024) p=0.311
3	1.031 (0.973, 1.092) p=0.308	1.086 (0.895, 1.319) p=0.402	1.051 (0.989, 1.116) p=0.107	1.002 (0.929, 1.082) p=0.951
4	0.838 (0.759, 0.925) p<0.001	1.255 (1.009, 1.560) p=0.041	0.798 (0.715, 0.892) p<0.001	0.853 (0.769, 0.946) p=0.003
5 (least deprived)	1.040 (0.957, 1.130) p=0.357	1.135 (0.943, 1.365) p=0.180	1.013 (0.929, 1.104) p=0.778	1.053 (0.968, 1.146) p=0.226
Level of reductions to working age benefits per capita				
1 (lowest reductions)	1.041 (0.972, 1.115) p=0.252	1.205 (1.001, 1.451) p=0.049	1.019 (0.949, 1.095) p=0.601	1.052 (0.977, 1.132) p=0.180
2	0.967 (0.927, 1.009) p=0.125	1.002 (0.835, 1.203) p=0.983	0.959 (0.910, 1.010) p=0.113	0.968 (0.927, 1.012) p=0.148
3	1.000 (0.949, 1.054) p=0.995	1.030 (0.919, 1.153) p=0.616	1.016 (0.965, 1.070) p=0.548	0.978 (0.920, 1.040) p=0.479
4 (greatest reductions)	0.980 (0.947, 1.014) p=0.242	0.975 (0.916, 1.038) p=0.430	0.979 (0.937, 1.022) p=0.333	0.975 (0.943, 1.008) p=0.140

[1] Adjusted by percentage working age, percentage male, GDHI and unemployment rate.

[2] Index of Multiple Deprivation, quintiles based on relative ranking of LAs

For peer review only

Appendix 5: Rates of principal and total nutritional anaemia hospital admissions over time, excluding those alongside other conditions which may cause nutritional deficiencies, stratified by age (2005-2018)

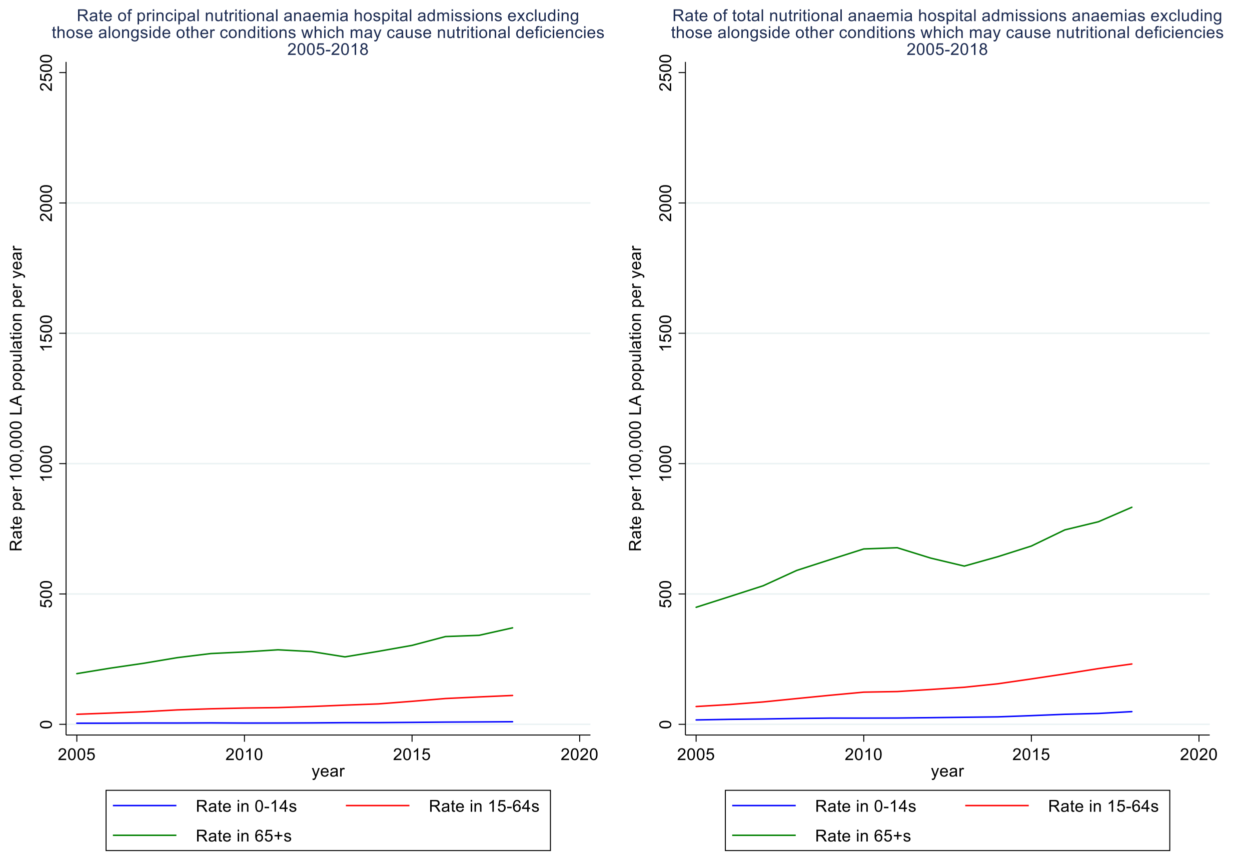

For peer review only

Appendix 6: Association between LA service spending and total nutritional anaemia hospital admissions excluding those alongside a principal or secondary diagnosis of another condition which may cause a nutritional deficiency, shown as the Incident Rate Ratio and stratified by age (95% Confidence Intervals in brackets)

	Incidence Rate Ratio for total nutritional anaemia hospital admissions by LA with an £100 increase in total LA service spending			
	All Ages	0-14	15-64	65+
Full sample[1]	0.991 (0.974, 1.009) p=0.312	0.967 (0.894, 1.047) p=0.408	0.989 (0.969, 1.009) p=0.279	0.998 (0.980, 1.017) p=0.829
IMD[2]				
1 (most deprived)	0.985 (0.962, 1.009) p=0.208	0.933 (0.823, 1.058) p=0.279	0.981 (0.952, 1.011) p=0.207	0.994 (0.969, 1.020) p=0.658
2	1.001 (0.961, 1.044) p=0.950	1.047 (0.989, 1.109) p=0.118	1.009 (0.967, 1.052) p=0.692	1.002 (0.955, 1.050) p=0.945
3	1.034 (0.998, 1.072) p=0.067	1.025 (0.934, 1.125) p=0.599	1.041 (0.991, 0.093) p=0.110	1.029 (0.992, 1.066) p=0.128
4	0.876 (0.818, 0.938) p<0.001	0.931 (0.789, 1.098) p=0.396	0.850 (0.786, 0.920) p<0.001	0.888 (0.828, 0.953) p=0.001
5 (least deprived)	1.030 (0.968, 1.095) p=0.357	0.977 (0.818, 1.167) p=0.799	1.024 (0.959, 1.094) p=0.472	1.038 (0.976, 1.104) p=0.237
Level of reductions to working age benefits per capita				
1 (lowest reductions)	1.026 (0.968, 1.087) p=0.390	0.979 (0.834, 1.149) p=0.792	1.031 (0.976, 1.088) p=0.279	1.027 (0.963, 1.096) p=0.412
2	0.976 (0.947, 1.005) p=0.104	0.973 (0.906, 1.045) p=0.446	0.960 (0.924, 0.997) p=0.035	0.993 (0.964, 1.023) p=0.656
3	1.004 (0.964, 1.047) p=0.839	1.030 (0.919, 1.154) p=0.616	1.012 (0.970, 1.055) p=0.590	1.002 (0.956, 1.050) p=0.946
4 (greatest reductions)	0.981 (0.956, 1.007) p=0.150	0.925 (0.813, 1.052) p=0.234	0.978 (0.946, 1.012) p=0.198	0.988 (0.963, 1.013) p=0.326

[1] Adjusted by percentage working age, percentage male, GDHI and unemployment rate.

[2] Index of Multiple Deprivation, quintiles based on relative ranking of LAs

For peer review only

Appendix 7: Association between LA service spending excluding social care spending and total nutritional anaemia hospital admissions, shown as the Incident Rate Ratio and stratified by age (95% Confidence Intervals in brackets)

	Incidence Rate Ratio for total nutritional anaemia hospital admissions by LA with an £100 increase in total LA service spending			
	All Ages	0-14	15-64	65+
Full Sample[1]	0.960 (0.939, 0.982) p<0.001	0.960 (0.869, 1.061) p=0.425	0.958 (0.934, 0.983) p=0.001	0.958 (0.936, 0.980) p<0.001
IMD[2]				
1 (most deprived)	0.957 (0.927, 0.987) p=0.006	0.903 (0.769, 1.060) p=0.213	0.950 (0.916, 0.986) p=0.007	0.962 (0.931, 0.995) p=0.025
2	0.976 (0.935, 1.020) p=0.278	1.074 (1.020, 1.131) p=0.006	0.989 (0.944, 1.037) p=0.652	0.962 (0.919, 1.008) p=0.104
3	0.988 (0.934, 1.045) p=0.670	1.087 (0.973, 1.214) p=0.141	0.990 (0.927, 1.056) p=0.750	0.973 (0.919, 1.030) p=0.349
4	0.847 (0.796, 0.901) p<0.001	0.959 (0.803, 1.146) p=0.645	0.822 (0.760, 0.888) p<0.001	0.849 (0.799, 0.901) p<0.001
5 (least deprived)	1.003 (0.931, 1.081) p=0.934	1.031 (0.889, 1.195) p=0.687	1.005 (0.924, 1.092) p=0.916	0.997 (0.933, 1.065) p=0.934
Level of reductions to working age benefits per capita				
1 (lowest reductions)	0.982 (0.914, 1.055) p=0.612	0.997 (0.845, 1.177) p=0.974	0.984 (0.910, 1.065) p=0.695	0.975 (0.912, 1.042) p=0.457
2	0.940 (0.903, 0.979) p=0.003	0.957 (0.874, 1.047) p=0.339	0.936 (0.891, 0.982) p=0.007	0.937 (0.900, 0.975) p=0.001
3	0.961 (0.922, 1.002) p=0.062	1.025 (0.912, 1.152) p=0.685	0.966 (0.924, 1.009) p=0.123	0.951 (0.909, 0.994) p=0.027
4 (greatest reductions)	0.963 (0.930, 0.997) p=0.031	0.911 (0.774, 1.072) p=0.261	0.959 (0.921, 0.999) p=0.043	0.965 (0.932, 1.000) p=0.047

[1] Adjusted by percentage working age, percentage male, GDHI and unemployment rate.

[2] Index of Multiple Deprivation, quintiles based on relative ranking of LAs

For peer review only

STROBE Statement—checklist of items that should be included in reports of observational studies

	Item No	Recommendation	Page No
**Title and abstract**	1	(*a*) Indicate the study's design with a commonly used term in the title or the abstract	1
		(*b*) Provide in the abstract an informative and balanced summary of what was done and what was found	1
**Introduction**			
Background/rationale	2	Explain the scientific background and rationale for the investigation being reported	2,3
Objectives	3	State specific objectives, including any prespecified hypotheses	3
**Methods**			
Study design	4	Present key elements of study design early in the paper	3
Setting	5	Describe the setting, locations, and relevant dates, including periods of recruitment, exposure, follow-up, and data collection	3,4
Participants	6	(*a*) *Cohort study*—Give the eligibility criteria, and the sources and methods of selection of participants. Describe methods of follow-up<br>*Case-control study*—Give the eligibility criteria, and the sources and methods of case ascertainment and control selection. Give the rationale for the choice of cases and controls<br>*Cross-sectional study*—Give the eligibility criteria, and the sources and methods of selection of participants	4
		(*b*) *Cohort study*—For matched studies, give matching criteria and number of exposed and unexposed<br>*Case-control study*—For matched studies, give matching criteria and the number of controls per case	
Variables	7	Clearly define all outcomes, exposures, predictors, potential confounders, and effect modifiers. Give diagnostic criteria, if applicable	5
Data sources/ measurement	8*	For each variable of interest, give sources of data and details of methods of assessment (measurement). Describe comparability of assessment methods if there is more than one group	5
Bias	9	Describe any efforts to address potential sources of bias	5,6
Study size	10	Explain how the study size was arrived at	6
Quantitative variables	11	Explain how quantitative variables were handled in the analyses. If applicable, describe which groupings were chosen and why	6
Statistical methods	12	(*a*) Describe all statistical methods, including those used to control for confounding	6,7
		(*b*) Describe any methods used to examine subgroups and interactions	7
		(*c*) Explain how missing data were addressed	7
		(*d*) *Cohort study*—If applicable, explain how loss to follow-up was addressed<br>*Case-control study*—If applicable, explain how matching of cases and controls was addressed<br>*Cross-sectional study*—If applicable, describe analytical methods taking account of sampling strategy	7
		(*e*) Describe any sensitivity analyses	

Continued on next page

For peer review only

**Results**			
Participants	13*	(a) Report numbers of individuals at each stage of study—eg numbers potentially eligible, examined for eligibility, confirmed eligible, included in the study, completing follow-up, and analysed	7
		(b) Give reasons for non-participation at each stage	7
		(c) Consider use of a flow diagram	7
Descriptive data	14*	(a) Give characteristics of study participants (eg demographic, clinical, social) and information on exposures and potential confounders	Table 1
		(b) Indicate number of participants with missing data for each variable of interest	Table 1
		(c) *Cohort study*—Summarise follow-up time (eg, average and total amount)	
Outcome data	15*	*Cohort study*—Report numbers of outcome events or summary measures over time	Table 1
		Case-control study—Report numbers in each exposure category, or summary measures of exposure	
		Cross-sectional study—Report numbers of outcome events or summary measures	
Main results	16	(*a*) Give unadjusted estimates and, if applicable, confounder-adjusted estimates and their precision (eg, 95% confidence interval). Make clear which confounders were adjusted for and why they were included	8
		(*b*) Report category boundaries when continuous variables were categorized	8
		(*c*) If relevant, consider translating estimates of relative risk into absolute risk for a meaningful time period	8
Other analyses	17	Report other analyses done—eg analyses of subgroups and interactions, and sensitivity analyses	8,9
**Discussion**			
Key results	18	Summarise key results with reference to study objectives	9
Limitations	19	Discuss limitations of the study, taking into account sources of potential bias or imprecision. Discuss both direction and magnitude of any potential bias	10
Interpretation	20	Give a cautious overall interpretation of results considering objectives, limitations, multiplicity of analyses, results from similar studies, and other relevant evidence	10,11
Generalisability	21	Discuss the generalisability (external validity) of the study results	11
**Other information**			
Funding	22	Give the source of funding and the role of the funders for the present study and, if applicable, for the original study on which the present article is based	13

*Give information separately for cases and controls in case-control studies and, if applicable, for exposed and unexposed groups in cohort and cross-sectional studies.

Note: An Explanation and Elaboration article discusses each checklist item and gives methodological background and published examples of transparent reporting. The STROBE checklist is best used in conjunction with this article (freely available on the Web sites of PLoS Medicine at http://www.plosmedicine.org/, Annals of Internal Medicine at http://www.annals.org/, and Epidemiology at http://www.epidem.com/). Information on the STROBE Initiative is available at www.strobe-statement.org.

For peer review only - http://bmjopen.bmj.com/site/about/guidelines.xhtml

BMJ Open

Local area public sector spending and nutritional anaemia hospital admissions in England: a longitudinal ecological study

Journal:	*BMJ Open*
Manuscript ID	bmjopen-2021-059739.R1
Article Type:	Original research
Date Submitted by the Author:	11-Jul-2022

Complete List of Authors:	Jenkins, Rosemary; Imperial College London<br>Vamos, Eszter; Imperial College London, School of Public Health<br>Mason, Kate; University of Liverpool, Public Health & Policy<br>Daras, Konstantinos ; University of Liverpool, Public Health, Policy and Systems<br>Taylor-Robinson, David; University of Liverpool,<br>Bambra, Clare; Newcastle University<br>Millett, Christopher; Imperial College, Primary Care and Social Medicine<br>Laverty, Anthony; Imperial College London, Primary Care and Public Health

**Primary Subject Heading**:	Public health
Secondary Subject Heading:	Health policy
Keywords:	SOCIAL MEDICINE, PUBLIC HEALTH, Health policy < HEALTH SERVICES ADMINISTRATION & MANAGEMENT

SCHOLARONE™
Manuscripts

BMJ

I, the Submitting Author has the right to grant and does grant on behalf of all authors of the Work (as defined in the below author licence), an exclusive licence and/or a non-exclusive licence for contributions from authors who are: i) UK Crown employees; ii) where BMJ has agreed a CC-BY licence shall apply, and/or iii) in accordance with the terms applicable for US Federal Government officers or employees acting as part of their official duties; on a worldwide, perpetual, irrevocable, royalty-free basis to BMJ Publishing Group Ltd ("BMJ") its licensees and where the relevant Journal is co-owned by BMJ to the co-owners of the Journal, to publish the Work in this journal and any other BMJ products and to exploit all rights, as set out in our licence.

The Submitting Author accepts and understands that any supply made under these terms is made by BMJ to the Submitting Author unless you are acting as an employee on behalf of your employer or a postgraduate student of an affiliated institution which is paying any applicable article publishing charge ("APC") for Open Access articles. Where the Submitting Author wishes to make the Work available on an Open Access basis (and intends to pay the relevant APC), the terms of reuse of such Open Access shall be governed by a Creative Commons licence – details of these licences and which Creative Commons licence will apply to this Work are set out in our licence referred to above.

Other than as permitted in any relevant BMJ Author's Self Archiving Policies, I confirm this Work has not been accepted for publication elsewhere, is not being considered for publication elsewhere and does not duplicate material already published. I confirm all authors consent to publication of this Work and authorise the granting of this licence.

For peer review only

Local area public sector spending and nutritional anaemia hospital admissions in England: a longitudinal ecological study

Rosemary H. Jenkins[1], Eszter P Vamos[1], Kate E. Mason[2], Konstantinos Daras[2], David Taylor-Robinson[2], Clare Bambra[3], Christopher Millett[1], Anthony A. Laverty[1]

[1] Department of Primary Care and Public Health, School of Public Health, Imperial College London, Charing Cross Campus; The Reynolds Building; St Dunstan's Road; London, UK; W6 8RP.

[2] Department of Public Health, Policy and Systems, Institute of Population Health, University of Liverpool; Waterhouse Building Block F, 2nd Floor, Liverpool, UK; L69 3BX

[3] Population Health Sciences Institute, Faculty of Medical Sciences, Newcastle University, UK, NE1 4LE

Correspondence to: Dr Anthony Laverty, Public Health Policy Evaluation Unit, Department of Primary Care and Public Health, School of Public Health, Imperial College London, Charing Cross Campus; The Reynolds Building; St Dunstan's Road; London, UK; W6 8RP. a.laverty@imperial.ac.uk

For peer review only

ABSTRACT

Introduction: Reductions in local government spending may have impacts on diets and health which increase the risk of hospital admissions for nutritional anaemias. Mechanisms include potential impacts of changes to local authority (LA) services (e.g. housing services) on personal resources and food access, availability, and provision. We therefore investigated the association between changes in LA spending and nutritional anaemia related hospital admissions.

Design: Longitudinal analysis of LA panel data using Poisson fixed effects regression models.

Setting: 312 LAs in England (2005-2018).

Main exposure: Total LA service expenditure per capita per year.

Main outcome: Principal and total nutritional anaemia hospital admissions, for all ages and stratified by age (0-14, 15-64, 65+).

Results: LA service expenditure increased by 9% between 2005 and 2009 then decreased by 20% between 2010 and 2018. Total nutritional anaemia hospital admissions increased between 2005 and 2018 from 173 to 633 admissions per 100,000 population. A £100 higher LA service spending was associated with a 1.9% decrease in total nutritional anaemia hospital admissions (adjusted Incidence Rate Ratio: 0.98, 95% CI 0.96 to 0.99). When stratified by age, this was seen only in adults. A £100 higher LA service spending was associated with a 2.6% decrease in total nutritional anaemia hospital admissions in the most deprived LAs (aIRR: 0.97, 95% CI: 0.95 to 1.0).

Conclusion: Increased LA spending was associated with reduced hospital admissions for nutritional anaemia. Austerity related reductions had the opposite effect, increasing admissions, with greater

impacts in more deprived areas. This adds further evidence to the potential negative impacts of austerity policies on health and health inequalities. Re-investing in LA services may prevent hospital admissions associated with nutritional anaemias.

Strengths and limitations of this study

- This study uses national data to assess relationships between public sector spending and hospital admissions
- A fixed effect panel design allows us to examine within local authority changes in both exposures and outcomes
- However, we examined hospitalisations only, which does not give a full picture of burden of disease, as measures such as GP diagnoses would
- The natural experimental design means that we cannot infer causality and research at the individual level would assist with this in the future

For peer review only

INTRODUCTION

Nutritional anaemias related to poor diet are an important public health issue, increasing in prevalence over recent years. Nutritional anaemias involve reductions in red blood cells or their haemoglobin content due to a low nutritional intake of certain micronutrients, particularly iron, vitamin B-12, and folate(1). Nutritional anaemias are associated with a range of adverse health outcomes – for example, iron deficiency anaemia can lead to poor outcomes in those with coronary heart disease(2). Furthermore, nutrient deficiencies, which cause nutritional anaemias, are important risk factors for diseases through multiple physiological mechanisms(3, 4). For example, hospitalisations for iron deficiency anaemias increased from 67,592 in 2008-09 to 131,064 in 2016-17(5). A 2019 report by the UK House of Commons Environmental Audit Committee described the presence of a double burden of malnutrition in the UK, with overweight and obesity coexisting with undernutrition, both leading to micronutrient deficiencies(6).

The increase in hospital admissions for nutritional anaemias has occurred at the same time as large cuts to public services in the UK(5). In 2010, the UK government introduced austerity policies which led to considerable changes to the UK benefits system and reductions in local authority (LA) funding and service expenditure(7, 8). Reductions in LA funding had greater impacts in urban and more deprived councils, especially in the north of England, leading to differential impacts on the wide range of services that LAs provide(9-11). These services include social care, housing, highways and transport, environment and regulatory, and planning and development services(11). Systematic review evidence demonstrates that these services may be important in improving the social determinants of health and health inequalities(12). Research reports have found that reductions to LA service spending may have had negative impacts on health and widened health inequalities(13). For example, a £100 decrease in annual LA funding per person has been associated with a decrease in life expectancy at birth of 1.2-1.3 months between 2013-17(14).

The relationship between changes in local government spending and risk of nutritional anaemia is unclear. Much of the extant research on nutritional impacts of austerity policies in the UK has focused on foodbank use and not other potential outcomes such as hospital admissions(15). Risk of nutritional anaemias caused by poor diets may be plausibly impacted by changes to public sector spending through a range of different mechanisms. Decreases in adult social care expenditure, particularly in more deprived LAs, may have led to fewer residential and domiciliary care placements, which could have precipitated changes in food provision to vulnerable people if they are not able to access the food provided through such care placements(16). Decreases in Meals on Wheels services, lunch clubs, and other food provision may also have a similar effect(17, 18). These

For peer review only

services have been shown to be important in providing vulnerable adults with the calories and nutrients that they need, preventing malnutrition(19, 20). Thus, decreases in these services may directly lead to increases in nutritional anaemias in these individuals due to deficits in nutrients. Fewer carers visiting homes may also lead to issues with food acquisition as individuals may rely on carers for shopping, which may also lead to changes in food intakes and potential nutritional anaemias. Changes to LA environmental and regulatory spending and planning and development spending may influence access and availability of foods through changes to local businesses, hygiene inspections, and food regulation, which may also lead to changes in food purchasing, particularly takeaways(21).

There are also pathways which may indirectly lead to reductions in micronutrient consumption and nutritional anaemias. Through factors including less housing advice and support, less money spent on housing renewal, poorer quality homes and lower spending on homelessness, reductions in LA housing service spending may lead to trade-offs between housing costs and paying for other items, including healthy food, due to a reduction in personal resources(22, 23). As micronutrient-rich foods such as fruits and vegetables tend to be more expensive than ultra-processed foods which are generally low in micronutrients, these changes in resources may indirectly lead to nutritional anaemias through changes in types of foods purchased and consumed(24). Lower LA highways and transport spending may have similar impacts on personal resources and therefore food purchasing due to increased costs of public transport(11, 25, 26). Decreases in highways and transport spending may also lead to reductions in public transport (eg. number of different routes, number of busses per day etc.)(11, 25, 26). This may adversely affect individuals' access to supermarkets, potentially leading to decreases in nutritious foods, increases in ultra-processed foods, and increases in takeaways(27, 28). Finally, a decrease in money spent on cultural services (including recreation and sport, library services, culture and heritage and open spaces) may indirectly lead to micronutrient depletion through changes to lifestyle (such as physical activity) and potential increases in stress and mental health issues, though further research is needed in this area(21, 29). Thus, there are a number of potential pathways which may influence diagnoses of nutritional anaemias (Fig. 1). We therefore aimed to investigate the association between changes to LA service spending and principal and total nutritional anaemia hospital admissions in LAs in England. Given the role of LAs in addressing the social determinants of health, we hypothesise that effects may be stronger in more deprived LAs and those experiencing greater reductions in working age benefits.

METHODS

Data

For peer review only

Exposure variables

The time period for our analysis was 2005-2018. Annual LA service expenditure is reported by the Ministry of Housing, Communities & Local Government, compiled and freely available from the Place-based Longitudinal Data Resource (PLDR) for 326 LAs(30-36). These data are based on financial years rather than calendar years. We used net expenditure data for lower tier LAs, which excludes income from services. The Islands of Scilly and the City of London were not included due to small populations, and an additional 12 LAs were excluded due to missing covariate data. We calculated total net LA expenditure excluding police services and fire and rescue services (as these are funded by separate grants and precepts and provided by separate authorities), education services (due to changes in schooling provision over the study period), public health services (due to data not being available for all years due to changes in LA responsibilities) and court services (due to data not being available for all years)(37). All data were adjusted for inflation using the Consumer Price Index with 2015 as the reference year(38).

Outcome variables

Hospital Episode Statistics (HES) is a database of all NHS hospitals' admissions, A&E attendances, and outpatient appointments in England(39). For this study, we used yearly LA-level counts of inpatient or day-case admissions of nutritional anaemias in NHS hospitals available as safeguarded datasets from the PLDR(40-43). These were iron deficiency anaemia (excluding iron deficiency anaemia due to blood loss), vitamin B-12 deficiency anaemia, folate deficiency anaemia and other nutritional anaemias by LA. We obtained principal nutritional anaemia hospital admissions data where the nutritional anaemia was the "condition established after study to be chiefly responsible for occasioning the admission of the patient to the hospital for care"(44). We also obtained data for hospital admissions where nutritional anaemias are a secondary diagnosis, an additional condition that an individual may have during a hospital admission for a different principal diagnosis. These were given by each age (0-14, 15-64, 65+) or sex group individually. We aggregated these data to obtain principal nutritional anaemia hospital admissions and total nutritional anaemia hospital admissions (consisting of both principal and secondary diagnoses). In this paper, we focus on total nutritional anaemia hospital admissions as they key outcome for a number of reasons, including 1) principal nutritional anaemia hospital admissions are relatively uncommon, and thus total nutritional anaemia hospital admissions are more reflective of the burden of nutritional anaemias in the population and 2) the multisystem impact of nutritional anaemias means that the primary diagnoses may be considered to be a manifestation of nutritional anaemias as they led to the patient being ill

For peer review only

enough to seek medical care. We used annual LA population estimates from the Office for National Statistics (ONS) to generate admission rates per 100,000 population(45).

For sensitivity analyses, we also obtained counts of nutritional anaemia admissions excluding those alongside a principal or secondary diagnosis of another condition that may cause nutritional deficiencies, such as cachexia, malabsorption, malignancies and diabetes (full list in Appendix 1). As with the main outcome variable above, these data were obtained stratified by age or sex. These data were also aggregated to obtain principal and total (ie. principal and secondary) nutritional anaemia hospital admissions.

Covariates & Effect Modifiers

In the regression models we adjusted for the proportion of the LA population that is working age, proportion of LA population that is male, Gross Disposable Household Income (GDHI) and unemployment rate. The proportion of LA population that was working age (aged 15-64) and male were calculated from ONS data(45). These covariates were included to account for differences in changing LA demographics. We obtained GDHI data, an area-level measure of individuals' available money for spending or saving following payment of taxes and receipt of benefits, adjusted for inflation, from the ONS(46). LA unemployment rates were also obtained from the ONS, who calculate them based on a model utilising estimates from the Labour Force Survey and counts of people claiming unemployment benefits(47). GDHI and unemployment rate were used as markers of available household resources at the LA level. Time invariant factors were not adjusted for as they were removed by the fixed effects model, however, we also obtained Index of Multiple Deprivation (IMD) and level of reductions in working-age benefits as they were identified a priori as potential effect modifiers. IMD 2015 was obtained from the Ministry of Housing, Communities & Local Government(48). We used the population-weighted rank of average rank of LA IMD to make quintiles of relative deprivation. We also used the Uneven Impact of Welfare Reform dataset (provided by its authors) which estimated the cumulative reductions in working-age people's benefits for each LA due to welfare reforms since 2010(49). Previous research has found strong associations between reductions in welfare benefits and clinical outcomes(50). We derived quartiles of this variable to assess level of reductions to working-age benefits as a potential effect modifier.

Analyses

We descriptively examined total LA service expenditure in 2005, 2010, and 2018, including differences by covariates and proposed effect modifiers. We also calculated percentage changes before (2005-2009) and after (2010-2018) the introduction of austerity measures. We tabulated

For peer review only

mean principal and total nutritional anaemia hospital admissions for the full sample and stratified by age group, presented as rates per 100,000 population, and plotted their change over time. We also plotted change over time stratified by sex.

We used a Poisson fixed effects panel regression approach for the main analysis. Panel regression models can be used to investigate LA units over time while taking data clustering over time into account(51). Poisson fixed effects regression was used as a robust approach to modelling count panel data(52). We used cluster robust standard errors to adjust for potential autocorrelation and heteroskedasticity(53). We also created dummy variables for each year and used them to account for England-wide time effects. LA population was used as an offset variable to account for population size. For our primary analysis, we undertook a Poisson fixed effects analysis to investigate the relationship between total LA service spending and total nutritional anaemia hospital admissions. We present adjusted models (with covariates as proportion of the LA population that is working age, proportion of the LA population that is male, Gross Disposable Household Income (GDHI) and unemployment rate). We also subsequently stratified by IMD quintiles and level of benefit reductions quartiles to test a priori hypotheses regarding effect modification. We undertook analyses stratified by age and sex. We separately analysed principal nutritional anaemia hospital admissions as the outcome, with adjusted and stratified Poisson models as above.

We also undertook two sensitivity analyses. In the first, we used as the outcome variable total nutritional anaemia hospital admissions excluding admissions alongside a diagnosis of another condition which may cause a nutritional deficiency. While public sector spending reductions could cause or exacerbate this wider set of conditions, this sensitivity analysis focuses on diagnoses of being admitted *for* nutritional anaemia rather than *with* it. In the second sensitivity analysis, we excluded social care expenditure from the exposure variable. Unlike most areas of LA service expenditure, some LAs had to increase their social care expenditure after 2010 due to demand. Thus, LAs with higher spending on social care may have higher levels of nutritional anaemias, which may lead to issues of reverse causality when social care is included in the exposure variable(54). Our sensitivity analysis investigated the relationship between LA spending excluding social care and total nutritional anaemia hospital admissions.

Patient and Public Involvement

Plans for this research were presented to a committee of three public experts in November 2020. The public experts provided feedback on the research plans and appropriate changes were made following the meeting.

For peer review only

RESULTS

On average, LA service spending increased by 9% between 2005 and 2009, and then decreased by 20% between 2010 and 2018 (Table 1). Mean total LA spending per year is plotted in Appendix 2. Between 2005 and 2009, LAs in Yorkshire and the Humber (16%), the East Midlands (15%), and the North West (14%) increased expenditure the most, while London (4%) and the South East (5%) increased expenditure the least. However, LAs with the highest unemployment rates decreased expenditure between 2005-2009. Following 2010, London had the greatest decrease of 31%, followed by the Northern regions (a 27% decrease for the North East and 21% for both the North West and Yorkshire and the Humber). More deprived LAs experienced greater cuts (28% in the most deprived quintile compared to 12% for the least deprived quintile). Greater reductions were also seen in areas with higher unemployment rates and larger reductions to working age benefits.

Total nutritional anaemia hospital admissions have increased since 2005, from 173 per 100,000 population in 2005 to 632 per 100,000 population in 2018 (Fig. 2). Total nutritional anaemia hospital admissions in 65+ adults increased between 2005-2010, with the rate of increase slowing between 2010-2013, and then increasing more steeply between 2014 and 2018. Rate of admissions in 65+s was 684 per 100,000 population in 2005 and 2,103 per 100,000 in 2018. There was also an increase throughout the study period for 15-64-year olds, from 88 per 100,000 population in 2005 to 364 per 100,000 population. Total nutritional anaemia hospital admissions also increased slightly in 0-14s between 2005 and 2018, from 19 to 57 admissions per 100,000 population. Rates of admissions also increased for both men and women when stratified by sex, and women had a higher rate of nutritional anaemias than men (Fig. 3).

In 2018, the rate of principal nutritional anaemia hospital admissions was 236 per 100,000 population (Table 2). Rates increased with age (11 per 100,000 population for 0-14s, 154 for 15-64s, and 717 for 65+s). The rate of total nutritional anaemia hospital admissions in 2018 was 632 per 100,000 population. Rates of total nutritional anaemia hospital admissions also increased with age – rates were relatively low in children (57 per 100,000 population) but were 364 and 2,102 per 100,000 population in adults (15-64 and 65+ respectively). Principal and total nutritional anaemia hospital admissions increased with deprivation for all age groups. Rates of admissions increased with unemployment and level of benefit reductions and decreased with GDHI.

Poisson fixed effects analysis of the association between total LA service spending and principal and total nutritional anaemia hospital admissions

A £100 per capita higher LA service spending was associated with 1.9% lower total nutritional anaemia hospital admissions (adjusted IRR: 0.981, 95% CI: 0.964, 0.999) (Table 3). This relationship was only statistically significant in adults aged ≥15 years. A £100 higher LA service spend per capita was associated with 2.2% lower total nutritional anaemia hospital admissions in the adjusted model for people aged 15-64, and 2.1% lower rates in 65+ respectively (IRR for 15-64: 0.978, 95% CI: 0.959, 0.998; IRR for 65+: 0.979, 95% CI: 0.960, 0.997). When stratified by IMD, this relationship was statistically significant in the 1st (most deprived) (adjusted IRR for all age groups: 0.974, 95% CI: 0.951, 0.998) and 4th (adjusted IRR for all age groups: 0.883, 95% CI: 0.831, 0.939) quintiles. This persisted in adults. When stratified by level of reductions to working age benefits, this relationship was present in the 2nd quartile (adjusted IRR for all age groups: 0.963, 95% CI: 0.935, 0.991) and 4th quartile for 65+ (adjusted IRR for 65+: 0.973, 95% CI: 0.947, 0.999).

When we investigated this relationship stratified by sex, we found that this relationship was statistically significant for males only (Table 4). A £100 per capita higher LA service spending was associated with 2.5% lower total nutritional anaemia admissions in males (adjusted IRR 0.975, 95% CI: 0.957, 0.993). Similarly to above, the relationship was statically significant in the 1st (adjusted IRR: 0.968, 95% CI: 0.944, 0.993) and 4th (adjusted IRR: 0.872, 95% CI: 0.817, 0.932) IMD quintiles for males, but none of the results were statistically significant for females when stratified by IMD. When stratified by reductions to working age benefits, higher LA spending was statistically significantly associated with lower hospital admissions for nutritional anaemias in the 2nd (adjusted IRR: 0.955, 95% CI: 0.926, 0.985) and 4th (adjusted IRR: 0.970, 95% CI: 0.942, 1.000) quartiles for males only.

When we investigated this relationship with principal rather than total nutritional anaemia hospital admissions as the outcome, this relationship was no longer statistically significant (adjusted IRR: 0.987, 95% CI: 0.963, 1.011) (Appendix 3). LA spending was not statistically significantly associated with principal nutritional anaemia hospital admissions in any of the age groups: 0-14 (adjusted IRR: 0.996, 95% CI: 0.945, 1.051), 15-64 (adjusted IRR: 0.986, 95% CI: 0.957, 1.015), and 65+ (adjusted IRR: 0.983, 95% CI: 0.957, 1.009). When stratified by IMD, a statistically significant relationship was present for the 4th quintile only (eg. IRR for all ages: 0.838, 95% CI: 0.759, 0.925).

Sensitivity analysis: excluding diagnoses which may cause a nutritional deficiency

The increase in nutritional anaemias over time (Fig. 2) was much less steep when nutritional anaemia admissions alongside a diagnosis of a condition which may cause a nutritional deficiency were excluded (Appendix 4). When nutritional anaemia hospital admissions that were alongside a principal or secondary diagnosis of another condition which may cause a nutritional deficiency were excluded in the Poisson regression analysis, generally the relationship between LA spending and

For peer review only

total nutritional anaemia hospital admissions was not statistically significant (for example, adjusted IRR for all age groups: 0.991, 95% CI: 0.974, 1.009) (Appendix 5). There were some significant relationships in the middle IMD and level of reductions to working age benefits (eg. adjusted IRR for quintile 2 of reductions to working age benefits for 15-64: 0.960, 95% CI: 0.924, 0.997).

Sensitivity analysis: total LA service expenditure excluding social care

When social care was excluded from the exposure variable, a £100 increase in LA service spending was associated with a 4.0% decrease in total nutritional anaemia hospital admissions (adjusted IRR: 0.960, 95% CI: 0.939, 0.982) (Appendix 6). This relationship remained in the 15-64 age group (adjusted IRR for 15-64: 0.958, 95% CI: 0.934, 0.983) and the 65+ age group (adjusted IRR for 65+: 0.958, 95% CI: 0.936, 0.980) when stratified by age (Appendix 7). Statistically significantly greater reductions in nutritional anaemias were seen in the first and fourth IMD quintiles, suggesting effect modification by deprivation. Little effect modification was seen by level of reductions to working age benefits.

DISCUSSION

This analysis of data from 2005-18 found that reductions in spending due to austerity policies may have increased nutritional anaemia hospital admissions, especially in more deprived areas. LA service spending increased by 9% between 2005 and 2009 then decreased by 20% between 2010 and 2018, following the introduction of austerity policies in 2010. Total nutritional anaemia hospital admissions also increased between 2005 and 2018. We found that a £100 higher LA service spending was associated with 1.9% lower total nutritional anaemia hospital admissions. This was driven by admissions in adults. A 2.6% lower admission rate was seen with a £100 higher expenditure in the most deprived LAs, suggesting greater impacts by deprivation, especially since these LAs experienced greater service spending reductions overall (a 28% reduction was seen in the most deprived quintile compared to a 12% reduction in the least deprived quintile between 2010 and 2018). However, we also found that a £100 higher expenditure was statistically significantly associated with a reduction in admissions in quintile 4 (one of the least deprived quintiles), suggesting that there may be impacts across the spectrum of LAs in terms of deprivation. Regardless of the relative effects of spending across levels of deprivation, the far greater cuts to spending in more deprived areas mean the absolute impact on nutritional anaemias will have been felt disproportionately in these areas.

We found that total nutritional anaemia hospital admissions increased between 2005 and 2018. This aligns with previous research suggesting decreases in micronutrient consumption and corresponding

increases in nutritional anaemias in the UK(5, 55, 56). We identified an overall increase in total nutritional anaemia hospital admissions which plateaued between 2010-2013, and then increased again. Research has found that between 2007 and 2011 the percentage of patients known to have had nutritional screening increased from 67-78% to 86-95%(57). Therefore, the increase in nutritional anaemia hospital admissions between 2005-2010 may be an artefact of increased screening over this period, with the plateau over the following years representing nutritional screening reaching near-saturation of hospital admissions. Given that nutritional screening was approaching saturation as austerity was being rolled out, an increase in nutritional anaemias after 2010 - during austerity - may be in part explained by changes in LA spending as per our hypothesis.

We found that the impact of LA spending on nutritional anaemia admissions was statistically significant in males but not females. This may be due to biological mechanisms – males have higher dietary reference values than women for most micronutrients (with the exception of iron to cover for iron losses during menstruation for women), although there is a lack of nuance in these reference values with regards to more subtle differences in growth and maturation rates and health vulnerabilities(58). Analyses of UK National Diet and Nutrition Survey data have found that women were more vulnerable than men to micronutrient deficiencies. It is possible that some women vulnerable to micronutrient deficiencies were already likely to be admitted with nutritional anaemias prior to austerity policies, particularly due to monitoring during pregnancy, whereas these impacts may have pushed men who were vulnerable to deficiencies into being admitted with nutritional anaemias. With regards to differences in causal pathways, it has been suggested that women have been more affected by the UK government's austerity policies than men(59). Thus, we might have expected to see greater or more statistically significant effects in women. Further individual level research, taking into account causal pathways, is needed to understand these sex differences.

Our study suggests that these decreases in LA service spending may have health impacts. This supports other studies which suggest that decreases in LA service spending may adversely affect health and mortality(14, 60). Our research adds new evidence that there may be associations with nutritional anaemias in addition to other health outcomes previously examined. As nutritional anaemias are important risk factors for diseases, this may be one pathway through which reductions in LA service expenditure may be associated with health. More deprived areas also experienced greater reductions in LA service spending, meaning the absolute increase in nutritional deficiencies attributable to service spending cuts will have been greater in more deprived areas, which already have greater health needs. This aligns with other evidence that austerity measures had a greater

For peer review only

impact in more deprived areas and may have widened health inequalities due to the role of LA services in improving the social determinants of health(9, 10, 12, 13).

This research has several strengths. We are the first to examine associations between austerity policies and nutritional anaemia hospital admissions. We used a large panel dataset with coverage of all NHS hospitals for 312 LAs in England, over a long time period (2005-2018). We used a fixed effects modelling strategy and were able to account for all time-invariant factors that differ between LAs and changing population sizes, allowing us to assess within-LA change in hospital admissions over time. Furthermore, our sensitivity analysis found a stronger relationship between total LA service spending and total nutritional anaemia hospital admissions when social care spending was excluded. This suggests that social care spending may be blunting the relationship between LA service spending and total nutritional anaemia hospital admissions, likely due to higher levels of nutritional anaemias requiring higher levels of social care spending(54). This further supports our hypothesis that austerity measures may have led to increased nutritional anaemia hospital admissions.

Our study also has some limitations. As previously described, we used counts of nutritional anaemia hospital admissions, which may be an artefact of hospital processes such as more screening leading to more diagnoses. As we used data on hospital admissions, we did not examine diagnoses by GPs, which may give a more complete picture of potential impacts on nutritional anaemias at the population level. We were also unable to break admissions down by severity, or to access data from laboratory tests, which may have picked up more cases of anaemia than were coded in the HES data. Further research could usefully explore these issues, as well as potential impacts of anaemia on the need for transfusions. Furthermore, the relationship we have described was only statistically significant when total nutritional anaemia hospital admissions were included as the outcome measure and not when principal nutritional anaemia hospital admissions were the outcome. Therefore, it is possible that the relationship seen may be a result of LA service spending being associated with increases in people accessing hospitals for other health conditions and nutritional anaemias also being present, leading to more total nutritional anaemia hospital admissions which would otherwise have been in the community and undiagnosed. One possibility is that such increases in hospital access may be due to more general impacts of austerity policies on health outcomes. Additionally, our exposure data were per financial year and our outcome data were per calendar year, representing a potential mismatch in time periods assessed. Another limitation is that we did not obtain outcome data by both age and sex.

For peer review only

Sensitivity analyses excluding hospital admissions alongside a medical condition which may cause a nutritional deficiency did not identify a relationship between LA service spending and total nutritional anaemia hospital admissions. This suggests that the relationship between LA spending and nutritional anaemia hospital admissions is driven by changes in the prevalence of these other conditions. The degree to which nutritional anaemias may be caused by other medical conditions is difficult to assess in routine data without more detailed clinical parameters and we excluded a range of common conditions such as cancers and type 2 diabetes where nutritional deficiencies are common. These diseases have a nutritional component, particularly with regards to their association with obesity, and thus may represent wide-ranging impacts of austerity policies on nutrition in terms of both nutritional anaemias and chronic disease. Together our analyses suggest that the burden of nutritional anaemias is increasing over time and that this is more apparent in areas with greater levels of deprivation, greater levels of medical comorbidity, and potentially in areas with more severe reductions in LA spending(61).

Our panel study suggests that reductions in LA service spending due to UK austerity policies may be associated with an increase in nutritional anaemias. This adds further evidence to the growing evidence base regarding potential impacts of these policies on health and health inequalities. Nutritional anaemias have wide-ranging impacts on health. Thus, policy-makers should consider associations between fiscal policies and health prior to their introduction. This is particularly pertinent given the budget deficit as well as possible further reductions in LA budgets alongside tax rises coupled with a emerging cost of living crisis. In the context of the proposed levelling-up agenda in England, this study adds more evidence to support the need for reinvestment in LA services as a means to improve population health outcomes and reduce health inequalities(62). Nutritional anaemias fell between 2005 and 2009, a period of a national health inequalities strategy in England which entailed sustained investment in LAs, the NHS, prevention and the welfare system (including tax credits, supplemental funds for pregnant women, and increases in the national pension)(63). Policy-makers should draw on this past success and consider including health outcomes in the allocation criteria for levelling-up investments(64). Indeed, a national health inequalities strategy is once more needed to level up health across the country. However, individual-level research is needed to elucidate pathways between changes to LA service spending and nutritional anaemias, particularly with regards to different areas of LA service spending.

DATA AVAILABILITY STATEMENT

All data are available on the PLDR resource (pldr.org)

ETHICS STATEMENT

Patient Consent for Publication

For peer review only

Not required.

Ethics Approval

Ethical approval was granted for this study by Imperial College Research Ethics Committee (ICREC).

ACKNOWLEDGEMENTS

We are grateful to Christina Beatty and Steve Fothergill for sharing their data from The Uneven Impact of Welfare Reform project.

COMPETING INTERESTS

We have no competing interests to declare.

FUNDING

This study is funded by the National Institute for Health Research (NIHR) School for Public Health Research (SPHR), Grant Reference Number PD-SPH-2015. DTR is funded by the MRC on a Clinician Scientist Fellowship (MR/P008577/1). KD is supported by the National Institute for Health Research (NIHR) Applied Research Collaboration North West Coast (ARC NWC, NIHR200182).

Contributions

Study conception and design by RHJ, EPV, KEM, AAL. Data analysis was by RHJ with input from EPV, KEM and AAL. RHJ wrote the first draft and all other authors (EPV, KEM, KD, DTR, CM, CM and AAL) made significant contributions to the final version.

Figure legends

Figure 1: Logic model of potential pathways through which changes to LA spending may affect nutritional anaemias

Figure 2: Rates of principal and total nutritional anaemia hospital admissions by year, stratified by age.

Figure 3: Rates of principal and total nutritional anaemia hospital admissions by year, stratified by sex

For peer review only

Table 1: Mean LA service expenditure in 2005, 2010, and 2018 and percentage change, stratified by socio-demographic variables.

	Total per capita LA spending 2005	Total per capita LA spending 2010	Total per capita LA spending 2018	Change 2005-2009	Change 2010-2018	Change 2005-2018
**Total**	845.4 (237.4)	861.6 (202.7)	690.9 (103.4)	+9.2%	-19.8%	-18.3%
**Region**						
North East	1007.0 (153.9)	1060.7 (166.8)	776.7 (78.6)	+11.5%	-26.8%	-22.9%
North West	882.7 (192.5)	943.9 (187.0)	741.7 (82.9)	+14.4%	-21.4%	-16.0%
Yorkshire & the Humber	773.1 (106.5)	839.5 (119.4)	661.7 (77.1)	+16.1%	-21.2%	-14.4%
East Midlands	705.0 (64.3)	776.7 (111.0)	620.0 (72.0)	+14.5%	-20.1%	-12.1%
West Midlands	770.2 (98.7)	768.7 (120.3)	655.8 (73.1)	+5.5%	-14.7%	-14.9%
London	1399.2 (272.7)	1263.1 (236.8)	870.0 (128.1)	+3.6%	-31.1%	-37.8%
South West	767.9 (64.2)	805.0 (74.5)	678.9 (52.0)	+10.0%	-15.7%	-11.6%
East of England	764.0 (48.9)	801.7 (49.9)	658.3 (42.1)	+11.1	-17.9%	-13.8%
South East	754.0 (81.3)	742.9 (75.1)	651.6 (61.9)	+4.7%	-12.3%	-13.6%
**Percentage male**						
<48.7%	825.5 (169.8)	851.5 (172.3)	687.6 (86.2)	+10.0%	-19.3%	-16.7%
48.7%-49.1%	855.1 (240.0)	859.5 (192.8)	658.8 (71.0)	+8.0%	-23.4%	-23.0%
49.1%-49.5%	811.2 (201.6)	834.0 (173.9)	674.7 (85.1)	+9.6%	-19.1%	-16.8%
>49.5%	907.9 (350.1)	906.6 (262.9)	725.5 (131.7)	+8.2%	-20.0%	-20.1%
**Percentage working age**						
<62.6%	761.2 (60.2)	786.8 (73.6)	659.6 (69.7)	+8.4%	-16.2%	-13.4%
62.6%-64.5%	782.8 (141.0)	771.7 (111.8)	687.7 (84.2)	+4.0%	-10.9%	-12.2%
64.5%-66.3%	786.7 (117.9)	838.9 (153.9)	700.7 (93.1)	+13.2%	-14.5%	-10.9%
>66.3%	985.5 (347.5)	995.5 (275.0)	812.2 (151.8)	+7.6%	-18.4%	-17.6%
**GDHI per capita[1]**						
<£15,892	884.8 (198.6)	956.1 (174.3)	740.0 (94.0)	+13.7%	-22.6%	-16.4%
£15,892-£18,346	782.9 (154.5)	795.8 (94.7)	659.9 (72.3)	+11.7%	-17.1%	-15.7%
£18,346-£21,422	824.6 (245.3)	816.3 (173.0)	656.8 (67.0)	+6.0%	-19.5%	-20.4%
>£21,422	893.9 (320.5)	891.9 (297.1)	708.5 (123.8)	+7.2%	-20.6%	-20.7%
**Unemployment rate**						
<3.8%	722.9 (48.7)	785.9 (75.8)	658.2 (70.2)	+19.9%	-16.3%	-9.0%
3-8%-5.1%	802.5 (117.0)	732.2 (58.6)	690.2 (92.8)	-2.1%	-5.7%	-14.0%
5.1%-6.8%	926.7 (177.4)	761.0 (83.8)	801.8 (141.5)	-12.3%	+5.4%	-13.5%
>6.8%	1352.3 (335.9)	972.6 (227.9)	842.4 (64.0)	-22.8%	-13.4%	-37.7%
**IMD**						
1 (most deprived)	1068.2 (326.0)	1084.2 (242.4)	782.5 (121.7)	+10.6%	-27.8%	-26.8%
2	883.2 (246.4)	914.2 (201.6)	713.0 (116.2)	+10.5%	-22.0%	-19.3%
3	798.0 (136.5)	809.0 (97.1)	664.0 (54.3)	+7.6%	-17.9%	-16.8%
4	746.4 (120.1)	769.5 (102.1)	647.3 (74.5)	+10.1%	-15.5%	-13.3%
5 (least deprived)	732.1 (89.6)	733.1 (82.6)	648.0 (62.0)	+6.8%	-11.6%	-11.5%
**Level of reductions to working age benefits per capita**						
1 (<£457)	726.6 (84.4)	731.3 (82.1)	643.6 (65.9)	+7.4%	-12.0%	-11.4%
2 (£457-£575)	805.8 (222.8)	819.3 (186.4)	675.5 (96.0)	+8.6%	-17.6%	-16.2%
3 (£575-£690)	868.8 (261.0)	885.8 (197.0)	694.2 (107.6)	+9.2%	-21.6%	-20.1%
4 (>£690)	985.3 (262.5)	1013.4 (208.1)	751.7 (108.8)	+11.3%	-25.8%	-23.7%

[1] GDHI is Gross Disposable Household Income per capita per year, an area-level measure of individuals' available money for spending or saving following payment of taxes and receipt of benefits

[2] Index of Multiple Deprivation, quintiles based on relative ranking of LAs

For peer review only

Table 2: Mean rate of principal and total nutritional anaemia hospital admissions per 100,000 LA population in 2018, stratified by socio-demographic variables.

	Mean rate of nutritional anaemia hospital admissions per 100,000 LA population in 2018 Mean (SE in brackets)							
	All ages		0-14		15-64		65+	
	Principal nutritional anaemia hospital admissions	Total nutritional anaemia hospital admissions	Principal nutritional anaemia hospital admissions	Total nutritional anaemia hospital admissions	Principal nutritional anaemia hospital admissions	Total nutritional anaemia hospital admissions	Principal nutritional anaemia hospital admissions	Total nutritional anaemia hospital admissions
**Total**	236.4 (95.8)	632.4 (213.7)	11.1 (16.9)	56.8 (41.1)	154.1 (65.0)	363.6 (145.8)	717.1 (278.3)	2101.8 (765.5)
**Region**								
North East	306.7 (106.1)	938.4 (268.3)	13.5 (6.9)	67.7 (26.2)	208.6 (79.3)	553.2 (210.4)	898.1 (329.1)	2997.9 (851.1)
North West	284.5 (88.6)	836.4 (200.5)	22.4 (35.4)	83.9 (62.5)	189.6 (72.8)	492.8 (172.2)	854.7 (308.1)	2688.0 (753.8)
Yorkshire & the Humber	278.3 (73.7)	698.0 (149.8)	7.9 (9.9)	55.2 (36.5)	181.9 (55.4)	404.8 (127.4)	804.8 (214.0)	2154.9 (374.2)
East Midlands	206.7 (86.1)	531.5 (179.1)	6.7 (8.8)	35.7 (29.0)	136.7 (61.1)	299.9 (113.1)	596.0 (261.4)	1677.8 (557.2)
West Midlands	211.0 (70.6)	584.5 (155.0)	11.1 (10.7)	61.9 (42.6)	146.5 (52.8)	347.9 (113.2)	585.8 (199.5)	1788.7 (503.3)
London	183.2 (62.5)	644.2 (140.2)	13.7 (9.1)	85.4 (29.3)	137.5 (49.4)	415.1 (109.8)	719.9 (209.0)	2956.2 (763.2)
South West	271.2 (119.0)	628.3 (204.4)	6.8 (8.4)	43.2 (28.7)	164.0 (69.0)	332.6 (108.1)	730.1 (313.1)	1825.1 (592.2)
East of England	255.2 (115.1)	620.2 (217.7)	9.8 (14.3)	47.7 (32.1)	157.2 (72.1)	354.6 (127.1)	786.0 (304.0)	1985.4 (582.0)
South East	205.8 (71.6)	515.4 (150.5)	9.5 (14.2)	48.1 (34.5)	127.7 (48.1)	276.6 (90.3)	651.3 (232.8)	1758.6 (571.3)
**Percentage male**								
<48.7%	238.0 (99.8)	614.5 (216.4)	9.7 (13.4)	48.9 (32.3)	144.9 (60.9)	332.2 (127.0)	662.1 (249.0)	1834.3 (581.6)
48.7%-49.1%	250.4 (100.3)	646.5 (206.5)	8.1 (9.6)	47.3 (31.1)	153.4 (61.9)	351.9 (128.1)	725.0 (298.2)	1981.2 (625.1)
49.1%-49.5%	240.4 (104.9)	633.1 (248.4)	12.3 (16.5)	57.9 (43.3)	159.7 (76.5)	364.8 (170.2)	725.3 (305.2)	2082.4 (844.3)
>49.5%	223.8 (81.3)	635.5 (186.0)	12.9 (22.0)	66.9 (47.5)	156.0 (59.4)	390.7 (142.7)	742.7 (260.1)	2372.5 (805.6)
**Percentage working age**								
<62.6%	241.5 (103.4)	624.5 (223.7)	9.2 (14.3)	47.8 (37.5)	148.4 (65.3)	337.0 (139.1)	662.6 (275.8)	1826.9 (616.5)
62.6%-64.5%	256.9 (85.8)	681.2 (223.6)	12.0 (11.2)	60.9 (38.5)	174.3 (67.9)	411.4 (167.4)	801.5 (259.2)	2278.9 (690.9)
64.5%-66.3%	223.0 (85.1)	591.4 (175.7)	17.8 (37.8)	75.1 (59.1)	163.7 (66.6)	381.9 (137.9)	763.8 (298.2)	2230.1 (704.6)
>66.3%	191.5 (71.8)	609.0 (168.0)	12.6 (9.5)	72.8 (35.2)	136.1 (49.1)	375.1 (115.6)	757.2 (266.2)	2799.7 (907.6)
**GDHI**[1]								

For peer review only

<£15,892	301.8 (102.0)	817.8 (244.4)	19.4 (30.4)	86.8 (59.3)	208.7 (69.3)	507.4 (180.5)	889.2 (281.3)	2628.6 (746.9)
£15,892-£18,346	241.0 (103.8)	649.8 (221.2)	10.1 (9.4)	52.1 (31.5)	156.2 (67.1)	368.4 (135.5)	704.1 (288.3)	2066.1 (692.3)
£18,346-£21,422	235.7 (90.7)	598.8 (172.8)	9.3 (12.9)	45.7 (30.2)	151.3 (57.2)	331.2 (103.6)	692.8 (295.8)	1911.2 (630.6)
>£21,422	200.6 (70.5)	549.8 (155.5)	9.1 (12.6)	53.2 (36.1)	126.9 (48.3)	310.9 (109.1)	656.3 (219.9)	1995.6 (804.9)
**Unemployment rate**								
<3.8%	230.6 (96.5)	579.0 (191.7)	8.3 (13.1)	44.1 (31.3)	140.3 (61.3)	310.7 (111.1)	673.5 (278.9)	1793.7 (579.8)
3-8%-5.1%	242.8 (92.5)	656.6 (203.7)	12.1 (21.6)	63.4 (47.2)	164.3 (60.5)	387.9 (131.7)	750.3 (276.9)	2226.3 (670.2)
5.1%-6.8%	237.5 (102.4)	752.4 (242.7)	18.9 (16.1)	84.0 (39.0)	175.0 (76.7)	482.5 (179.4)	786.8 (256.0)	2911.8 (887.9)
>6.8%	279.4 (84.1)	856.6 (256.7)	19.7 (8.8)	117.9 (28.0)	221.2 (60.6)	602.4 (184.1)	867.9 (297.3)	2873.1 (706.2)
**IMD[1]**								
1 (most deprived)	254.9 (99.1)	764.9 (230.5)	20.2 (28.0)	90.5 (51.6)	186.5 (75.1)	493.9 (173.0)	817.3 (267.4)	2781.7 (775.2)
2	246.6 (113.1)	670.4 (234.7)	11.1 (9.3)	61.4 (36.6)	165.0 (66.7)	390.3 (138.7)	742.9 (321.4)	2252.2 (847.3)
3	241.1 (89.3)	622.9 (177.6)	11.0 (13.5)	51.9 (32.2)	156.2 (57.2)	350.2 (103.7)	710.8 (275.3)	1993.3 (602.7)
4	238.8 (94.4)	595.1 (180.2)	7.9 (13.6)	41.9 (30.8)	142.8 (59.6)	314.4 (106.7)	692.2 (277.3)	1829.7 (541.6)
5 (least deprived)	202.3 (73.3)	512.3 (153.8)	5.5 (8.9)	38.5 (28.1)	120.9 (45.6)	271.2 (82.4)	625.1 (214.7)	1665.2 (467.5)
**Level of reductions to working age benefits per capita**								
1 (<£457)	206.2 (75.7)	521.9 (150.1)	5.3 (8.5)	39.4 (30.0)	123.0 (46.8)	275.7 (82.5)	630.4 (231.7)	1679.5 (484.8)
2 (£457-£575)	238.8 (100.4)	610.6 (196.9)	11.5 (16.1)	50.2 (33.5)	145.3 (60.7)	327.4 (111.3)	703.0 (284.4)	1962.2 (717.5)
3 (£575-£690)	239.5 (105.0)	639.8 (201.6)	9.3 (8.6)	51.6 (30.9)	158.2 (62.7)	366.4 (115.2)	744.6 (305.4)	2208.3 (748.8)
4 (>£690)	263.3 (92.7)	759.8 (233.6)	18.9 (26.3)	88.0 (51.4)	192.4 (70.0)	489.5 (172.6)	795.5 (263.7)	2571.1 (801.0)

[1] GDHI is Gross Disposable Household Income per capita per year, an area-level measure of individuals' available money for spending or saving following payment of taxes and receipt of benefits

[2] Index of Multiple Deprivation, quintiles based on relative ranking of LAs

For peer review only

For peer review only - http://bmjopen.bmj.com/site/about/guidelines.xhtml

Table 3: Association between LA service spending and total nutritional anaemia hospital admissions, shown as the Incident Rate Ratio and stratified by age (95% Confidence Intervals in brackets)

	Incidence Rate Ratio for total nutritional anaemia hospital admissions by LA with an £100 increase in total LA service spending			
	All ages	0-14	15-64	65+
Full Sample[1]	0.981 (0.964, 0.999) p=0.038	0.974 (0.904, 1.050) p=0.488	0.978 (0.959, 0.998) p=0.033	0.979 (0.960, 0.997) p=0.024
IMD[2]				
1 (most deprived)	0.974 (0.951, 0.998) p=0.032	0.943 (0.834, 1.066) p=0.348	0.971 (0.943, 0.999) p=0.043	0.974 (0.950, 0.999) p=0.038
2	0.991 (0.949, 1.035) p=0.678	1.030 (0.980, 1.082) p=0.251	0.999 (0.957, 1.043) p=0.960	0.982 (0.936, 1.030) p=0.452
3	1.016 (0.976, 1.058) p=0.431	1.032 (0.938, 1.135) p=0.524	1.019 (0.968, 1.073) p=0.472	1.006 (0.966, 1.047) p=0.779
4	0.883 (0.831, 0.939) p<0.001	0.929 (0.790, 1.092) p=0.371	0.851 (0.792, 0.915) p<0.001	0.891 (0.838, 0.946) p<0.001
5 (least deprived)	1.011 (0.955, 1.070) p=0.712	1.014 (0.860, 1.195) p=0.870	0.989 (0.922, 1.063) p=0.768	1.018 (0.966, 1.072) p=0.505
Level of reductions to working age benefits per capita				
1 (lowest reductions)	1.011 (0.961, 1.063) p=0.680	1.013 (0.877, 1.170) p=0.864	1.000 (0.946, 1.056) p=0.985	1.013 (0.962, 1.067) p=0.616
2	0.963 (0.935, 0.991) p=0.010	0.955 (0.889, 1.026) p=0.208	0.954 (0.920, 0.989) p=0.010	0.963 (0.935, 0.992) p=0.012
3	0.992 (0.952, 1.032) p=0.671	1.029 (0.928, 1.141) p=0.587	0.995 (0.955, 1.036) p=0.803	0.985 (0.942, 1.030) p=0.500
4 (greatest reductions)	0.975 (0.949, 1.003) p=0.077	0.935 (0.824, 1.061) p=0.294	0.973 (0.941, 1.006) p=0.104	0.973 (0.947, 0.999) p=0.043

[1] Adjusted by percentage working age, percentage male, GDHI and unemployment rate.
[2] Index of Multiple Deprivation, quintiles based on relative ranking of LAs.

For peer review only

Table 4: Impact of LA service spending on total nutritional anaemia hospital admissions, shown as the Incident Rate Ratio and stratified by sex (95% Confidence Intervals in brackets)

	Incidence Rate Ratio for total nutritional anaemia hospital admissions by LA with an £100 increase in total LA service spending		
	All Ages	Male	Female
Full Sample[1]	0.981 (0.964, 0.999) p=0.038	0.975 (0.957, 0.993) p=0.007	0.992 (0.974, 1.010) p=0.358
IMD[2]			
1 (most deprived)	0.974 (0.951, 0.998) p=0.032	0.968 (0.944, 0.993) p=0.012	0.980 (0.956, 1.005) p=0.118
2	0.991 (0.949, 1.035) p=0.678	0.988 (0.947, 1.030) p=0.563	1.005 (0.962, 1.050) p=0.818
3	1.016 (0.976, 1.058) p=0.431	1.009 (0.962, 1.058) p=0.707	1.032 (0.989, 1.076) p=0.151
4	0.883 (0.831, 0.939) p<0.001	0.872 (0.817, 0.932) p<0.001	0.902 (0.851, 0.956) p<0.001
5 (least deprived)	1.011 (0.955, 1.070) p=0.712	1.001 (0.936, 1.071) p=0.967	1.029 (0.967, 1.094) p=0.367
Level of reductions to working age benefits per capita			
1 (lowest reductions)	1.011 (0.961, 1.063) p=0.680	1.013 (0.962, 1.067) p=0.623	1.020 (0.962, 1.083) p=0.506
2	0.963 (0.935, 0.991) p=0.010	0.955 (0.926, 0.985) p=0.004	0.981 (0.951, 1.011) p=0.210
3	0.992 (0.952, 1.032) p=0.671	0.983 (0.944, 1.024) p=0.419	0.999 (0.960, 1.040) p=0.960
4 (greatest reductions)	0.975 (0.949, 1.003) p=0.077	0.970 (0.942, 1.000) p=0.048	0.986 (0.957, 1.015) p=0.330

[1] Adjusted by percentage working age, percentage male, GDHI and unemployment rate.
[2] Index of Multiple Deprivation, quintiles based on relative ranking of LAs

For peer review only

REFERENCES

1. World Health Organisation. Anaemia 2021 [Available from: https://www.who.int/health-topics/anaemia#tab=tab_2.
2. von Haehling S, Jankowska EA, van Veldhuisen DJ, Ponikowski P, Anker SD. Iron deficiency and cardiovascular disease. Nature Reviews Cardiology. 2015;12(11):659-69.
3. Darnton-Hill I, Webb P, Harvey PWJ, Hunt JM, Dalmiya N, Chopra M, et al. Micronutrient deficiencies and gender: social and economic costs. The American Journal of Clinical Nutrition. 2005;81(5):1198S-205S.
4. Lim SS, Vos T, Flaxman AD, Danaei G, Shibuya K, Adair-Rohani H, et al. A comparative risk assessment of burden of disease and injury attributable to 67 risk factors and risk factor clusters in 21 regions, 1990-2010: a systematic analysis for the Global Burden of Disease Study 2010. Lancet. 2012;380(9859):2224-60.
5. NHS Digital. Hospital Admitted Patient Care Activity 2020 [Available from: https://digital.nhs.uk/data-and-information/publications/statistical/hospital-admitted-patient-care-activity.
6. House of Commons Environmental Audit Committee. Sustainable Development Goals in the UK follow up: hunger, malnutrition and food insecurity in the UK. London: House of Commons; 2019.
7. HM Treasury. Budget 2010. 2010.
8. HM Treasury. Chancellor announces reforms to the welfare system 2010 [Available from: https://www.gov.uk/government/news/chancellor-announces-reforms-to-the-welfare-system.
9. Smith NA, Phillips D, Simpson P, Eiser D, Trickey M. A time of revolution? British local government finance in the 2010s. Institute for Fiscal Studies; 2016.
10. Centre for Cities. Cities Outlook 2019: A Decade of Austerity 2019.
11. Ministry of Housing Communities & Local Government. General Fund Revenue Account Outturn 2019-20 Specific guidance notes for completing the REVENUE OUTTURN (RO) suite of forms. 2020.
12. Bambra C, Gibson M, Sowden A, Wright K, Whitehead M, Petticrew M. Tackling the wider social determinants of health and health inequalities: evidence from systematic reviews. Journal of Epidemiology and Community Health. 2010;64(4):284.
13. Marmot M, Allen, J, Boyce, T, et al. Health Equity in England: The Marmot Review 10 Years On. 2020.
14. Alexiou A, Fahy K, Mason K, Bennett D, Brown H, Bambra C, et al. Local government funding and life expectancy in England: a longitudinal ecological study. The Lancet Public Health. 2021;6:e641-47.
15. Jenkins RH, Aliabadi S, Vamos EP, et al. The relationship between austerity and food insecurity in the UK: A systematic review. EClinicalMedicine. 2021;33:100781.
16. Humphries RH, P; Charles, A; Thorlby, R; Holder, H. Social care for older people: Home truths. The Kings Fund 2016. Report No.: 978 1 909029 66 8.
17. NHS Digital. Community Care Statistics, Social Services Activity, England, 2013-2014 2014 [Available from: https://digital.nhs.uk/data-and-information/publications/statistical/adult-social-care-activity-and-finance-report/community-care-statistics-social-services-activity-england-2013-14-final-release.
18. Penny J, Slay J. Surviving Austerity: Local Voices and Local Action in England's Poorest Neighbourhoods: New Economics Foundation; 2013 [Available from: https://www.barrowcadbury.org.uk/wp-content/uploads/2013/08/Surviving-Austerity.pdf.
19. Wright L, Vance L, Sudduth C, Epps JB. The Impact of a Home-Delivered Meal Program on Nutritional Risk, Dietary Intake, Food Security, Loneliness, and Social Well-Being. Journal of Nutrition in Gerontology and Geriatrics. 2015;34(2):218-27.
20. Dewar M, Dickinson A, Smeeton N. Tracking and treating malnutrition: a retrospective observational study of the nutritional status of vulnerable people accessing a meals-on-wheels (MOW) service. Prim Health Care Res Dev. 2020;21:e19-e.
21. National Audit Office. Financial Sustainability of Local Authorities 2018.

22. Pollack CE, Griffin BA, Lynch J. Housing Affordability and Health Among Homeowners and Renters. American Journal of Preventive Medicine. 2010;39(6):515-21.
23. Office for National Statistics. Family spending in the UK: April 2018 to March 2019 2020 [Available from: https://www.ons.gov.uk/peoplepopulationandcommunity/personalandhouseholdfinances/expenditure/bulletins/familyspendingintheuk/april2018tomarch2019.
24. Jones NRV, Conklin AI, Suhrcke M, Monsivais P. The Growing Price Gap between More and Less Healthy Foods: Analysis of a Novel Longitudinal UK Dataset. PLOS ONE. 2014;9(10):e109343.
25. Sheffield Political Economy Research Institute. Heating or eating and the impact of austerity. 2018.
26. Hastings A, Bailey N, Bramley G, Gannon M. Austerity urbanism in England: The 'regressive redistribution' of local government services and the impact on the poor and marginalised. Environment and Planning A: Economy and Space. 2017;49(9):2007-24.
27. Gundlach L, Resch B, Omri M. Food Deserts: Evaluating Grocery Stores and Bus Accessibility using GIS in Madison, Wisconsin. 2013.
28. Rose D, Richards R. Food store access and household fruit and vegetable use among participants in the US Food Stamp Program. Public health nutrition. 2004;7(8):1081-8.
29. Lopresti AL. The Effects of Psychological and Environmental Stress on Micronutrient Concentrations in the Body: A Review of the Evidence. Adv Nutr. 2020;11(1):103-12.
30. Barr B, Alexiou A. Local Authority Finance: Net Current Expenditure - Highways and Transport services (FIN_07_04), Verson 1.01, Place-Based Longitudinal Data Resource [Internet]; 2021. Podcast
31. Barr B, Alexiou A. Local Authority Finance: Net Current Expenditure - Social Care services (FIN_07_05), Verson 1.01, Place-Based Longitudinal Data Resource [Internet]; 2021. Podcast
32. Barr B, Alexiou A. Local Authority Finance: Net Current Expenditure - Housing services (GFRA only) (FIN_07_09), Verson 1.01, Place-Based Longitudinal Data Resource [Internet]; 2021. Podcast
33. Barr B, Alexiou A. Local Authority Finance: Net Current Service Expenditure - Cultural and related services (FIN_07_10), Verson 1.01, Place-Based Longitudinal Data Resource [Internet]; 2021. Podcast
34. Barr B, Alexiou A. Local Authority Finance: Net Current Expenditure - Environmental and regulatory services (FIN_07_11), Verson 1.01, Place-Based Longitudinal Data Resource [Internet]; 2021. Podcast
35. Barr B, Alexiou A. Local Authority Finance: Net Current Expenditure - Planning and development services (FIN_07_12), Verson 1.01, Place-Based Longitudinal Data Resource [Internet]; 2021. Podcast
36. Barr B, Alexiou A. Local Authority Finance: Net Current Expenditure - Central services (FIN_07_13), Verson 1.01, Place-Based Longitudinal Data Resource [Internet]; 2021. Podcast
37. Innes D, Tetlow G. Delivering Fiscal Squeeze by Cutting Local Government Spending. Fiscal Studies. 2015;36(3):303-25.
38. Office for National Statistics. CPIH ANNUAL RATE 00: ALL ITEMS 2015=100 2021 [Available from: https://www.ons.gov.uk/economy/inflationandpriceindices/timeseries/l55o/mm23.
39. NHS Digital. Hospital Episode Statistics (HES) 2021 [Available from: https://digital.nhs.uk/data-and-information/data-tools-and-services/data-services/hospital-episode-statistics.
40. Barr B, Alexiou A. Total primary diagnoses of nutritional anaemias (D50-53) excluding primary or secondary diagnosis of D50.0, version 3 [Safeguarded Dataset], Place-Based Longitudinal Data Resource [Internet]; 2021. Podcast
41. Barr B, Alexiou A. Total secondary diagnoses of nutritional anaemias (D50-53) excluding primary or secondary diagnosis of D50.0, version 3 [Safeguarded Dataset], Place-Based Longitudinal Data Resource [Internet]; 2021. Podcast
42. Barr B, Alexiou A. Total primary diagnoses of nutritional anaemias (D50-53) excluding primary or secondary diagnosis of D50.0, C00-C96, F50, R13, K91.2, R64, E70-90, K50.90, K51.90,

For peer review only

K63.2,Z99.2, N17-19,K70-77,E08-E11 and O24.4, version 3 [Safeguarded Dataset], Place-Based Longitudinal Data Resource [Internet]; 2021. Podcast
43. Barr B, Alexiou A. Total secondary diagnoses of nutritional anaemias (D50-53) excluding primary or secondary diagnosis of D50.0,C00-C96, F50, R13, K91.2, R64, E70-90, K50.90, K51.90, K63.2, Z99.2, N17-19, K70-77, E08-E11 and O24.4, version 3 [Safeguarded Dataset], Place-Based Longitudinal Data Resource [Internet]; 2021. Podcast
44. U.S. Federal Government's Department of Health and Human Services. ICD-10-CM Official Guidelines for Coding and Reporting. 2020.
45. Office for National Statistics. Lower layer Super Output Area population estimates 2020 [Available from: https://www.ons.gov.uk/peoplepopulationandcommunity/populationandmigration/populationestimates/datasets/lowersuperoutputareamidyearpopulationestimates.
46. Office for National Statistics. Regional gross disposable household income QMI 2018 [Available from: https://www.ons.gov.uk/economy/regionalaccounts/grossdisposablehouseholdincome/methodologies/regionalgrossdisposablehouseholdincomeqmi.
47. Office for National Statistics. Model-Based Estimates of ILO Unemployment for LAD/UAs in Great Britain: Guide for Users. 2006.
48. Ministry of Housing Communities & Local Government. English indices of deprivation 2015 2015 [Available from: https://www.gov.uk/government/statistics/english-indices-of-deprivation-2015].
49. Beatty C, Fothergill, S. The uneven impact of welfare reform: the financial losses to places and people. Sheffield Hallam University Centre for Regional Economic and Social Research; 2016.
50. Cherrie M, Curtis S, Baranyi G, Cunningham N, Dibben C, Bambra C, et al. A data linkage study of the effects of the Great Recession and austerity on antidepressant prescription usage. European Journal of Public Health. 2021;31(2):297-303.
51. Wooldridge JM. Econometric analysis of cross section and panel data: MIT press; 2010.
52. Wooldridge JM. Distribution-free estimation of some nonlinear panel data models. Journal of Econometrics. 1999;90(1):77-97.
53. Bertrand M, Duflo E, Mullainathan S. How Much Should We Trust Differences-In-Differences Estimates? The Quarterly Journal of Economics. 2004;119(1):249-75.
54. Elia M. The cost of malnutrition in England and potential cost savings from nutritional interventions (short version): A report on the cost of disease-related malnutrition in England and a budget impact analysis of implementing the NICE clinical guidelines/quality standard on nutritional support in adults. BAPEN; 2015.
55. Public Health England. National Diet and Nutrition Survey. Years 1 to 9 of the Rolling Programme (2008/2009 – 2016/2017): Time trend and income analyses. 2019.
56. Public Health England. National Diet and Nutrition Survey. Rolling programme Years 9 to 11 (2016/2017 to 2018/2019). 2020.
57. Russell CA, Elia M. NUTRITION SCREENING SURVEYS IN HOSPITALS IN THE UK, 2007-2011. BAPEN 2014.
58. Prentice A. Sex differences in requirements for micronutrients across the lifecourse. Proceedings of the Nutrition Society. 2021;80(3):356-64.
59. Alston P. Statement on Visit to the United Kingdom, by Professor Philip Alston, United Nations Special Rapporteur on extreme poverty and human rights 2018 [Available from: https://www.ohchr.org/EN/NewsEvents/Pages/DisplayNews.aspx?NewsID=23881&LangID=E.
60. Watkins J, Wulaningsih W, Da Zhou C, Marshall DC, Sylianteng GDC, Dela Rosa PG, et al. Effects of health and social care spending constraints on mortality in England: a time trend analysis. BMJ Open. 2017;7(11):e017722.
61. McLean G, Gunn J, Wyke S, Guthrie B, Watt GC, Blane DN, et al. The influence of socioeconomic deprivation on multimorbidity at different ages: a cross-sectional study. British Journal of General Practice. 2014;64(624):e440-e7.
62. The Rt Hon Boris Johnson MP. The Prime Minister's Levelling Up speech: 15 July 2021 2021 [Available from: https://www.gov.uk/government/speeches/the-prime-ministers-levelling-up-speech-15-july-2021.

For peer review only

63. Robinson T, Brown H, Norman PD, Fraser LK, Barr B, Bambra C. The impact of New Labour's English health inequalities strategy on geographical inequalities in infant mortality: a time-trend analysis. Journal of Epidemiology and Community Health. 2019;73(6):564-8.
64. Dixon J, Everest G. Briefing: The government's levelling up agenda The Health Foundation; 2021 [Available from: https://reader.health.org.uk/governments-levelling-up-agenda.

For peer review only

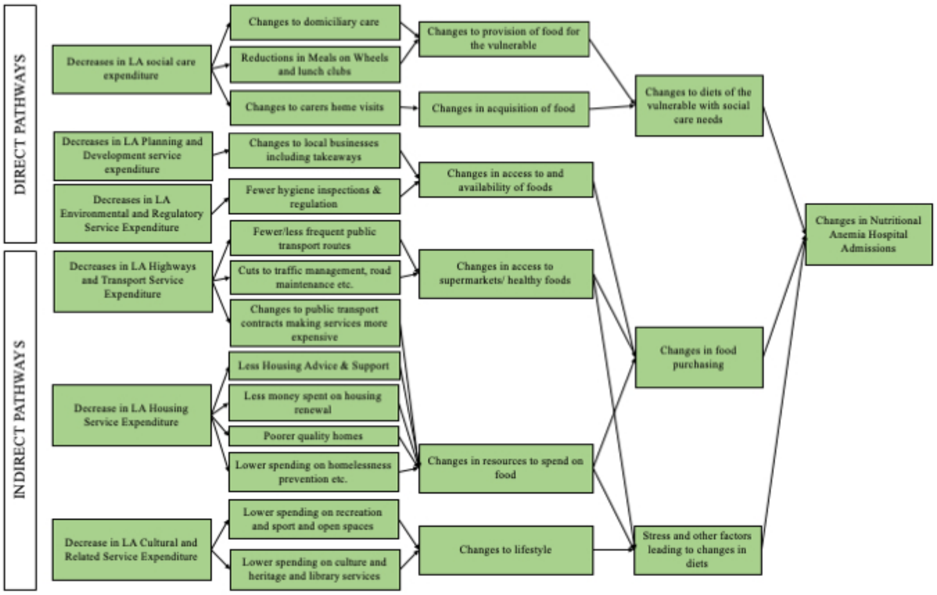

Figure 1: Logic model of potential pathways through which changes to LA spending may affect nutritional anaemias

397x336mm (46 x 46 DPI)

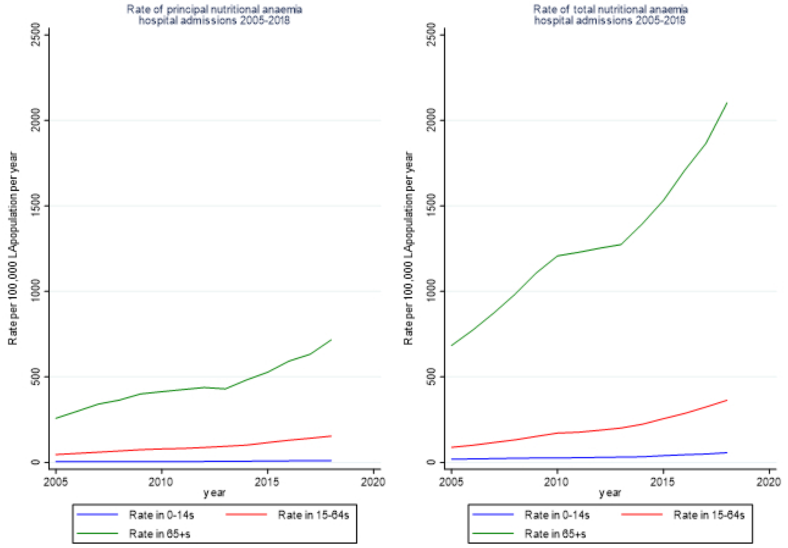

Figure 2: Rates of principal and total nutritional anaemia hospital admissions by year, stratified by age.

216x128mm (96 x 96 DPI)

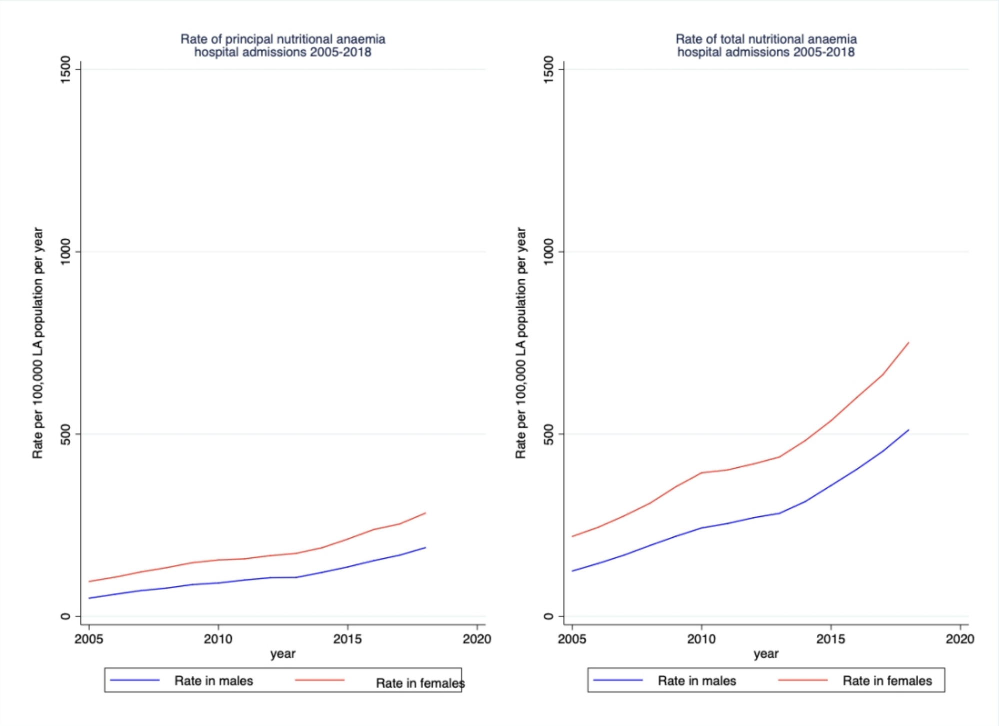

Figure 3: Rates of principal and total nutritional anaemia hospital admissions by year, stratified by sex

219x159mm (144 x 144 DPI)

Appendix 1: ICD-10 codes for conditions included in this study

**ICD-10 code**	**Diagnosis**
Principal and total nutritional anaemia hospital admissions:	
D50	Iron deficiency anaemia
D51	Vitamin B12 deficiency anaemia
D52	Folate deficiency anaemia
D53	Other nutritional anaemias
Excluding admissions alongside a principal or secondary diagnosis of:	
D50.0	Iron deficiency anaemia secondary to blood loss
C00-C96	Malignancies
F50	Eating disorders
R13	Difficulty swallowing
K91.2	Postoperative malabsorption
R64	Cachexia
E70-90	Metabolic disorders
K50.90, K51.90	GI disease including Crohn's disease and ulcerative colitis
K63.2	Fistula
Z99.2, N17-19	Kidney disease and dialysis
K70-77	Liver disease
E08-E11, O24.4	Diabetes mellitus (due to underlying condition, drug or chemical induced, type I, type II, gestational)

For peer review only

Appendix 2: Changes in mean total LA spending over time

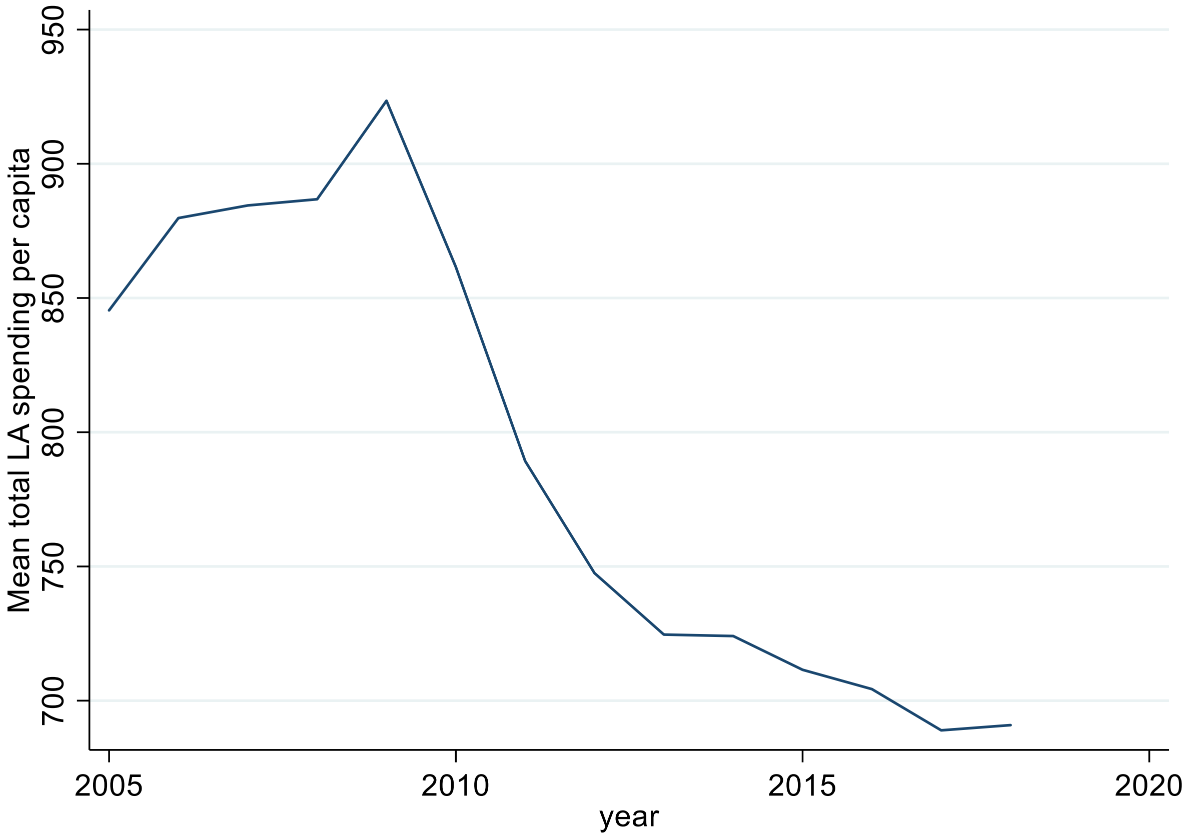

view only

Appendix 3: Impact of LA service spending on total nutritional anaemia hospital admissions, shown as the Incident Rate Ratio and stratified by gender (95% Confidence Intervals in brackets)

	Incidence Rate Ratio for total nutritional anaemia hospital admissions by LA with an £100 increase in total LA service spending		
	All Ages	Male	Female
Full Sample[1]	0.981 (0.964, 0.999) p=0.038	0.975 (0.957, 0.993) p=0.007	0.992 (0.974, 1.010) p=0.358
IMD[2]			
1 (most deprived)	0.974 (0.951, 0.998) p=0.032	0.968 (0.944, 0.993) p=0.012	0.980 (0.956, 1.005) p=0.118
2	0.991 (0.949, 1.035) p=0.678	0.988 (0.947, 1.030) p=0.563	1.005 (0.962, 1.050) p=0.818
3	1.016 (0.976, 1.058) p=0.431	1.009 (0.962, 1.058) p=0.707	1.032 (0.989, 1.076) p=0.151
4	0.883 (0.831, 0.939) p<0.001	0.872 (0.817, 0.932) p<0.001	0.902 (0.851, 0.956) p<0.001
5 (least deprived)	1.011 (0.955, 1.070) p=0.712	1.001 (0.936, 1.071) p=0.967	1.029 (0.967, 1.094) p=0.367
Level of reductions to working age benefits per capita			
1 (lowest reductions)	1.011 (0.961, 1.063) p=0.680	1.013 (0.962, 1.067) p=0.623	1.020 (0.962, 1.083) p=0.506
2	0.963 (0.935, 0.991) p=0.010	0.955 (0.926, 0.985) p=0.004	0.981 (0.951, 1.011) p=0.210
3	0.992 (0.952, 1.032) p=0.671	0.983 (0.944, 1.024) p=0.419	0.999 (0.960, 1.040) p=0.960
4 (greatest reductions)	0.975 (0.949, 1.003) p=0.077	0.970 (0.942, 1.000) p=0.048	0.986 (0.957, 1.015) p=0.330

[1] Adjusted by percentage working age, percentage male, GDHI and unemployment rate.

[2] Index of Multiple Deprivation, quintiles based on relative ranking of LAs

For peer review only

Appendix 4: Association between LA service spending and principal nutritional anaemia hospital admissions, shown as the Incident Rate Ratio and stratified by age (95% Confidence Intervals in brackets).

	Incidence Rate Ratio for principal nutritional anaemia hospital admissions by LA with an £100 increase in total LA service spending			
	All Ages	0-14	15-64	65+
Full sample[1]	0.987 (0.963, 1.011) p=0.293	0.996 (0.945, 1.051) p=0.896	0.986 (0.957, 1.015) p=0.332	0.983 (0.957, 1.009) p=0.190
IMD[2]				
1 (most deprived)	0.985 (0.950, 1.022) p=0.435	0.969 (0.899, 1.043) p=0.400	0.989 (0.945, 1.035) p=0.634	0.978 (0.941, 1.016) p=0.247
2	0.988 (0.942, 1.036) p=0.611	0.984 (0.866, 1.119) p=0.806	1.000 (0.950, 1.053) p=0.999	0.975 (0.928, 1.024) p=0.311
3	1.031 (0.973, 1.092) p=0.308	1.086 (0.895, 1.319) p=0.402	1.051 (0.989, 1.116) p=0.107	1.002 (0.929, 1.082) p=0.951
4	0.838 (0.759, 0.925) p<0.001	1.255 (1.009, 1.560) p=0.041	0.798 (0.715, 0.892) p<0.001	0.853 (0.769, 0.946) p=0.003
5 (least deprived)	1.040 (0.957, 1.130) p=0.357	1.135 (0.943, 1.365) p=0.180	1.013 (0.929, 1.104) p=0.778	1.053 (0.968, 1.146) p=0.226
Level of reductions to working age benefits per capita				
1 (lowest reductions)	1.041 (0.972, 1.115) p=0.252	1.205 (1.001, 1.451) p=0.049	1.019 (0.949, 1.095) p=0.601	1.052 (0.977, 1.132) p=0.180
2	0.967 (0.927, 1.009) p=0.125	1.002 (0.835, 1.203) p=0.983	0.959 (0.910, 1.010) p=0.113	0.968 (0.927, 1.012) p=0.148
3	1.000 (0.949, 1.054) p=0.995	1.030 (0.919, 1.153) p=0.616	1.016 (0.965, 1.070) p=0.548	0.978 (0.920, 1.040) p=0.479
4 (greatest reductions)	0.980 (0.947, 1.014) p=0.242	0.975 (0.916, 1.038) p=0.430	0.979 (0.937, 1.022) p=0.333	0.975 (0.943, 1.008) p=0.140

[1] Adjusted by percentage working age, percentage male, GDHI and unemployment rate.

[2] Index of Multiple Deprivation, quintiles based on relative ranking of LAs

For peer review only

Appendix 5: Rates of principal and total nutritional anaemia hospital admissions over time, excluding those alongside other conditions which may cause nutritional deficiencies, stratified by age (2005-2018)

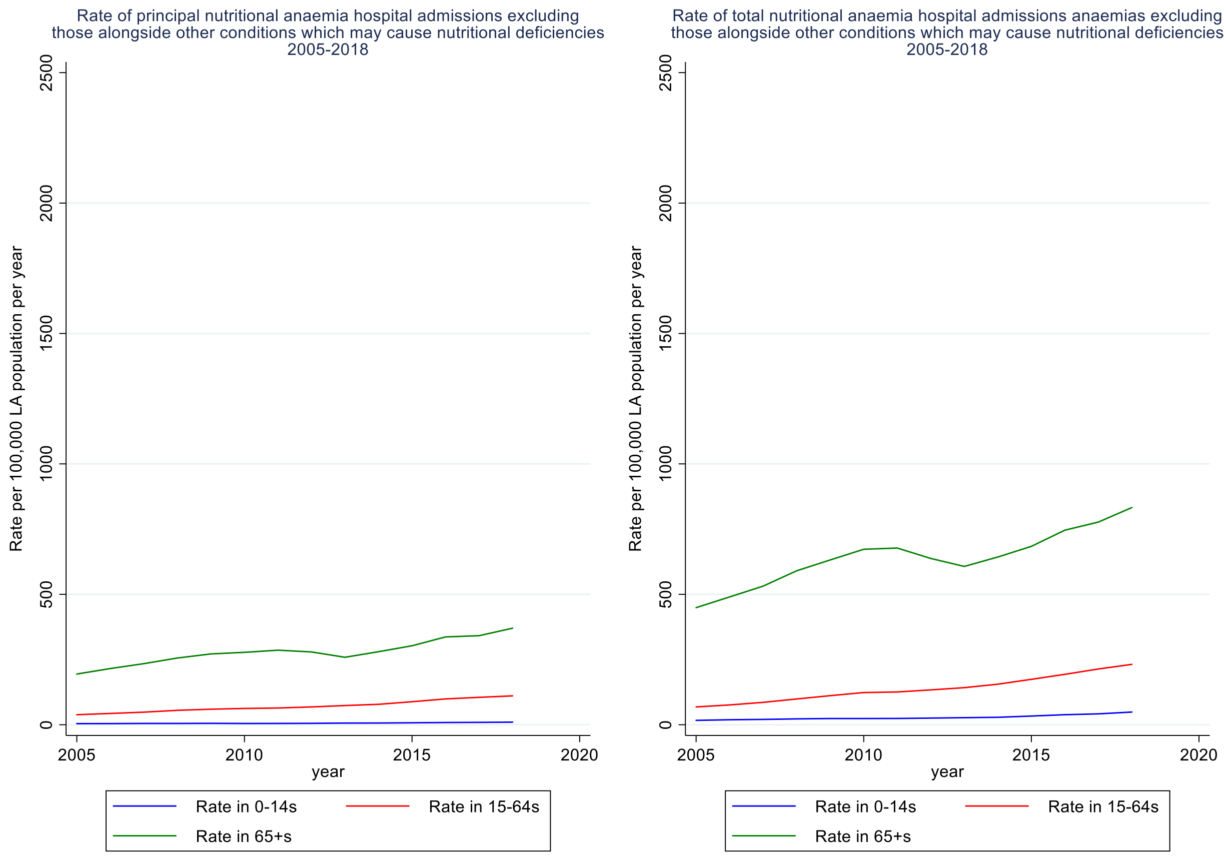

For peer review only

Appendix 6: Association between LA service spending and total nutritional anaemia hospital admissions excluding those alongside a principal or secondary diagnosis of another condition which may cause a nutritional deficiency, shown as the Incident Rate Ratio and stratified by age (95% Confidence Intervals in brackets)

	Incidence Rate Ratio for total nutritional anaemia hospital admissions by LA with an £100 increase in total LA service spending			
	All Ages	0-14	15-64	65+
Full sample[1]	0.991 (0.974, 1.009) p=0.312	0.967 (0.894, 1.047) p=0.408	0.989 (0.969, 1.009) p=0.279	0.998 (0.980, 1.017) p=0.829
IMD[2]				
1 (most deprived)	0.985 (0.962, 1.009) p=0.208	0.933 (0.823, 1.058) p=0.279	0.981 (0.952, 1.011) p=0.207	0.994 (0.969, 1.020) p=0.658
2	1.001 (0.961, 1.044) p=0.950	1.047 (0.989, 1.109) p=0.118	1.009 (0.967, 1.052) p=0.692	1.002 (0.955, 1.050) p=0.945
3	1.034 (0.998, 1.072) p=0.067	1.025 (0.934, 1.125) p=0.599	1.041 (0.991, 0.093) p=0.110	1.029 (0.992, 1.066) p=0.128
4	0.876 (0.818, 0.938) p<0.001	0.931 (0.789, 1.098) p=0.396	0.850 (0.786, 0.920) p<0.001	0.888 (0.828, 0.953) p=0.001
5 (least deprived)	1.030 (0.968, 1.095) p=0.357	0.977 (0.818, 1.167) p=0.799	1.024 (0.959, 1.094) p=0.472	1.038 (0.976, 1.104) p=0.237
Level of reductions to working age benefits per capita				
1 (lowest reductions)	1.026 (0.968, 1.087) p=0.390	0.979 (0.834, 1.149) p=0.792	1.031 (0.976, 1.088) p=0.279	1.027 (0.963, 1.096) p=0.412
2	0.976 (0.947, 1.005) p=0.104	0.973 (0.906, 1.045) p=0.446	0.960 (0.924, 0.997) p=0.035	0.993 (0.964, 1.023) p=0.656
3	1.004 (0.964, 1.047) p=0.839	1.030 (0.919, 1.154) p=0.616	1.012 (0.970, 1.055) p=0.590	1.002 (0.956, 1.050) p=0.946
4 (greatest reductions)	0.981 (0.956, 1.007) p=0.150	0.925 (0.813, 1.052) p=0.234	0.978 (0.946, 1.012) p=0.198	0.988 (0.963, 1.013) p=0.326

[1] Adjusted by percentage working age, percentage male, GDHI and unemployment rate.

[2] Index of Multiple Deprivation, quintiles based on relative ranking of LAs

For peer review only

Appendix 7: Association between LA service spending excluding social care spending and total nutritional anaemia hospital admissions, shown as the Incident Rate Ratio and stratified by age (95% Confidence Intervals in brackets)

	Incidence Rate Ratio for total nutritional anaemia hospital admissions by LA with an £100 increase in total LA service spending			
	All Ages	0-14	15-64	65+
Full Sample[1]	0.960 (0.939, 0.982) p<0.001	0.960 (0.869, 1.061) p=0.425	0.958 (0.934, 0.983) p=0.001	0.958 (0.936, 0.980) p<0.001
IMD[2]				
1 (most deprived)	0.957 (0.927, 0.987) p=0.006	0.903 (0.769, 1.060) p=0.213	0.950 (0.916, 0.986) p=0.007	0.962 (0.931, 0.995) p=0.025
2	0.976 (0.935, 1.020) p=0.278	1.074 (1.020, 1.131) p=0.006	0.989 (0.944, 1.037) p=0.652	0.962 (0.919, 1.008) p=0.104
3	0.988 (0.934, 1.045) p=0.670	1.087 (0.973, 1.214) p=0.141	0.990 (0.927, 1.056) p=0.750	0.973 (0.919, 1.030) p=0.349
4	0.847 (0.796, 0.901) p<0.001	0.959 (0.803, 1.146) p=0.645	0.822 (0.760, 0.888) p<0.001	0.849 (0.799, 0.901) p<0.001
5 (least deprived)	1.003 (0.931, 1.081) p=0.934	1.031 (0.889, 1.195) p=0.687	1.005 (0.924, 1.092) p=0.916	0.997 (0.933, 1.065) p=0.934
Level of reductions to working age benefits per capita				
1 (lowest reductions)	0.982 (0.914, 1.055) p=0.612	0.997 (0.845, 1.177) p=0.974	0.984 (0.910, 1.065) p=0.695	0.975 (0.912, 1.042) p=0.457
2	0.940 (0.903, 0.979) p=0.003	0.957 (0.874, 1.047) p=0.339	0.936 (0.891, 0.982) p=0.007	0.937 (0.900, 0.975) p=0.001
3	0.961 (0.922, 1.002) p=0.062	1.025 (0.912, 1.152) p=0.685	0.966 (0.924, 1.009) p=0.123	0.951 (0.909, 0.994) p=0.027
4 (greatest reductions)	0.963 (0.930, 0.997) p=0.031	0.911 (0.774, 1.072) p=0.261	0.959 (0.921, 0.999) p=0.043	0.965 (0.932, 1.000) p=0.047

[1] Adjusted by percentage working age, percentage male, GDHI and unemployment rate.
[2] Index of Multiple Deprivation, quintiles based on relative ranking of LAs

For peer review only

STROBE Statement—checklist of items that should be included in reports of observational studies

	Item No	Recommendation	Page No
**Title and abstract**	1	(*a*) Indicate the study's design with a commonly used term in the title or the abstract	1
		(*b*) Provide in the abstract an informative and balanced summary of what was done and what was found	1
**Introduction**			
Background/rationale	2	Explain the scientific background and rationale for the investigation being reported	2,3
Objectives	3	State specific objectives, including any prespecified hypotheses	3
**Methods**			
Study design	4	Present key elements of study design early in the paper	3
Setting	5	Describe the setting, locations, and relevant dates, including periods of recruitment, exposure, follow-up, and data collection	3,4
Participants	6	(*a*) *Cohort study*—Give the eligibility criteria, and the sources and methods of selection of participants. Describe methods of follow-up *Case-control study*—Give the eligibility criteria, and the sources and methods of case ascertainment and control selection. Give the rationale for the choice of cases and controls *Cross-sectional study*—Give the eligibility criteria, and the sources and methods of selection of participants	4
		(*b*) *Cohort study*—For matched studies, give matching criteria and number of exposed and unexposed *Case-control study*—For matched studies, give matching criteria and the number of controls per case	
Variables	7	Clearly define all outcomes, exposures, predictors, potential confounders, and effect modifiers. Give diagnostic criteria, if applicable	5
Data sources/ measurement	8*	For each variable of interest, give sources of data and details of methods of assessment (measurement). Describe comparability of assessment methods if there is more than one group	*5*
Bias	9	Describe any efforts to address potential sources of bias	5,6
Study size	10	Explain how the study size was arrived at	6
Quantitative variables	11	Explain how quantitative variables were handled in the analyses. If applicable, describe which groupings were chosen and why	6
Statistical methods	12	(*a*) Describe all statistical methods, including those used to control for confounding	6,7
		(*b*) Describe any methods used to examine subgroups and interactions	7
		(*c*) Explain how missing data were addressed	7
		(*d*) *Cohort study*—If applicable, explain how loss to follow-up was addressed *Case-control study*—If applicable, explain how matching of cases and controls was addressed *Cross-sectional study*—If applicable, describe analytical methods taking account of sampling strategy	7
		(*e*) Describe any sensitivity analyses	

Continued on next page

For peer review only - http://bmjopen.bmj.com/site/about/guidelines.xhtml

**Results**			
Participants	13*	(a) Report numbers of individuals at each stage of study—eg numbers potentially eligible, examined for eligibility, confirmed eligible, included in the study, completing follow-up, and analysed	7
		(b) Give reasons for non-participation at each stage	7
		(c) Consider use of a flow diagram	7
Descriptive data	14*	(a) Give characteristics of study participants (eg demographic, clinical, social) and information on exposures and potential confounders	Table 1
		(b) Indicate number of participants with missing data for each variable of interest	Table 1
		(c) *Cohort study*—Summarise follow-up time (eg, average and total amount)	
Outcome data	15*	*Cohort study*—Report numbers of outcome events or summary measures over time	Table 1
		Case-control study—Report numbers in each exposure category, or summary measures of exposure	
		Cross-sectional study—Report numbers of outcome events or summary measures	
Main results	16	(*a*) Give unadjusted estimates and, if applicable, confounder-adjusted estimates and their precision (eg, 95% confidence interval). Make clear which confounders were adjusted for and why they were included	8
		(*b*) Report category boundaries when continuous variables were categorized	8
		(*c*) If relevant, consider translating estimates of relative risk into absolute risk for a meaningful time period	8
Other analyses	17	Report other analyses done—eg analyses of subgroups and interactions, and sensitivity analyses	8,9
**Discussion**			
Key results	18	Summarise key results with reference to study objectives	9
Limitations	19	Discuss limitations of the study, taking into account sources of potential bias or imprecision. Discuss both direction and magnitude of any potential bias	10
Interpretation	20	Give a cautious overall interpretation of results considering objectives, limitations, multiplicity of analyses, results from similar studies, and other relevant evidence	10,11
Generalisability	21	Discuss the generalisability (external validity) of the study results	11
**Other information**			
Funding	22	Give the source of funding and the role of the funders for the present study and, if applicable, for the original study on which the present article is based	13

*Give information separately for cases and controls in case-control studies and, if applicable, for exposed and unexposed groups in cohort and cross-sectional studies.

Note: An Explanation and Elaboration article discusses each checklist item and gives methodological background and published examples of transparent reporting. The STROBE checklist is best used in conjunction with this article (freely available on the Web sites of PLoS Medicine at http://www.plosmedicine.org/, Annals of Internal Medicine at http://www.annals.org/, and Epidemiology at http://www.epidem.com/). Information on the STROBE Initiative is available at www.strobe-statement.org.

For peer review only - http://bmjopen.bmj.com/site/about/guidelines.xhtml

Local area public sector spending and nutritional anaemia hospital admissions in England: a longitudinal ecological study

Journal:	*BMJ Open*
Manuscript ID	bmjopen-2021-059739.R2
Article Type:	Original research
Date Submitted by the Author:	22-Aug-2022
Complete List of Authors:	Jenkins, Rosemary; Imperial College London<br>Vamos, Eszter; Imperial College London, School of Public Health<br>Mason, Kate; University of Liverpool, Public Health & Policy<br>Daras, Konstantinos ; University of Liverpool, Public Health, Policy and Systems<br>Taylor-Robinson, David; University of Liverpool,<br>Bambra, Clare; Newcastle University<br>Millett, Christopher; Imperial College, Primary Care and Social Medicine<br>Laverty, Anthony; Imperial College London, Primary Care and Public Health
**Primary Subject Heading**:	Public health
Secondary Subject Heading:	Health policy
Keywords:	SOCIAL MEDICINE, PUBLIC HEALTH, Health policy < HEALTH SERVICES ADMINISTRATION & MANAGEMENT

SCHOLARONE™
Manuscripts

I, the Submitting Author has the right to grant and does grant on behalf of all authors of the Work (as defined in the below author licence), an exclusive licence and/or a non-exclusive licence for contributions from authors who are: i) UK Crown employees; ii) where BMJ has agreed a CC-BY licence shall apply, and/or iii) in accordance with the terms applicable for US Federal Government officers or employees acting as part of their official duties; on a worldwide, perpetual, irrevocable, royalty-free basis to BMJ Publishing Group Ltd ("BMJ") its licensees and where the relevant Journal is co-owned by BMJ to the co-owners of the Journal, to publish the Work in this journal and any other BMJ products and to exploit all rights, as set out in our licence.

The Submitting Author accepts and understands that any supply made under these terms is made by BMJ to the Submitting Author unless you are acting as an employee on behalf of your employer or a postgraduate student of an affiliated institution which is paying any applicable article publishing charge ("APC") for Open Access articles. Where the Submitting Author wishes to make the Work available on an Open Access basis (and intends to pay the relevant APC), the terms of reuse of such Open Access shall be governed by a Creative Commons licence – details of these licences and which Creative Commons licence will apply to this Work are set out in our licence referred to above.

Other than as permitted in any relevant BMJ Author's Self Archiving Policies, I confirm this Work has not been accepted for publication elsewhere, is not being considered for publication elsewhere and does not duplicate material already published. I confirm all authors consent to publication of this Work and authorise the granting of this licence.

For peer review only

Local area public sector spending and nutritional anaemia hospital admissions in England: a longitudinal ecological study

Rosemary H. Jenkins[1], Eszter P Vamos[1], Kate E. Mason[2], Konstantinos Daras[2], David Taylor-Robinson[2], Clare Bambra[3], Christopher Millett[1], Anthony A. Laverty[1]

[1] Department of Primary Care and Public Health, School of Public Health, Imperial College London, Charing Cross Campus; The Reynolds Building; St Dunstan's Road; London, UK; W6 8RP.

[2] Department of Public Health, Policy and Systems, Institute of Population Health, University of Liverpool; Waterhouse Building Block F, 2nd Floor, Liverpool, UK; L69 3BX

[3] Population Health Sciences Institute, Faculty of Medical Sciences, Newcastle University, UK, NE1 4LE

Correspondence to: Dr Anthony Laverty, Public Health Policy Evaluation Unit, Department of Primary Care and Public Health, School of Public Health, Imperial College London, Charing Cross Campus; The Reynolds Building; St Dunstan's Road; London, UK; W6 8RP. a.laverty@imperial.ac.uk

For peer review only

ABSTRACT

Introduction: Reductions in local government spending may have impacts on diets and health which increase the risk of hospital admissions for nutritional anaemias. Mechanisms include potential impacts of changes to local authority (LA) services (e.g. housing services) on personal resources and food access, availability, and provision. We therefore investigated the association between changes in LA spending and nutritional anaemia related hospital admissions. Specifically we address whether greater cuts to LA spending were linked to increased hospital admissions for nutritional anaemia.

Design: Longitudinal analysis of LA panel data using Poisson fixed effects regression models.

Setting: 312 LAs in England (2005-2018).

Main exposure: Total LA service expenditure per capita per year.

Main outcome: Principal and total nutritional anaemia hospital admissions, for all ages and stratified by age (0-14, 15-64, 65+).

Results: LA service expenditure increased by 9% between 2005 and 2009 then decreased by 20% between 2010 and 2018. Total nutritional anaemia hospital admissions increased between 2005 and 2018 from 173 to 633 admissions per 100,000 population. A £100 higher LA service spending was associated with a 1.9% decrease in total nutritional anaemia hospital admissions (adjusted Incidence Rate Ratio: 0.98, 95% CI 0.96 to 0.99). When stratified by age, this was seen only in adults. A £100 higher LA service spending was associated with a 2.6% decrease in total nutritional anaemia hospital admissions in the most deprived LAs (aIRR: 0.97, 95% CI: 0.95 to 1.0).

Conclusion: Increased LA spending was associated with reduced hospital admissions for nutritional anaemia. Austerity related reductions had the opposite effect, increasing admissions, with greater impacts in more deprived areas. This adds further evidence to the potential negative impacts of austerity policies on health and health inequalities. Re-investing in LA services may prevent hospital admissions associated with nutritional anaemias.

Strengths and limitations of this study

- This study uses national data to assess relationships between public sector spending and hospital admissions
- A fixed effect panel design allows us to examine within local authority changes in both exposures and outcomes
- However, we examined hospitalisations only, which does not give a full picture of burden of disease, as measures such as GP diagnoses would
- The natural experimental design means that we cannot infer causality and research at the individual level would assist with this in the future

For peer review only

INTRODUCTION

Nutritional anaemias related to poor diet are an important public health issue, increasing in prevalence over recent years. Nutritional anaemias involve reductions in red blood cells or their haemoglobin content due to a low nutritional intake of certain micronutrients, particularly iron, vitamin B-12, and folate(1). Nutritional anaemias are associated with a range of adverse health outcomes – for example, iron deficiency anaemia can lead to poor outcomes in those with coronary heart disease(2). Furthermore, nutrient deficiencies, which cause nutritional anaemias, are important risk factors for diseases through multiple physiological mechanisms(3, 4). For example, hospitalisations for iron deficiency anaemias increased from 67,592 in 2008-09 to 131,064 in 2016-17(5). A 2019 report by the UK House of Commons Environmental Audit Committee described the presence of a double burden of malnutrition in the UK, with overweight and obesity coexisting with undernutrition, both leading to micronutrient deficiencies(6).

The increase in hospital admissions for nutritional anaemias has occurred at the same time as large cuts to public services in the UK(5). In 2010, the UK government introduced austerity policies which led to considerable changes to the UK benefits system and reductions in local authority (LA) funding and service expenditure(7, 8). Reductions in LA funding had greater impacts in urban and more deprived councils, especially in the north of England, leading to differential impacts on the wide range of services that LAs provide(9-11). These services include social care, housing, highways and transport, environment and regulatory, and planning and development services(11). Systematic review evidence demonstrates that these services may be important in improving the social determinants of health and health inequalities(12). Research reports have found that reductions to LA service spending may have had negative impacts on health and widened health inequalities(13). For example, a £100 decrease in annual LA funding per person has been associated with a decrease in life expectancy at birth of 1.2-1.3 months between 2013-17(14).

The relationship between changes in local government spending and risk of nutritional anaemia is unclear. Much of the extant research on nutritional impacts of austerity policies in the UK has focused on foodbank use and not other potential outcomes such as hospital admissions(15). Risk of nutritional anaemias caused by poor diets may be plausibly impacted by changes to public sector spending through a range of different mechanisms. Decreases in adult social care expenditure, particularly in more deprived LAs, may have led to fewer residential and domiciliary care placements, which could have precipitated changes in food provision to vulnerable people if they are not able to access the food provided through such care placements(16). Decreases in Meals on Wheels services, lunch clubs, and other food provision may also have a similar effect(17, 18). These

For peer review only

services have been shown to be important in providing vulnerable adults with the calories and nutrients that they need, preventing malnutrition(19, 20). Thus, decreases in these services may directly lead to increases in nutritional anaemias in these individuals due to deficits in nutrients. Fewer carers visiting homes may also lead to issues with food acquisition as individuals may rely on carers for shopping, which may also lead to changes in food intakes and potential nutritional anaemias. Changes to LA environmental and regulatory spending and planning and development spending may influence access and availability of foods through changes to local businesses, hygiene inspections, and food regulation, which may also lead to changes in food purchasing, particularly takeaways(21).

There are also pathways which may indirectly lead to reductions in micronutrient consumption and nutritional anaemias. Through factors including less housing advice and support, less money spent on housing renewal, poorer quality homes and lower spending on homelessness, reductions in LA housing service spending may lead to trade-offs between housing costs and paying for other items, including healthy food, due to a reduction in personal resources(22, 23). As micronutrient-rich foods such as fruits and vegetables tend to be more expensive than ultra-processed foods which are generally low in micronutrients, these changes in resources may indirectly lead to nutritional anaemias through changes in types of foods purchased and consumed(24). Lower LA highways and transport spending may have similar impacts on personal resources and therefore food purchasing due to increased costs of public transport(11, 25, 26). Decreases in highways and transport spending may also lead to reductions in public transport (eg. number of different routes, number of busses per day etc.)(11, 25, 26). This may adversely affect individuals' access to supermarkets, potentially leading to decreases in nutritious foods, increases in ultra-processed foods, and increases in takeaways(27, 28). Finally, a decrease in money spent on cultural services (including recreation and sport, library services, culture and heritage and open spaces) may indirectly lead to micronutrient depletion through changes to lifestyle (such as physical activity) and potential increases in stress and mental health issues, though further research is needed in this area(21, 29). Thus, there are a number of potential pathways which may influence diagnoses of nutritional anaemias (Fig. 1). We therefore aimed to investigate the association between changes to LA service spending and principal and total nutritional anaemia hospital admissions in LAs in England. Given the role of LAs in addressing the social determinants of health, we hypothesise that effects may be stronger in more deprived LAs and those experiencing greater reductions in working age benefits.

METHODS

Data

For peer review only

Exposure variables

The time period for our analysis was 2005-2018. Annual LA service expenditure is reported by the Ministry of Housing, Communities & Local Government, compiled and freely available from the Place-based Longitudinal Data Resource (PLDR) for 326 LAs(30-36). These data are based on financial years rather than calendar years. We used net expenditure data for lower tier LAs, which excludes income from services. The Islands of Scilly and the City of London were not included due to small populations, and an additional 12 LAs were excluded due to missing covariate data. We calculated total net LA expenditure excluding police services and fire and rescue services (as these are funded by separate grants and precepts and provided by separate authorities), education services (due to changes in schooling provision over the study period), public health services (due to data not being available for all years due to changes in LA responsibilities) and court services (due to data not being available for all years)(37). All data were adjusted for inflation using the Consumer Price Index with 2015 as the reference year(38).

Outcome variables

Hospital Episode Statistics (HES) is a database of all NHS hospitals' admissions, A&E attendances, and outpatient appointments in England(39). For this study, we used yearly LA-level counts of inpatient or day-case admissions of nutritional anaemias in NHS hospitals available as safeguarded datasets from the PLDR(40-43). These were iron deficiency anaemia (excluding iron deficiency anaemia due to blood loss), vitamin B-12 deficiency anaemia, folate deficiency anaemia and other nutritional anaemias by LA. We obtained principal nutritional anaemia hospital admissions data where the nutritional anaemia was the "condition established after study to be chiefly responsible for occasioning the admission of the patient to the hospital for care"(44). We also obtained data for hospital admissions where nutritional anaemias are a secondary diagnosis, an additional condition that an individual may have during a hospital admission for a different principal diagnosis. These were given by each age (0-14, 15-64, 65+) or sex group individually. We aggregated these data to obtain principal nutritional anaemia hospital admissions and total nutritional anaemia hospital admissions (consisting of both principal and secondary diagnoses). In this paper, we focus on total nutritional anaemia hospital admissions as they key outcome for a number of reasons, including 1) principal nutritional anaemia hospital admissions are relatively uncommon, and thus total nutritional anaemia hospital admissions are more reflective of the burden of nutritional anaemias in the population and 2) the multisystem impact of nutritional anaemias means that the primary diagnoses may be considered to be a manifestation of nutritional anaemias as they led to the patient being ill

For peer review only

enough to seek medical care. We used annual LA population estimates from the Office for National Statistics (ONS) to generate admission rates per 100,000 population(45).

For sensitivity analyses, we also obtained counts of nutritional anaemia admissions excluding those alongside a principal or secondary diagnosis of another condition that may cause nutritional deficiencies, such as cachexia, malabsorption, malignancies and diabetes (full list in Appendix 1). As with the main outcome variable above, these data were obtained stratified by age or sex. These data were also aggregated to obtain principal and total (ie. principal and secondary) nutritional anaemia hospital admissions.

Covariates & Effect Modifiers

In the regression models we adjusted for the proportion of the LA population that is working age, proportion of LA population that is male, Gross Disposable Household Income (GDHI) and unemployment rate. The proportion of LA population that was working age (aged 15-64) and male were calculated from ONS data(45). These covariates were included to account for differences in changing LA demographics. We obtained GDHI data, an area-level measure of individuals' available money for spending or saving following payment of taxes and receipt of benefits, adjusted for inflation, from the ONS(46). LA unemployment rates were also obtained from the ONS, who calculate them based on a model utilising estimates from the Labour Force Survey and counts of people claiming unemployment benefits(47). GDHI and unemployment rate were used as markers of available household resources at the LA level. Time invariant factors were not adjusted for as they were removed by the fixed effects model, however, we also obtained Index of Multiple Deprivation (IMD) and level of reductions in working-age benefits as they were identified a priori as potential effect modifiers. IMD 2015 was obtained from the Ministry of Housing, Communities & Local Government(48). We used the population-weighted rank of average rank of LA IMD to make quintiles of relative deprivation. We also used the Uneven Impact of Welfare Reform dataset (provided by its authors) which estimated the cumulative reductions in working-age people's benefits for each LA due to welfare reforms since 2010(49). Previous research has found strong associations between reductions in welfare benefits and clinical outcomes(50). We derived quartiles of this variable to assess level of reductions to working-age benefits as a potential effect modifier.

Analyses

We descriptively examined total LA service expenditure in 2005, 2010, and 2018, including differences by covariates and proposed effect modifiers. We also calculated percentage changes before (2005-2009) and after (2010-2018) the introduction of austerity measures. We tabulated

For peer review only - http://bmjopen.bmj.com/site/about/guidelines.xhtml

mean principal and total nutritional anaemia hospital admissions for the full sample and stratified by age group, presented as rates per 100,000 population, and plotted their change over time. We also plotted change over time stratified by sex.

We used a Poisson fixed effects panel regression approach for the main analysis. Panel regression models can be used to investigate LA units over time while taking data clustering over time into account(51). Poisson fixed effects regression was used as a robust approach to modelling count panel data(52). We used cluster robust standard errors to adjust for potential autocorrelation and heteroskedasticity(53). We also created dummy variables for each year and used them to account for England-wide time effects. LA population was used as an offset variable to account for population size. For our primary analysis, we undertook a Poisson fixed effects analysis to investigate the relationship between total LA service spending and total nutritional anaemia hospital admissions. We present adjusted models (with covariates as proportion of the LA population that is working age, proportion of the LA population that is male, Gross Disposable Household Income (GDHI) and unemployment rate). We also subsequently stratified by IMD quintiles and level of benefit reductions quartiles to test a priori hypotheses regarding effect modification. We undertook analyses stratified by age and sex. We separately analysed principal nutritional anaemia hospital admissions as the outcome, with adjusted and stratified Poisson models as above.

We also undertook two sensitivity analyses. In the first, we used as the outcome variable total nutritional anaemia hospital admissions excluding admissions alongside a diagnosis of another condition which may cause a nutritional deficiency. While public sector spending reductions could cause or exacerbate this wider set of conditions, this sensitivity analysis focuses on diagnoses of being admitted *for* nutritional anaemia rather than *with* it. In the second sensitivity analysis, we excluded social care expenditure from the exposure variable. Unlike most areas of LA service expenditure, some LAs had to increase their social care expenditure after 2010 due to demand. Thus, LAs with higher spending on social care may have higher levels of nutritional anaemias, which may lead to issues of reverse causality when social care is included in the exposure variable(54). Our sensitivity analysis investigated the relationship between LA spending excluding social care and total nutritional anaemia hospital admissions.

Patient and Public Involvement

Plans for this research were presented to a committee of three public experts in November 2020. The public experts provided feedback on the research plans and appropriate changes were made following the meeting.

For peer review only

RESULTS

On average, LA service spending increased by 9% between 2005 and 2009, and then decreased by 20% between 2010 and 2018 (Table 1). Mean total LA spending per year is plotted in Appendix 2. Between 2005 and 2009, LAs in Yorkshire and the Humber (16%), the East Midlands (15%), and the North West (14%) increased expenditure the most, while London (4%) and the South East (5%) increased expenditure the least. However, LAs with the highest unemployment rates decreased expenditure between 2005-2009. Following 2010, London had the greatest decrease of 31%, followed by the Northern regions (a 27% decrease for the North East and 21% for both the North West and Yorkshire and the Humber). More deprived LAs experienced greater cuts (28% in the most deprived quintile compared to 12% for the least deprived quintile). Greater reductions were also seen in areas with higher unemployment rates and larger reductions to working age benefits.

Total nutritional anaemia hospital admissions have increased since 2005, from 173 per 100,000 population in 2005 to 632 per 100,000 population in 2018 (Fig. 2). Total nutritional anaemia hospital admissions in 65+ adults increased between 2005-2010, with the rate of increase slowing between 2010-2013, and then increasing more steeply between 2014 and 2018. Rate of admissions in 65+s was 684 per 100,000 population in 2005 and 2,103 per 100,000 in 2018. There was also an increase throughout the study period for 15-64-year olds, from 88 per 100,000 population in 2005 to 364 per 100,000 population. Total nutritional anaemia hospital admissions also increased slightly in 0-14s between 2005 and 2018, from 19 to 57 admissions per 100,000 population. Rates of admissions also increased for both men and women when stratified by sex, and women had a higher rate of nutritional anaemias than men (Fig. 3).

In 2018, the rate of principal nutritional anaemia hospital admissions was 236 per 100,000 population (Table 2). Rates increased with age (11 per 100,000 population for 0-14s, 154 for 15-64s, and 717 for 65+s). The rate of total nutritional anaemia hospital admissions in 2018 was 632 per 100,000 population. Rates of total nutritional anaemia hospital admissions also increased with age – rates were relatively low in children (57 per 100,000 population) but were 364 and 2,102 per 100,000 population in adults (15-64 and 65+ respectively). Principal and total nutritional anaemia hospital admissions increased with deprivation for all age groups. Rates of admissions increased with unemployment and level of benefit reductions and decreased with GDHI.

Poisson fixed effects analysis of the association between total LA service spending and principal and total nutritional anaemia hospital admissions

A £100 per capita higher LA service spending was associated with 1.9% lower total nutritional anaemia hospital admissions (adjusted IRR: 0.981, 95% CI: 0.964, 0.999) (Table 3). This relationship was only statistically significant in adults aged ≥15 years. A £100 higher LA service spend per capita was associated with 2.2% lower total nutritional anaemia hospital admissions in the adjusted model for people aged 15-64, and 2.1% lower rates in 65+ respectively (IRR for 15-64: 0.978, 95% CI: 0.959, 0.998; IRR for 65+: 0.979, 95% CI: 0.960, 0.997). When stratified by IMD, this relationship was statistically significant in the 1st (most deprived) (adjusted IRR for all age groups: 0.974, 95% CI: 0.951, 0.998) and 4th (adjusted IRR for all age groups: 0.883, 95% CI: 0.831, 0.939) quintiles. This persisted in adults. When stratified by level of reductions to working age benefits, this relationship was present in the 2nd quartile (adjusted IRR for all age groups: 0.963, 95% CI: 0.935, 0.991) and 4th quartile for 65+ (adjusted IRR for 65+: 0.973, 95% CI: 0.947, 0.999).

When we investigated this relationship stratified by sex, we found that this relationship was statistically significant for males only (Table 4)(Appendix Table 3). A £100 per capita higher LA service spending was associated with 2.5% lower total nutritional anaemia admissions in males (adjusted IRR 0.975, 95% CI: 0.957, 0.993). Similarly to above, the relationship was statically significant in the 1st (adjusted IRR: 0.968, 95% CI: 0.944, 0.993) and 4th (adjusted IRR: 0.872, 95% CI: 0.817, 0.932) IMD quintiles for males, but none of the results were statistically significant for females when stratified by IMD. When stratified by reductions to working age benefits, higher LA spending was statistically significantly associated with lower hospital admissions for nutritional anaemias in the 2nd (adjusted IRR: 0.955, 95% CI: 0.926, 0.985) and 4th (adjusted IRR: 0.970, 95% CI: 0.942, 1.000) quartiles for males only.

When we investigated this relationship with principal rather than total nutritional anaemia hospital admissions as the outcome, this relationship was no longer statistically significant (adjusted IRR: 0.987, 95% CI: 0.963, 1.011) (Appendix 4). LA spending was not statistically significantly associated with principal nutritional anaemia hospital admissions in any of the age groups: 0-14 (adjusted IRR: 0.996, 95% CI: 0.945, 1.051), 15-64 (adjusted IRR: 0.986, 95% CI: 0.957, 1.015), and 65+ (adjusted IRR: 0.983, 95% CI: 0.957, 1.009). When stratified by IMD, a statistically significant relationship was present for the 4th quintile only (eg. IRR for all ages: 0.838, 95% CI: 0.759, 0.925).

Sensitivity analysis: excluding diagnoses which may cause a nutritional deficiency

The increase in nutritional anaemias over time (Fig. 2) was much less steep when nutritional anaemia admissions alongside a diagnosis of a condition which may cause a nutritional deficiency were excluded (Appendix 5). When nutritional anaemia hospital admissions that were alongside a principal or secondary diagnosis of another condition which may cause a nutritional deficiency were

For peer review only

excluded in the Poisson regression analysis, generally the relationship between LA spending and total nutritional anaemia hospital admissions was not statistically significant (for example, adjusted IRR for all age groups: 0.991, 95% CI: 0.974, 1.009) (Appendix 6). There were some significant relationships in the middle IMD and level of reductions to working age benefits (eg. adjusted IRR for quintile 2 of reductions to working age benefits for 15-64: 0.960, 95% CI: 0.924, 0.997).

Sensitivity analysis: total LA service expenditure excluding social care

When social care was excluded from the exposure variable, a £100 increase in LA service spending was associated with a 4.0% decrease in total nutritional anaemia hospital admissions (adjusted IRR: 0.960, 95% CI: 0.939, 0.982) (Appendix 7). This relationship remained in the 15-64 age group (adjusted IRR for 15-64: 0.958, 95% CI: 0.934, 0.983) and the 65+ age group (adjusted IRR for 65+: 0.958, 95% CI: 0.936, 0.980) when stratified by age. Statistically significantly greater reductions in nutritional anaemias were seen in the first and fourth IMD quintiles, suggesting effect modification by deprivation. Little effect modification was seen by level of reductions to working age benefits.

DISCUSSION

This analysis of data from 2005-18 found that reductions in spending due to austerity policies may have increased nutritional anaemia hospital admissions, especially in more deprived areas. LA service spending increased by 9% between 2005 and 2009 then decreased by 20% between 2010 and 2018, following the introduction of austerity policies in 2010. Total nutritional anaemia hospital admissions also increased between 2005 and 2018. We found that a £100 higher LA service spending was associated with 1.9% lower total nutritional anaemia hospital admissions. This was driven by admissions in adults. A 2.6% lower admission rate was seen with a £100 higher expenditure in the most deprived LAs, suggesting greater impacts by deprivation, especially since these LAs experienced greater service spending reductions overall (a 28% reduction was seen in the most deprived quintile compared to a 12% reduction in the least deprived quintile between 2010 and 2018). However, we also found that a £100 higher expenditure was statistically significantly associated with a reduction in admissions in quintile 4 (one of the least deprived quintiles), suggesting that there may be impacts across the spectrum of LAs in terms of deprivation. Regardless of the relative effects of spending across levels of deprivation, the far greater cuts to spending in more deprived areas mean the absolute impact on nutritional anaemias will have been felt disproportionately in these areas.

We found that total nutritional anaemia hospital admissions increased between 2005 and 2018. This aligns with previous research suggesting decreases in micronutrient consumption and corresponding

For peer review only

increases in nutritional anaemias in the UK(5, 55, 56). We identified an overall increase in total nutritional anaemia hospital admissions which plateaued between 2010-2013, and then increased again. Research has found that between 2007 and 2011 the percentage of patients known to have had nutritional screening increased from 67-78% to 86-95%(57). Therefore, the increase in nutritional anaemia hospital admissions between 2005-2010 may be an artefact of increased screening over this period, with the plateau over the following years representing nutritional screening reaching near-saturation of hospital admissions. Given that nutritional screening was approaching saturation as austerity was being rolled out, an increase in nutritional anaemias after 2010 - during austerity - may be in part explained by changes in LA spending as per our hypothesis.

We found that the impact of LA spending on nutritional anaemia admissions was statistically significant in males but not females. This may be due to biological mechanisms – males have higher dietary reference values than women for most micronutrients (with the exception of iron to cover for iron losses during menstruation for women), although there is a lack of nuance in these reference values with regards to more subtle differences in growth and maturation rates and health vulnerabilities(58). Analyses of UK National Diet and Nutrition Survey data have found that women were more vulnerable than men to micronutrient deficiencies. It is possible that some women vulnerable to micronutrient deficiencies were already likely to be admitted with nutritional anaemias prior to austerity policies, particularly due to monitoring during pregnancy, whereas these impacts may have pushed men who were vulnerable to deficiencies into being admitted with nutritional anaemias. With regards to differences in causal pathways, it has been suggested that women have been more affected by the UK government's austerity policies than men(59). Thus, we might have expected to see greater or more statistically significant effects in women. Further individual level research, taking into account causal pathways, is needed to understand these sex differences.

Our study suggests that these decreases in LA service spending may have health impacts. This supports other studies which suggest that decreases in LA service spending may adversely affect health and mortality(14, 60). Our research adds new evidence that there may be associations with nutritional anaemias in addition to other health outcomes previously examined. As nutritional anaemias are important risk factors for diseases, this may be one pathway through which reductions in LA service expenditure may be associated with health. More deprived areas also experienced greater reductions in LA service spending, meaning the absolute increase in nutritional deficiencies attributable to service spending cuts will have been greater in more deprived areas, which already have greater health needs. This aligns with other evidence that austerity measures had a greater

For peer review only

impact in more deprived areas and may have widened health inequalities due to the role of LA services in improving the social determinants of health(9, 10, 12, 13).

This research has several strengths. We are the first to examine associations between austerity policies and nutritional anaemia hospital admissions. We used a large panel dataset with coverage of all NHS hospitals for 312 LAs in England, over a long time period (2005-2018). We used a fixed effects modelling strategy and were able to account for all time-invariant factors that differ between LAs and changing population sizes, allowing us to assess within-LA change in hospital admissions over time. Furthermore, our sensitivity analysis found a stronger relationship between total LA service spending and total nutritional anaemia hospital admissions when social care spending was excluded. This suggests that social care spending may be blunting the relationship between LA service spending and total nutritional anaemia hospital admissions, likely due to higher levels of nutritional anaemias requiring higher levels of social care spending(54). This further supports our hypothesis that austerity measures may have led to increased nutritional anaemia hospital admissions.

Our study also has some limitations. As previously described, we used counts of nutritional anaemia hospital admissions, which may be an artefact of hospital processes such as more screening leading to more diagnoses. As we used data on hospital admissions, we did not examine diagnoses by GPs, which may give a more complete picture of potential impacts on nutritional anaemias at the population level. We were also unable to break admissions down by severity, or to access data from laboratory tests, which may have picked up more cases of anaemia than were coded in the HES data. Further research could usefully explore these issues, as well as potential impacts of anaemia on the need for transfusions. Furthermore, the relationship we have described was only statistically significant when total nutritional anaemia hospital admissions were included as the outcome measure and not when principal nutritional anaemia hospital admissions were the outcome. Therefore, it is possible that the relationship seen may be a result of LA service spending being associated with increases in people accessing hospitals for other health conditions and nutritional anaemias also being present, leading to more total nutritional anaemia hospital admissions which would otherwise have been in the community and undiagnosed. One possibility is that such increases in hospital access may be due to more general impacts of austerity policies on health outcomes. Additionally, our exposure data were per financial year and our outcome data were per calendar year, representing a potential mismatch in time periods assessed. Another limitation is that we did not obtain outcome data by both age and sex.

For peer review only

Sensitivity analyses excluding hospital admissions alongside a medical condition which may cause a nutritional deficiency did not identify a relationship between LA service spending and total nutritional anaemia hospital admissions. This suggests that the relationship between LA spending and nutritional anaemia hospital admissions is driven by changes in the prevalence of these other conditions. The degree to which nutritional anaemias may be caused by other medical conditions is difficult to assess in routine data without more detailed clinical parameters and we excluded a range of common conditions such as cancers and type 2 diabetes where nutritional deficiencies are common. These diseases have a nutritional component, particularly with regards to their association with obesity, and thus may represent wide-ranging impacts of austerity policies on nutrition in terms of both nutritional anaemias and chronic disease. Together our analyses suggest that the burden of nutritional anaemias is increasing over time and that this is more apparent in areas with greater levels of deprivation, greater levels of medical comorbidity, and potentially in areas with more severe reductions in LA spending(61).

Our panel study suggests that reductions in LA service spending due to UK austerity policies may be associated with an increase in nutritional anaemias. This adds further evidence to the growing evidence base regarding potential impacts of these policies on health and health inequalities. Nutritional anaemias have wide-ranging impacts on health. Thus, policy-makers should consider associations between fiscal policies and health prior to their introduction. This is particularly pertinent given the budget deficit as well as possible further reductions in LA budgets alongside tax rises coupled with a emerging cost of living crisis. In the context of the proposed levelling-up agenda in England, this study adds more evidence to support the need for reinvestment in LA services as a means to improve population health outcomes and reduce health inequalities(62). Nutritional anaemias fell between 2005 and 2009, a period of a national health inequalities strategy in England which entailed sustained investment in LAs, the NHS, prevention and the welfare system (including tax credits, supplemental funds for pregnant women, and increases in the national pension)(63). Policy-makers should draw on this past success and consider including health outcomes in the allocation criteria for levelling-up investments(64). Indeed, a national health inequalities strategy is once more needed to level up health across the country. However, individual-level research is needed to elucidate pathways between changes to LA service spending and nutritional anaemias, particularly with regards to different areas of LA service spending.

Conclusion

Overall our analyses conclude that increased LA spending was associated with reduced hospital admissions for nutritional anaemia, with austerity having the opposite effect. This research adds to

For peer review only

other accumulating evidence of the negative impacts of austerity and the importance of maintaining and increasing public sector spending.

DATA AVAILABILITY STATEMENT

All data are available on the PLDR resource (pldr.org)

ETHICS STATEMENT

Patient Consent for Publication

Not required.

Ethics Approval

Not required

ACKNOWLEDGEMENTS

We are grateful to Christina Beatty and Steve Fothergill for sharing their data from The Uneven Impact of Welfare Reform project.

COMPETING INTERESTS

We have no competing interests to declare.

FUNDING

This study is funded by the National Institute for Health Research (NIHR) School for Public Health Research (SPHR), Grant Reference Number PD-SPH-2015. DTR is funded by the MRC on a Clinician Scientist Fellowship (MR/P008577/1).

Contributions

Study conception and design by RJ, EV, KM, AL. Data analysis was by RJ with input from RJ, EV, KM and AL. RJ wrote the first draft and all other authors (EV, KM, KD, DTR, CB, CM, AL) made significant contributions to the final version.

Figure legends

Figure 1: Logic model of potential pathways through which changes to LA spending may affect nutritional anaemias

Figure 2: Rates of principal and total nutritional anaemia hospital admissions by year, stratified by age.

Figure 3: Rates of principal and total nutritional anaemia hospital admissions by year, stratified by sex

For peer review only

For peer review only

Table 1: Mean LA service expenditure in 2005, 2010, and 2018 and percentage change, stratified by socio-demographic variables.

	Total per capita LA spending 2005	Total per capita LA spending 2010	Total per capita LA spending 2018	Change 2005-2009	Change 2010-2018	Change 2005-2018
**Total**	845.4 (237.4)	861.6 (202.7)	690.9 (103.4)	+9.2%	-19.8%	-18.3%
**Region**						
North East	1007.0 (153.9)	1060.7 (166.8)	776.7 (78.6)	+11.5%	-26.8%	-22.9%
North West	882.7 (192.5)	943.9 (187.0)	741.7 (82.9)	+14.4%	-21.4%	-16.0%
Yorkshire & the Humber	773.1 (106.5)	839.5 (119.4)	661.7 (77.1)	+16.1%	-21.2%	-14.4%
East Midlands	705.0 (64.3)	776.7 (111.0)	620.0 (72.0)	+14.5%	-20.1%	-12.1%
West Midlands	770.2 (98.7)	768.7 (120.3)	655.8 (73.1)	+5.5%	-14.7%	-14.9%
London	1399.2 (272.7)	1263.1 (236.8)	870.0 (128.1)	+3.6%	-31.1%	-37.8%
South West	767.9 (64.2)	805.0 (74.5)	678.9 (52.0)	+10.0%	-15.7%	-11.6%
East of England	764.0 (48.9)	801.7 (49.9)	658.3 (42.1)	+11.1	-17.9%	-13.8%
South East	754.0 (81.3)	742.9 (75.1)	651.6 (61.9)	+4.7%	-12.3%	-13.6%
**Percentage male**						
<48.7%	825.5 (169.8)	851.5 (172.3)	687.6 (86.2)	+10.0%	-19.3%	-16.7%
48.7%-49.1%	855.1 (240.0)	859.5 (192.8)	658.8 (71.0)	+8.0%	-23.4%	-23.0%
49.1%-49.5%	811.2 (201.6)	834.0 (173.9)	674.7 (85.1)	+9.6%	-19.1%	-16.8%
>49.5%	907.9 (350.1)	906.6 (262.9)	725.5 (131.7)	+8.2%	-20.0%	-20.1%
**Percentage working age**						
<62.6%	761.2 (60.2)	786.8 (73.6)	659.6 (69.7)	+8.4%	-16.2%	-13.4%
62.6%-64.5%	782.8 (141.0)	771.7 (111.8)	687.7 (84.2)	+4.0%	-10.9%	-12.2%
64.5%-66.3%	786.7 (117.9)	838.9 (153.9)	700.7 (93.1)	+13.2%	-14.5%	-10.9%
>66.3%	985.5 (347.5)	995.5 (275.0)	812.2 (151.8)	+7.6%	-18.4%	-17.6%
**GDHI per capita**[1]						
<£15,892	884.8 (198.6)	956.1 (174.3)	740.0 (94.0)	+13.7%	-22.6%	-16.4%
£15,892-£18,346	782.9 (154.5)	795.8 (94.7)	659.9 (72.3)	+11.7%	-17.1%	-15.7%
£18,346-£21,422	824.6 (245.3)	816.3 (173.0)	656.8 (67.0)	+6.0%	-19.5%	-20.4%
>£21,422	893.9 (320.5)	891.9 (297.1)	708.5 (123.8)	+7.2%	-20.6%	-20.7%
**Unemployment rate**						
<3.8%	722.9 (48.7)	785.9 (75.8)	658.2 (70.2)	+19.9%	-16.3%	-9.0%
3-8%-5.1%	802.5 (117.0)	732.2 (58.6)	690.2 (92.8)	-2.1%	-5.7%	-14.0%
5.1%-6.8%	926.7 (177.4)	761.0 (83.8)	801.8 (141.5)	-12.3%	+5.4%	-13.5%
>6.8%	1352.3 (335.9)	972.6 (227.9)	842.4 (64.0)	-22.8%	-13.4%	-37.7%
**IMD**						
1 (most deprived)	1068.2 (326.0)	1084.2 (242.4)	782.5 (121.7)	+10.6%	-27.8%	-26.8%
2	883.2 (246.4)	914.2 (201.6)	713.0 (116.2)	+10.5%	-22.0%	-19.3%
3	798.0 (136.5)	809.0 (97.1)	664.0 (54.3)	+7.6%	-17.9%	-16.8%
4	746.4 (120.1)	769.5 (102.1)	647.3 (74.5)	+10.1%	-15.5%	-13.3%
5 (least deprived)	732.1 (89.6)	733.1 (82.6)	648.0 (62.0)	+6.8%	-11.6%	-11.5%
**Level of reductions to working age benefits per capita**						
1 (<£457)	726.6 (84.4)	731.3 (82.1)	643.6 (65.9)	+7.4%	-12.0%	-11.4%
2 (£457-£575)	805.8 (222.8)	819.3 (186.4)	675.5 (96.0)	+8.6%	-17.6%	-16.2%
3 (£575-£690)	868.8 (261.0)	885.8 (197.0)	694.2 (107.6)	+9.2%	-21.6%	-20.1%
4 (>£690)	985.3 (262.5)	1013.4 (208.1)	751.7 (108.8)	+11.3%	-25.8%	-23.7%

[1] GDHI is Gross Disposable Household Income per capita per year, an area-level measure of individuals' available money for spending or saving following payment of taxes and receipt of benefits

[2] Index of Multiple Deprivation, quintiles based on relative ranking of LAs

For peer review only

Table 2: Mean rate of principal and total nutritional anaemia hospital admissions per 100,000 LA population in 2018, stratified by socio-demographic variables.

	Mean rate of nutritional anaemia hospital admissions per 100,000 LA population in 2018 Mean (SE in brackets)							
	All ages		0-14		15-64		65+	
	Principal nutritional anaemia hospital admissions	Total nutritional anaemia hospital admissions	Principal nutritional anaemia hospital admissions	Total nutritional anaemia hospital admissions	Principal nutritional anaemia hospital admissions	Total nutritional anaemia hospital admissions	Principal nutritional anaemia hospital admissions	Total nutritional anaemia hospital admissions
**Total**	236.4 (95.8)	632.4 (213.7)	11.1 (16.9)	56.8 (41.1)	154.1 (65.0)	363.6 (145.8)	717.1 (278.3)	2101.8 (765.5)
**Region**								
North East	306.7 (106.1)	938.4 (268.3)	13.5 (6.9)	67.7 (26.2)	208.6 (79.3)	553.2 (210.4)	898.1 (329.1)	2997.9 (851.1)
North West	284.5 (88.6)	836.4 (200.5)	22.4 (35.4)	83.9 (62.5)	189.6 (72.8)	492.8 (172.2)	854.7 (308.1)	2688.0 (753.8)
Yorkshire & the Humber	278.3 (73.7)	698.0 (149.8)	7.9 (9.9)	55.2 (36.5)	181.9 (55.4)	404.8 (127.4)	804.8 (214.0)	2154.9 (374.2)
East Midlands	206.7 (86.1)	531.5 (179.1)	6.7 (8.8)	35.7 (29.0)	136.7 (61.1)	299.9 (113.1)	596.0 (261.4)	1677.8 (557.2)
West Midlands	211.0 (70.6)	584.5 (155.0)	11.1 (10.7)	61.9 (42.6)	146.5 (52.8)	347.9 (113.2)	585.8 (199.5)	1788.7 (503.3)
London	183.2 (62.5)	644.2 (140.2)	13.7 (9.1)	85.4 (29.3)	137.5 (49.4)	415.1 (109.8)	719.9 (209.0)	2956.2 (763.2)
South West	271.2 (119.0)	628.3 (204.4)	6.8 (8.4)	43.2 (28.7)	164.0 (69.0)	332.6 (108.1)	730.1 (313.1)	1825.1 (592.2)
East of England	255.2 (115.1)	620.2 (217.7)	9.8 (14.3)	47.7 (32.1)	157.2 (72.1)	354.6 (127.1)	786.0 (304.0)	1985.4 (582.0)
South East	205.8 (71.6)	515.4 (150.5)	9.5 (14.2)	48.1 (34.5)	127.7 (48.1)	276.6 (90.3)	651.3 (232.8)	1758.6 (571.3)
**Percentage male**								
<48.7%	238.0 (99.8)	614.5 (216.4)	9.7 (13.4)	48.9 (32.3)	144.9 (60.9)	332.2 (127.0)	662.1 (249.0)	1834.3 (581.6)
48.7%-49.1%	250.4 (100.3)	646.5 (206.5)	8.1 (9.6)	47.3 (31.1)	153.4 (61.9)	351.9 (128.1)	725.0 (298.2)	1981.2 (625.1)
49.1%-49.5%	240.4 (104.9)	633.1 (248.4)	12.3 (16.5)	57.9 (43.3)	159.7 (76.5)	364.8 (170.2)	725.3 (305.2)	2082.4 (844.3)
>49.5%	223.8 (81.3)	635.5 (186.0)	12.9 (22.0)	66.9 (47.5)	156.0 (59.4)	390.7 (142.7)	742.7 (260.1)	2372.5 (805.6)
**Percentage working age**								
<62.6%	241.5 (103.4)	624.5 (223.7)	9.2 (14.3)	47.8 (37.5)	148.4 (65.3)	337.0 (139.1)	662.6 (275.8)	1826.9 (616.5)
62.6%-64.5%	256.9 (85.8)	681.2 (223.6)	12.0 (11.2)	60.9 (38.5)	174.3 (67.9)	411.4 (167.4)	801.5 (259.2)	2278.9 (690.9)
64.5%-66.3%	223.0 (85.1)	591.4 (175.7)	17.8 (37.8)	75.1 (59.1)	163.7 (66.6)	381.9 (137.9)	763.8 (298.2)	2230.1 (704.6)
>66.3%	191.5 (71.8)	609.0 (168.0)	12.6 (9.5)	72.8 (35.2)	136.1 (49.1)	375.1 (115.6)	757.2 (266.2)	2799.7 (907.6)
**GDHI[1]**								

For peer review only

<£15,892	301.8 (102.0)	817.8 (244.4)	19.4 (30.4)	86.8 (59.3)	208.7 (69.3)	507.4 (180.5)	889.2 (281.3)	2628.6 (746.9)
£15,892-£18,346	241.0 (103.8)	649.8 (221.2)	10.1 (9.4)	52.1 (31.5)	156.2 (67.1)	368.4 (135.5)	704.1 (288.3)	2066.1 (692.3)
£18,346-£21,422	235.7 (90.7)	598.8 (172.8)	9.3 (12.9)	45.7 (30.2)	151.3 (57.2)	331.2 (103.6)	692.8 (295.8)	1911.2 (630.6)
>£21,422	200.6 (70.5)	549.8 (155.5)	9.1 (12.6)	53.2 (36.1)	126.9 (48.3)	310.9 (109.1)	656.3 (219.9)	1995.6 (804.9)
**Unemployment rate**								
<3.8%	230.6 (96.5)	579.0 (191.7)	8.3 (13.1)	44.1 (31.3)	140.3 (61.3)	310.7 (111.1)	673.5 (278.9)	1793.7 (579.8)
3-8%-5.1%	242.8 (92.5)	656.6 (203.7)	12.1 (21.6)	63.4 (47.2)	164.3 (60.5)	387.9 (131.7)	750.3 (276.9)	2226.3 (670.2)
5.1%-6.8%	237.5 (102.4)	752.4 (242.7)	18.9 (16.1)	84.0 (39.0)	175.0 (76.7)	482.5 (179.4)	786.8 (256.0)	2911.8 (887.9)
>6.8%	279.4 (84.1)	856.6 (256.7)	19.7 (8.8)	117.9 (28.0)	221.2 (60.6)	602.4 (184.1)	867.9 (297.3)	2873.1 (706.2)
**IMD**[1]								
1 (most deprived)	254.9 (99.1)	764.9 (230.5)	20.2 (28.0)	90.5 (51.6)	186.5 (75.1)	493.9 (173.0)	817.3 (267.4)	2781.7 (775.2)
2	246.6 (113.1)	670.4 (234.7)	11.1 (9.3)	61.4 (36.6)	165.0 (66.7)	390.3 (138.7)	742.9 (321.4)	2252.2 (847.3)
3	241.1 (89.3)	622.9 (177.6)	11.0 (13.5)	51.9 (32.2)	156.2 (57.2)	350.2 (103.7)	710.8 (275.3)	1993.3 (602.7)
4	238.8 (94.4)	595.1 (180.2)	7.9 (13.6)	41.9 (30.8)	142.8 (59.6)	314.4 (106.7)	692.2 (277.3)	1829.7 (541.6)
5 (least deprived)	202.3 (73.3)	512.3 (153.8)	5.5 (8.9)	38.5 (28.1)	120.9 (45.6)	271.2 (82.4)	625.1 (214.7)	1665.2 (467.5)
**Level of reductions to working age benefits per capita**								
1 (<£457)	206.2 (75.7)	521.9 (150.1)	5.3 (8.5)	39.4 (30.0)	123.0 (46.8)	275.7 (82.5)	630.4 (231.7)	1679.5 (484.8)
2 (£457-£575)	238.8 (100.4)	610.6 (196.9)	11.5 (16.1)	50.2 (33.5)	145.3 (60.7)	327.4 (111.3)	703.0 (284.4)	1962.2 (717.5)
3 (£575-£690)	239.5 (105.0)	639.8 (201.6)	9.3 (8.6)	51.6 (30.9)	158.2 (62.7)	366.4 (115.2)	744.6 (305.4)	2208.3 (748.8)
4 (>£690)	263.3 (92.7)	759.8 (233.6)	18.9 (26.3)	88.0 (51.4)	192.4 (70.0)	489.5 (172.6)	795.5 (263.7)	2571.1 (801.0)

[1] GDHI is Gross Disposable Household Income per capita per year, an area-level measure of individuals' available money for spending or saving following payment of taxes and receipt of benefits

[2] Index of Multiple Deprivation, quintiles based on relative ranking of LAs

For peer review only

Table 3: Association between LA service spending and total nutritional anaemia hospital admissions, shown as the Incident Rate Ratio and stratified by age (95% Confidence Intervals in brackets)

	Incidence Rate Ratio for total nutritional anaemia hospital admissions by LA with an £100 increase in total LA service spending			
	All ages	0-14	15-64	65+
Full Sample[1]	0.981 (0.964, 0.999) p=0.038	0.974 (0.904, 1.050) p=0.488	0.978 (0.959, 0.998) p=0.033	0.979 (0.960, 0.997) p=0.024
IMD[2]				
1 (most deprived)	0.974 (0.951, 0.998) p=0.032	0.943 (0.834, 1.066) p=0.348	0.971 (0.943, 0.999) p=0.043	0.974 (0.950, 0.999) p=0.038
2	0.991 (0.949, 1.035) p=0.678	1.030 (0.980, 1.082) p=0.251	0.999 (0.957, 1.043) p=0.960	0.982 (0.936, 1.030) p=0.452
3	1.016 (0.976, 1.058) p=0.431	1.032 (0.938, 1.135) p=0.524	1.019 (0.968, 1.073) p=0.472	1.006 (0.966, 1.047) p=0.779
4	0.883 (0.831, 0.939) p<0.001	0.929 (0.790, 1.092) p=0.371	0.851 (0.792, 0.915) p<0.001	0.891 (0.838, 0.946) p<0.001
5 (least deprived)	1.011 (0.955, 1.070) p=0.712	1.014 (0.860, 1.195) p=0.870	0.989 (0.922, 1.063) p=0.768	1.018 (0.966, 1.072) p=0.505
Level of reductions to working age benefits per capita				
1 (lowest reductions)	1.011 (0.961, 1.063) p=0.680	1.013 (0.877, 1.170) p=0.864	1.000 (0.946, 1.056) p=0.985	1.013 (0.962, 1.067) p=0.616
2	0.963 (0.935, 0.991) p=0.010	0.955 (0.889, 1.026) p=0.208	0.954 (0.920, 0.989) p=0.010	0.963 (0.935, 0.992) p=0.012
3	0.992 (0.952, 1.032) p=0.671	1.029 (0.928, 1.141) p=0.587	0.995 (0.955, 1.036) p=0.803	0.985 (0.942, 1.030) p=0.500
4 (greatest reductions)	0.975 (0.949, 1.003) p=0.077	0.935 (0.824, 1.061) p=0.294	0.973 (0.941, 1.006) p=0.104	0.973 (0.947, 0.999) p=0.043

[1] Adjusted by percentage working age, percentage male, GDHI and unemployment rate.
[2] Index of Multiple Deprivation, quintiles based on relative ranking of LAs.

For peer review only

Table 4: Impact of LA service spending on total nutritional anaemia hospital admissions, shown as the Incident Rate Ratio and stratified by sex (95% Confidence Intervals in brackets)

	Incidence Rate Ratio for total nutritional anaemia hospital admissions by LA with an £100 increase in total LA service spending		
	All Ages	Male	Female
Full Sample[1]	0.981 (0.964, 0.999) p=0.038	0.975 (0.957, 0.993) p=0.007	0.992 (0.974, 1.010) p=0.358
IMD[2]			
1 (most deprived)	0.974 (0.951, 0.998) p=0.032	0.968 (0.944, 0.993) p=0.012	0.980 (0.956, 1.005) p=0.118
2	0.991 (0.949, 1.035) p=0.678	0.988 (0.947, 1.030) p=0.563	1.005 (0.962, 1.050) p=0.818
3	1.016 (0.976, 1.058) p=0.431	1.009 (0.962, 1.058) p=0.707	1.032 (0.989, 1.076) p=0.151
4	0.883 (0.831, 0.939) p<0.001	0.872 (0.817, 0.932) p<0.001	0.902 (0.851, 0.956) p<0.001
5 (least deprived)	1.011 (0.955, 1.070) p=0.712	1.001 (0.936, 1.071) p=0.967	1.029 (0.967, 1.094) p=0.367
Level of reductions to working age benefits per capita			
1 (lowest reductions)	1.011 (0.961, 1.063) p=0.680	1.013 (0.962, 1.067) p=0.623	1.020 (0.962, 1.083) p=0.506
2	0.963 (0.935, 0.991) p=0.010	0.955 (0.926, 0.985) p=0.004	0.981 (0.951, 1.011) p=0.210
3	0.992 (0.952, 1.032) p=0.671	0.983 (0.944, 1.024) p=0.419	0.999 (0.960, 1.040) p=0.960
4 (greatest reductions)	0.975 (0.949, 1.003) p=0.077	0.970 (0.942, 1.000) p=0.048	0.986 (0.957, 1.015) p=0.330

[1] Adjusted by percentage working age, percentage male, GDHI and unemployment rate.
[2] Index of Multiple Deprivation, quintiles based on relative ranking of LAs

For peer review only

REFERENCES

1. World Health Organisation. Anaemia 2021 [Available from: https://www.who.int/health-topics/anaemia#tab=tab_2.
2. von Haehling S, Jankowska EA, van Veldhuisen DJ, Ponikowski P, Anker SD. Iron deficiency and cardiovascular disease. Nature Reviews Cardiology. 2015;12(11):659-69.
3. Darnton-Hill I, Webb P, Harvey PWJ, Hunt JM, Dalmiya N, Chopra M, et al. Micronutrient deficiencies and gender: social and economic costs. The American Journal of Clinical Nutrition. 2005;81(5):1198S-205S.
4. Lim SS, Vos T, Flaxman AD, Danaei G, Shibuya K, Adair-Rohani H, et al. A comparative risk assessment of burden of disease and injury attributable to 67 risk factors and risk factor clusters in 21 regions, 1990-2010: a systematic analysis for the Global Burden of Disease Study 2010. Lancet. 2012;380(9859):2224-60.
5. NHS Digital. Hospital Admitted Patient Care Activity 2020 [Available from: https://digital.nhs.uk/data-and-information/publications/statistical/hospital-admitted-patient-care-activity.
6. House of Commons Environmental Audit Committee. Sustainable Development Goals in the UK follow up: hunger, malnutrition and food insecurity in the UK. London: House of Commons; 2019.
7. HM Treasury. Budget 2010. 2010.
8. HM Treasury. Chancellor announces reforms to the welfare system 2010 [Available from: https://www.gov.uk/government/news/chancellor-announces-reforms-to-the-welfare-system.
9. Smith NA, Phillips D, Simpson P, Eiser D, Trickey M. A time of revolution? British local government finance in the 2010s. Institute for Fiscal Studies; 2016.
10. Centre for Cities. Cities Outlook 2019: A Decade of Austerity 2019.
11. Ministry of Housing Communities & Local Government. General Fund Revenue Account Outturn 2019-20 Specific guidance notes for completing the REVENUE OUTTURN (RO) suite of forms. 2020.
12. Bambra C, Gibson M, Sowden A, Wright K, Whitehead M, Petticrew M. Tackling the wider social determinants of health and health inequalities: evidence from systematic reviews. Journal of Epidemiology and Community Health. 2010;64(4):284.
13. Marmot M, Allen, J, Boyce, T, et al. Health Equity in England: The Marmot Review 10 Years On. 2020.
14. Alexiou A, Fahy K, Mason K, Bennett D, Brown H, Bambra C, et al. Local government funding and life expectancy in England: a longitudinal ecological study. The Lancet Public Health. 2021;6:e641-47.
15. Jenkins RH, Aliabadi S, Vamos EP, et al. The relationship between austerity and food insecurity in the UK: A systematic review. EClinicalMedicine. 2021;33:100781.
16. Humphries RH, P; Charles, A; Thorlby, R; Holder, H. Social care for older people: Home truths. The Kings Fund 2016. Report No.: 978 1 909029 66 8.
17. NHS Digital. Community Care Statistics, Social Services Activity, England, 2013-2014 2014 [Available from: https://digital.nhs.uk/data-and-information/publications/statistical/adult-social-care-activity-and-finance-report/community-care-statistics-social-services-activity-england-2013-14-final-release.
18. Penny J, Slay J. Surviving Austerity: Local Voices and Local Action in England's Poorest Neighbourhoods: New Economics Foundation; 2013 [Available from: https://www.barrowcadbury.org.uk/wp-content/uploads/2013/08/Surviving-Austerity.pdf.
19. Wright L, Vance L, Sudduth C, Epps JB. The Impact of a Home-Delivered Meal Program on Nutritional Risk, Dietary Intake, Food Security, Loneliness, and Social Well-Being. Journal of Nutrition in Gerontology and Geriatrics. 2015;34(2):218-27.
20. Dewar M, Dickinson A, Smeeton N. Tracking and treating malnutrition: a retrospective observational study of the nutritional status of vulnerable people accessing a meals-on-wheels (MOW) service. Prim Health Care Res Dev. 2020;21:e19-e.
21. National Audit Office. Financial Sustainability of Local Authorities 2018.

For peer review only

22. Pollack CE, Griffin BA, Lynch J. Housing Affordability and Health Among Homeowners and Renters. American Journal of Preventive Medicine. 2010;39(6):515-21.
23. Office for National Statistics. Family spending in the UK: April 2018 to March 2019 2020 [Available from: https://www.ons.gov.uk/peoplepopulationandcommunity/personalandhouseholdfinances/expenditure/bulletins/familyspendingintheuk/april2018tomarch2019.
24. Jones NRV, Conklin AI, Suhrcke M, Monsivais P. The Growing Price Gap between More and Less Healthy Foods: Analysis of a Novel Longitudinal UK Dataset. PLOS ONE. 2014;9(10):e109343.
25. Sheffield Political Economy Research Institute. Heating or eating and the impact of austerity. 2018.
26. Hastings A, Bailey N, Bramley G, Gannon M. Austerity urbanism in England: The 'regressive redistribution' of local government services and the impact on the poor and marginalised. Environment and Planning A: Economy and Space. 2017;49(9):2007-24.
27. Gundlach L, Resch B, Omri M. Food Deserts: Evaluating Grocery Stores and Bus Accessibility using GIS in Madison, Wisconsin. 2013.
28. Rose D, Richards R. Food store access and household fruit and vegetable use among participants in the US Food Stamp Program. Public health nutrition. 2004;7(8):1081-8.
29. Lopresti AL. The Effects of Psychological and Environmental Stress on Micronutrient Concentrations in the Body: A Review of the Evidence. Adv Nutr. 2020;11(1):103-12.
30. Barr B, Alexiou A. Local Authority Finance: Net Current Expenditure - Highways and Transport services (FIN_07_04), Verson 1.01, Place-Based Longitudinal Data Resource [Internet]; 2021. Podcast
31. Barr B, Alexiou A. Local Authority Finance: Net Current Expenditure - Social Care services (FIN_07_05), Verson 1.01, Place-Based Longitudinal Data Resource [Internet]; 2021. Podcast
32. Barr B, Alexiou A. Local Authority Finance: Net Current Expenditure - Housing services (GFRA only) (FIN_07_09), Verson 1.01, Place-Based Longitudinal Data Resource [Internet]; 2021. Podcast
33. Barr B, Alexiou A. Local Authority Finance: Net Current Service Expenditure - Cultural and related services (FIN_07_10), Verson 1.01, Place-Based Longitudinal Data Resource [Internet]; 2021. Podcast
34. Barr B, Alexiou A. Local Authority Finance: Net Current Expenditure - Environmental and regulatory services (FIN_07_11), Verson 1.01, Place-Based Longitudinal Data Resource [Internet]; 2021. Podcast
35. Barr B, Alexiou A. Local Authority Finance: Net Current Expenditure - Planning and development services (FIN_07_12), Verson 1.01, Place-Based Longitudinal Data Resource [Internet]; 2021. Podcast
36. Barr B, Alexiou A. Local Authority Finance: Net Current Expenditure - Central services (FIN_07_13), Verson 1.01, Place-Based Longitudinal Data Resource [Internet]; 2021. Podcast
37. Innes D, Tetlow G. Delivering Fiscal Squeeze by Cutting Local Government Spending. Fiscal Studies. 2015;36(3):303-25.
38. Office for National Statistics. CPIH ANNUAL RATE 00: ALL ITEMS 2015=100 2021 [Available from: https://www.ons.gov.uk/economy/inflationandpriceindices/timeseries/l55o/mm23.
39. NHS Digital. Hospital Episode Statistics (HES) 2021 [Available from: https://digital.nhs.uk/data-and-information/data-tools-and-services/data-services/hospital-episode-statistics.
[Dataset] 40. Barr B, Alexiou A. Total primary diagnoses of nutritional anaemias (D50-53) excluding primary or secondary diagnosis of D50.0, version 3 [Safeguarded Dataset], Place-Based Longitudinal Data Resource https://pldr.org
41. Barr B, Alexiou A. Total secondary diagnoses of nutritional anaemias (D50-53) excluding primary or secondary diagnosis of D50.0, version 3 [Safeguarded Dataset], Place-Based Longitudinal Data Resource [Internet]; 2021. Podcast
42. Barr B, Alexiou A. Total primary diagnoses of nutritional anaemias (D50-53) excluding primary or secondary diagnosis of D50.0, C00-C96, F50, R13, K91.2, R64, E70-90, K50.90, K51.90,

K63.2,Z99.2, N17-19,K70-77,E08-E11 and O24.4, version 3 [Safeguarded Dataset], Place-Based Longitudinal Data Resource [Internet]; 2021. Podcast
43. Barr B, Alexiou A. Total secondary diagnoses of nutritional anaemias (D50-53) excluding primary or secondary diagnosis of D50.0,C00-C96, F50, R13, K91.2, R64, E70-90, K50.90, K51.90, K63.2, Z99.2, N17-19, K70-77, E08-E11 and O24.4, version 3 [Safeguarded Dataset], Place-Based Longitudinal Data Resource [Internet]; 2021. Podcast
44. U.S. Federal Government's Department of Health and Human Services. ICD-10-CM Official Guidelines for Coding and Reporting. 2020.
45. Office for National Statistics. Lower layer Super Output Area population estimates 2020 [Available from: https://www.ons.gov.uk/peoplepopulationandcommunity/populationandmigration/populationestimates/datasets/lowersuperoutputareamidyearpopulationestimates.
46. Office for National Statistics. Regional gross disposable household income QMI 2018 [Available from: https://www.ons.gov.uk/economy/regionalaccounts/grossdisposablehouseholdincome/methodologies/regionalgrossdisposablehouseholdincomeqmi.
47. Office for National Statistics. Model-Based Estimates of ILO Unemployment for LAD/UAs in Great Britain: Guide for Users. 2006.
48. Ministry of Housing Communities & Local Government. English indices of deprivation 2015 2015 [Available from: https://www.gov.uk/government/statistics/english-indices-of-deprivation-2015].
49. Beatty C, Fothergill, S. The uneven impact of welfare reform: the financial losses to places and people. Sheffield Hallam University Centre for Regional Economic and Social Research; 2016.
50. Cherrie M, Curtis S, Baranyi G, Cunningham N, Dibben C, Bambra C, et al. A data linkage study of the effects of the Great Recession and austerity on antidepressant prescription usage. European Journal of Public Health. 2021;31(2):297-303.
51. Wooldridge JM. Econometric analysis of cross section and panel data: MIT press; 2010.
52. Wooldridge JM. Distribution-free estimation of some nonlinear panel data models. Journal of Econometrics. 1999;90(1):77-97.
53. Bertrand M, Duflo E, Mullainathan S. How Much Should We Trust Differences-In-Differences Estimates? The Quarterly Journal of Economics. 2004;119(1):249-75.
54. Elia M. The cost of malnutrition in England and potential cost savings from nutritional interventions (short version): A report on the cost of disease-related malnutrition in England and a budget impact analysis of implementing the NICE clinical guidelines/quality standard on nutritional support in adults. BAPEN; 2015.
55. Public Health England. National Diet and Nutrition Survey. Years 1 to 9 of the Rolling Programme (2008/2009 – 2016/2017): Time trend and income analyses. 2019.
56. Public Health England. National Diet and Nutrition Survey. Rolling programme Years 9 to 11 (2016/2017 to 2018/2019). 2020.
57. Russell CA, Elia M. NUTRITION SCREENING SURVEYS IN HOSPITALS IN THE UK, 2007-2011. BAPEN 2014.
58. Prentice A. Sex differences in requirements for micronutrients across the lifecourse. Proceedings of the Nutrition Society. 2021;80(3):356-64.
59. Alston P. Statement on Visit to the United Kingdom, by Professor Philip Alston, United Nations Special Rapporteur on extreme poverty and human rights 2018 [Available from: https://www.ohchr.org/EN/NewsEvents/Pages/DisplayNews.aspx?NewsID=23881&LangID=E.
60. Watkins J, Wulaningsih W, Da Zhou C, Marshall DC, Sylianteng GDC, Dela Rosa PG, et al. Effects of health and social care spending constraints on mortality in England: a time trend analysis. BMJ Open. 2017;7(11):e017722.
61. McLean G, Gunn J, Wyke S, Guthrie B, Watt GC, Blane DN, et al. The influence of socioeconomic deprivation on multimorbidity at different ages: a cross-sectional study. British Journal of General Practice. 2014;64(624):e440-e7.
62. The Rt Hon Boris Johnson MP. The Prime Minister's Levelling Up speech: 15 July 2021 2021 [Available from: https://www.gov.uk/government/speeches/the-prime-ministers-levelling-up-speech-15-july-2021.

For peer review only

63. Robinson T, Brown H, Norman PD, Fraser LK, Barr B, Bambra C. The impact of New Labour's English health inequalities strategy on geographical inequalities in infant mortality: a time-trend analysis. Journal of Epidemiology and Community Health. 2019;73(6):564-8.
64. Dixon J, Everest G. Briefing: The government's levelling up agenda The Health Foundation; 2021 [Available from: https://reader.health.org.uk/governments-levelling-up-agenda.

For peer review only

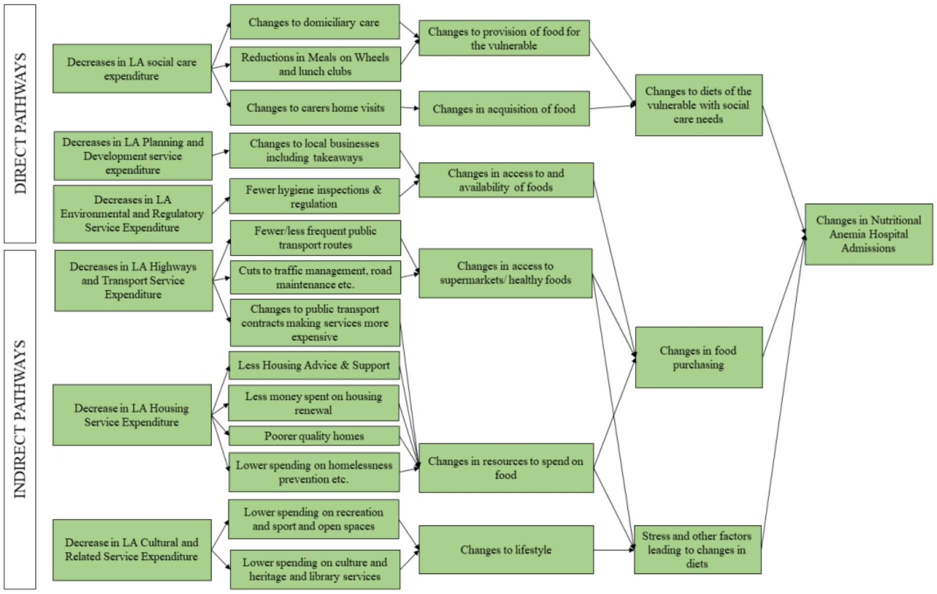

Figure 1

162x137mm (236 x 236 DPI)

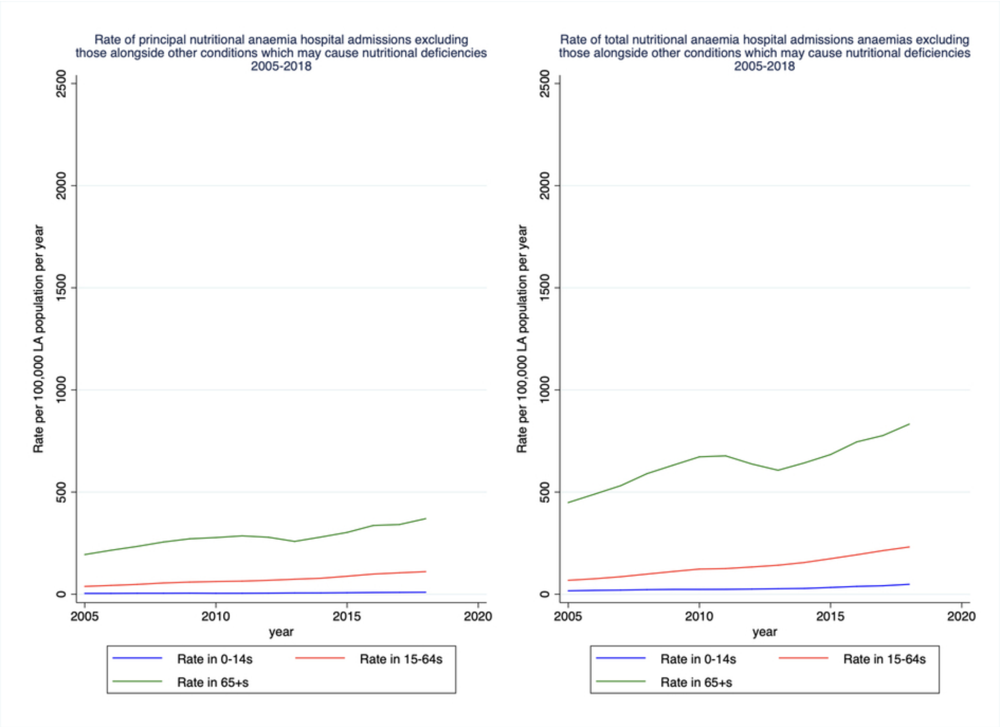

Figure 2

80x58mm (300 x 300 DPI)

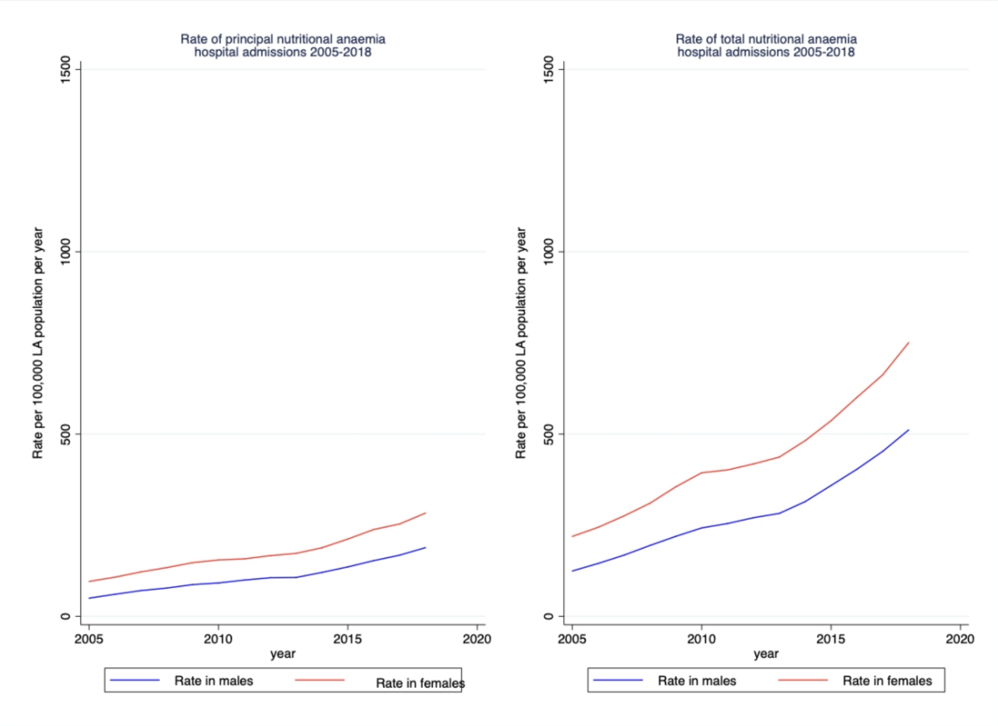

Figure 3

133x97mm (236 x 236 DPI)

Appendix 1: ICD-10 codes for conditions included in this study

ICD-10 code	Diagnosis
Principal and total nutritional anaemia hospital admissions:	
D50	Iron deficiency anaemia
D51	Vitamin B12 deficiency anaemia
D52	Folate deficiency anaemia
D53	Other nutritional anaemias
Excluding admissions alongside a principal or secondary diagnosis of:	
D50.0	Iron deficiency anaemia secondary to blood loss
C00-C96	Malignancies
F50	Eating disorders
R13	Difficulty swallowing
K91.2	Postoperative malabsorption
R64	Cachexia
E70-90	Metabolic disorders
K50.90, K51.90	GI disease including Crohn's disease and ulcerative colitis
K63.2	Fistula
Z99.2, N17-19	Kidney disease and dialysis
K70-77	Liver disease
E08-E11, O24.4	Diabetes mellitus (due to underlying condition, drug or chemical induced, type I, type II, gestational)

For peer review only

Appendix 2: Changes in mean total LA spending over time

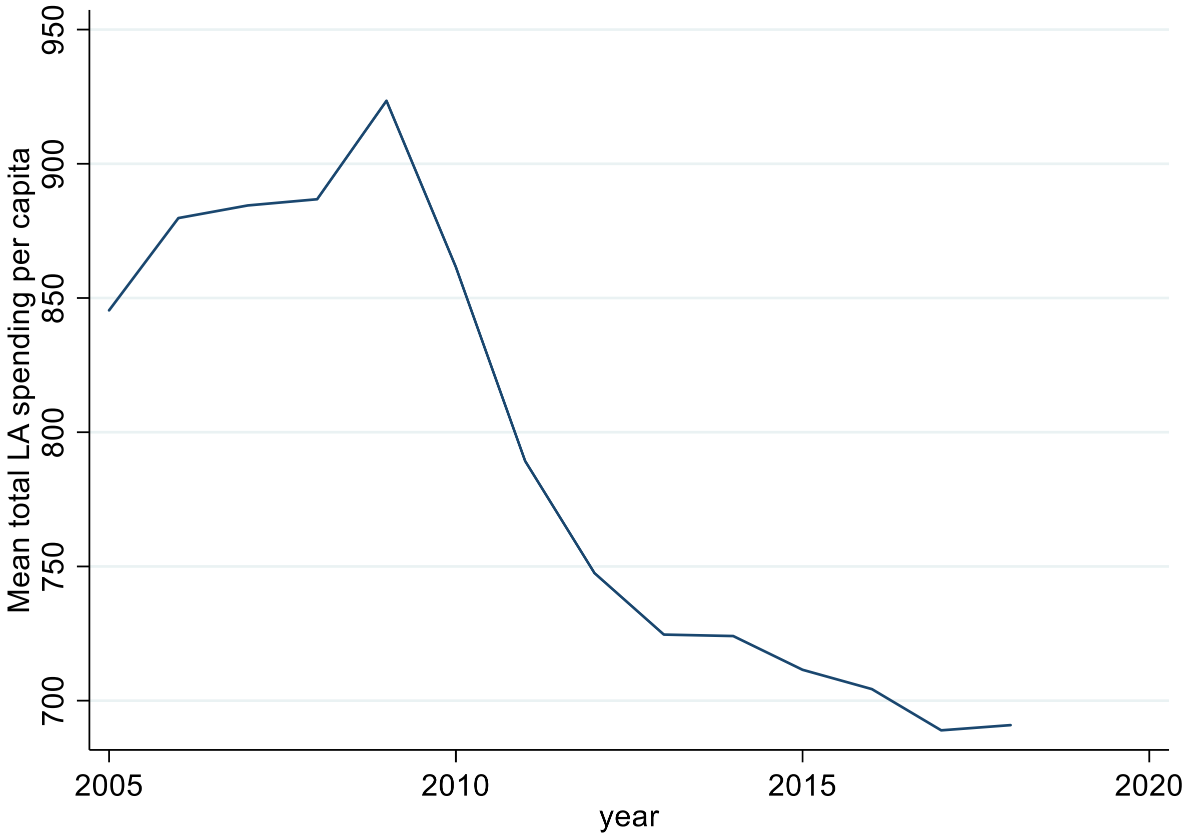

For peer review only

Appendix 3: Impact of LA service spending on total nutritional anaemia hospital admissions, shown as the Incident Rate Ratio and stratified by gender (95% Confidence Intervals in brackets)

	Incidence Rate Ratio for total nutritional anaemia hospital admissions by LA with an £100 increase in total LA service spending		
	All Ages	Male	Female
Full Sample[1]	0.981 (0.964, 0.999) p=0.038	0.975 (0.957, 0.993) p=0.007	0.992 (0.974, 1.010) p=0.358
IMD[2]			
1 (most deprived)	0.974 (0.951, 0.998) p=0.032	0.968 (0.944, 0.993) p=0.012	0.980 (0.956, 1.005) p=0.118
2	0.991 (0.949, 1.035) p=0.678	0.988 (0.947, 1.030) p=0.563	1.005 (0.962, 1.050) p=0.818
3	1.016 (0.976, 1.058) p=0.431	1.009 (0.962, 1.058) p=0.707	1.032 (0.989, 1.076) p=0.151
4	0.883 (0.831, 0.939) p<0.001	0.872 (0.817, 0.932) p<0.001	0.902 (0.851, 0.956) p<0.001
5 (least deprived)	1.011 (0.955, 1.070) p=0.712	1.001 (0.936, 1.071) p=0.967	1.029 (0.967, 1.094) p=0.367
Level of reductions to working age benefits per capita			
1 (lowest reductions)	1.011 (0.961, 1.063) p=0.680	1.013 (0.962, 1.067) p=0.623	1.020 (0.962, 1.083) p=0.506
2	0.963 (0.935, 0.991) p=0.010	0.955 (0.926, 0.985) p=0.004	0.981 (0.951, 1.011) p=0.210
3	0.992 (0.952, 1.032) p=0.671	0.983 (0.944, 1.024) p=0.419	0.999 (0.960, 1.040) p=0.960
4 (greatest reductions)	0.975 (0.949, 1.003) p=0.077	0.970 (0.942, 1.000) p=0.048	0.986 (0.957, 1.015) p=0.330

[1] Adjusted by percentage working age, percentage male, GDHI and unemployment rate.
[2] Index of Multiple Deprivation, quintiles based on relative ranking of LAs

For peer review only

Appendix 4: Association between LA service spending and principal nutritional anaemia hospital admissions, shown as the Incident Rate Ratio and stratified by age (95% Confidence Intervals in brackets).

	Incidence Rate Ratio for principal nutritional anaemia hospital admissions by LA with an £100 increase in total LA service spending			
	All Ages	0-14	15-64	65+
Full sample[1]	0.987 (0.963, 1.011) p=0.293	0.996 (0.945, 1.051) p=0.896	0.986 (0.957, 1.015) p=0.332	0.983 (0.957, 1.009) p=0.190
IMD[2]				
1 (most deprived)	0.985 (0.950, 1.022) p=0.435	0.969 (0.899, 1.043) p=0.400	0.989 (0.945, 1.035) p=0.634	0.978 (0.941, 1.016) p=0.247
2	0.988 (0.942, 1.036) p=0.611	0.984 (0.866, 1.119) p=0.806	1.000 (0.950, 1.053) p=0.999	0.975 (0.928, 1.024) p=0.311
3	1.031 (0.973, 1.092) p=0.308	1.086 (0.895, 1.319) p=0.402	1.051 (0.989, 1.116) p=0.107	1.002 (0.929, 1.082) p=0.951
4	0.838 (0.759, 0.925) p<0.001	1.255 (1.009, 1.560) p=0.041	0.798 (0.715, 0.892) p<0.001	0.853 (0.769, 0.946) p=0.003
5 (least deprived)	1.040 (0.957, 1.130) p=0.357	1.135 (0.943, 1.365) p=0.180	1.013 (0.929, 1.104) p=0.778	1.053 (0.968, 1.146) p=0.226
Level of reductions to working age benefits per capita				
1 (lowest reductions)	1.041 (0.972, 1.115) p=0.252	1.205 (1.001, 1.451) p=0.049	1.019 (0.949, 1.095) p=0.601	1.052 (0.977, 1.132) p=0.180
2	0.967 (0.927, 1.009) p=0.125	1.002 (0.835, 1.203) p=0.983	0.959 (0.910, 1.010) p=0.113	0.968 (0.927, 1.012) p=0.148
3	1.000 (0.949, 1.054) p=0.995	1.030 (0.919, 1.153) p=0.616	1.016 (0.965, 1.070) p=0.548	0.978 (0.920, 1.040) p=0.479
4 (greatest reductions)	0.980 (0.947, 1.014) p=0.242	0.975 (0.916, 1.038) p=0.430	0.979 (0.937, 1.022) p=0.333	0.975 (0.943, 1.008) p=0.140

[1] Adjusted by percentage working age, percentage male, GDHI and unemployment rate.

[2] Index of Multiple Deprivation, quintiles based on relative ranking of LAs

For peer review only

Appendix 5: Rates of principal and total nutritional anaemia hospital admissions over time, excluding those alongside other conditions which may cause nutritional deficiencies, stratified by age (2005-2018)

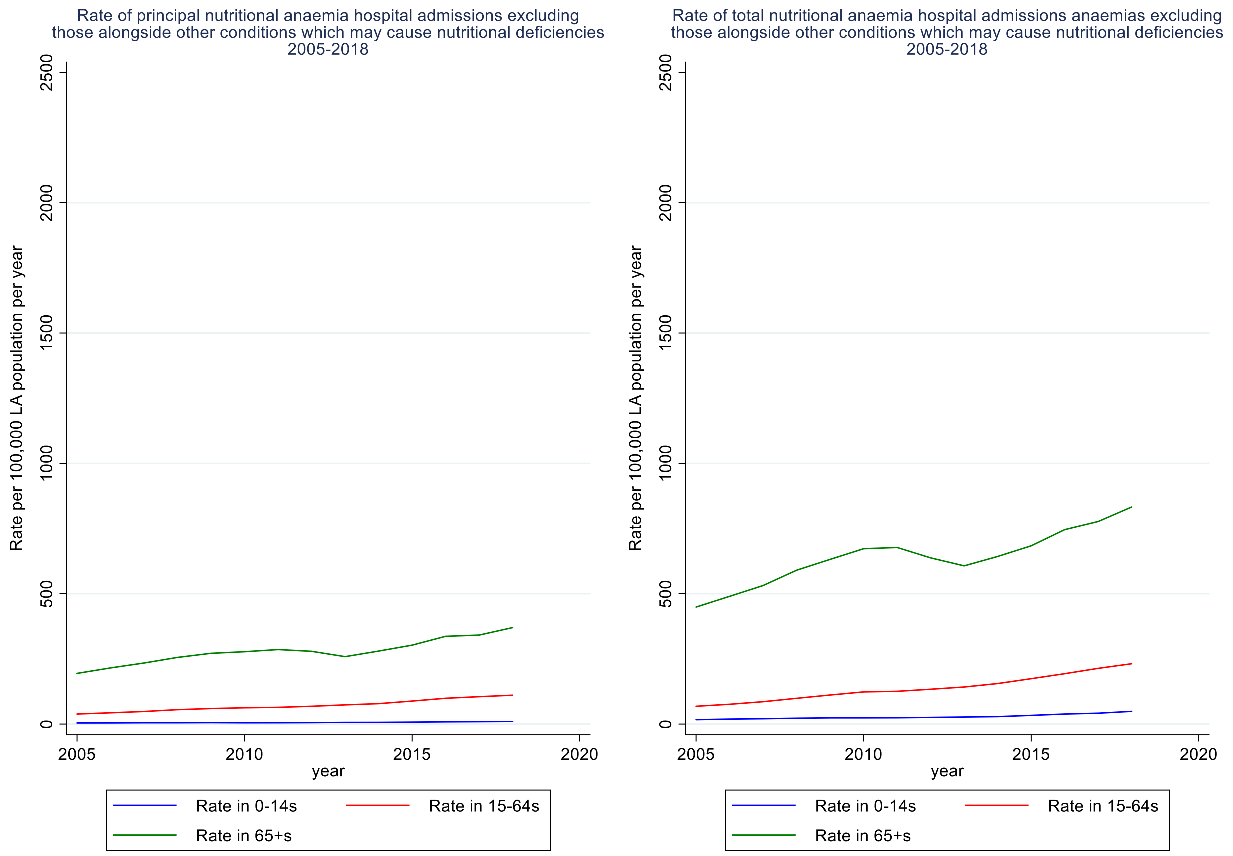

Appendix 6: Association between LA service spending and total nutritional anaemia hospital admissions excluding those alongside a principal or secondary diagnosis of another condition which may cause a nutritional deficiency, shown as the Incident Rate Ratio and stratified by age (95% Confidence Intervals in brackets)

	Incidence Rate Ratio for total nutritional anaemia hospital admissions by LA with an £100 increase in total LA service spending			
	All Ages	0-14	15-64	65+
Full sample[1]	0.991 (0.974, 1.009) p=0.312	0.967 (0.894, 1.047) p=0.408	0.989 (0.969, 1.009) p=0.279	0.998 (0.980, 1.017) p=0.829
IMD[2]				
1 (most deprived)	0.985 (0.962, 1.009) p=0.208	0.933 (0.823, 1.058) p=0.279	0.981 (0.952, 1.011) p=0.207	0.994 (0.969, 1.020) p=0.658
2	1.001 (0.961, 1.044) p=0.950	1.047 (0.989, 1.109) p=0.118	1.009 (0.967, 1.052) p=0.692	1.002 (0.955, 1.050) p=0.945
3	1.034 (0.998, 1.072) p=0.067	1.025 (0.934, 1.125) p=0.599	1.041 (0.991, 0.093) p=0.110	1.029 (0.992, 1.066) p=0.128
4	0.876 (0.818, 0.938) p<0.001	0.931 (0.789, 1.098) p=0.396	0.850 (0.786, 0.920) p<0.001	0.888 (0.828, 0.953) p=0.001
5 (least deprived)	1.030 (0.968, 1.095) p=0.357	0.977 (0.818, 1.167) p=0.799	1.024 (0.959, 1.094) p=0.472	1.038 (0.976, 1.104) p=0.237
Level of reductions to working age benefits per capita				
1 (lowest reductions)	1.026 (0.968, 1.087) p=0.390	0.979 (0.834, 1.149) p=0.792	1.031 (0.976, 1.088) p=0.279	1.027 (0.963, 1.096) p=0.412
2	0.976 (0.947, 1.005) p=0.104	0.973 (0.906, 1.045) p=0.446	0.960 (0.924, 0.997) p=0.035	0.993 (0.964, 1.023) p=0.656
3	1.004 (0.964, 1.047) p=0.839	1.030 (0.919, 1.154) p=0.616	1.012 (0.970, 1.055) p=0.590	1.002 (0.956, 1.050) p=0.946
4 (greatest reductions)	0.981 (0.956, 1.007) p=0.150	0.925 (0.813, 1.052) p=0.234	0.978 (0.946, 1.012) p=0.198	0.988 (0.963, 1.013) p=0.326

[1] Adjusted by percentage working age, percentage male, GDHI and unemployment rate.

[2] Index of Multiple Deprivation, quintiles based on relative ranking of LAs

For peer review only

Appendix 7: Association between LA service spending excluding social care spending and total nutritional anaemia hospital admissions, shown as the Incident Rate Ratio and stratified by age (95% Confidence Intervals in brackets)

	Incidence Rate Ratio for total nutritional anaemia hospital admissions by LA with an £100 increase in total LA service spending			
	All Ages	0-14	15-64	65+
Full Sample[1]	0.960 (0.939, 0.982) p<0.001	0.960 (0.869, 1.061) p=0.425	0.958 (0.934, 0.983) p=0.001	0.958 (0.936, 0.980) p<0.001
IMD[2]				
1 (most deprived)	0.957 (0.927, 0.987) p=0.006	0.903 (0.769, 1.060) p=0.213	0.950 (0.916, 0.986) p=0.007	0.962 (0.931, 0.995) p=0.025
2	0.976 (0.935, 1.020) p=0.278	1.074 (1.020, 1.131) p=0.006	0.989 (0.944, 1.037) p=0.652	0.962 (0.919, 1.008) p=0.104
3	0.988 (0.934, 1.045) p=0.670	1.087 (0.973, 1.214) p=0.141	0.990 (0.927, 1.056) p=0.750	0.973 (0.919, 1.030) p=0.349
4	0.847 (0.796, 0.901) p<0.001	0.959 (0.803, 1.146) p=0.645	0.822 (0.760, 0.888) p<0.001	0.849 (0.799, 0.901) p<0.001
5 (least deprived)	1.003 (0.931, 1.081) p=0.934	1.031 (0.889, 1.195) p=0.687	1.005 (0.924, 1.092) p=0.916	0.997 (0.933, 1.065) p=0.934
Level of reductions to working age benefits per capita				
1 (lowest reductions)	0.982 (0.914, 1.055) p=0.612	0.997 (0.845, 1.177) p=0.974	0.984 (0.910, 1.065) p=0.695	0.975 (0.912, 1.042) p=0.457
2	0.940 (0.903, 0.979) p=0.003	0.957 (0.874, 1.047) p=0.339	0.936 (0.891, 0.982) p=0.007	0.937 (0.900, 0.975) p=0.001
3	0.961 (0.922, 1.002) p=0.062	1.025 (0.912, 1.152) p=0.685	0.966 (0.924, 1.009) p=0.123	0.951 (0.909, 0.994) p=0.027
4 (greatest reductions)	0.963 (0.930, 0.997) p=0.031	0.911 (0.774, 1.072) p=0.261	0.959 (0.921, 0.999) p=0.043	0.965 (0.932, 1.000) p=0.047

[1] Adjusted by percentage working age, percentage male, GDHI and unemployment rate.

[2] Index of Multiple Deprivation, quintiles based on relative ranking of LAs

For peer review only

STROBE Statement—checklist of items that should be included in reports of observational studies

	Item No	Recommendation	Page No
**Title and abstract**	1	(*a*) Indicate the study's design with a commonly used term in the title or the abstract	1
		(*b*) Provide in the abstract an informative and balanced summary of what was done and what was found	1
**Introduction**			
Background/rationale	2	Explain the scientific background and rationale for the investigation being reported	2,3
Objectives	3	State specific objectives, including any prespecified hypotheses	3
**Methods**			
Study design	4	Present key elements of study design early in the paper	3
Setting	5	Describe the setting, locations, and relevant dates, including periods of recruitment, exposure, follow-up, and data collection	3,4
Participants	6	(*a*) *Cohort study*—Give the eligibility criteria, and the sources and methods of selection of participants. Describe methods of follow-up<br>*Case-control study*—Give the eligibility criteria, and the sources and methods of case ascertainment and control selection. Give the rationale for the choice of cases and controls<br>*Cross-sectional study*—Give the eligibility criteria, and the sources and methods of selection of participants	4
		(*b*) *Cohort study*—For matched studies, give matching criteria and number of exposed and unexposed<br>*Case-control study*—For matched studies, give matching criteria and the number of controls per case	
Variables	7	Clearly define all outcomes, exposures, predictors, potential confounders, and effect modifiers. Give diagnostic criteria, if applicable	5
Data sources/ measurement	8*	For each variable of interest, give sources of data and details of methods of assessment (measurement). Describe comparability of assessment methods if there is more than one group	5
Bias	9	Describe any efforts to address potential sources of bias	5,6
Study size	10	Explain how the study size was arrived at	6
Quantitative variables	11	Explain how quantitative variables were handled in the analyses. If applicable, describe which groupings were chosen and why	6
Statistical methods	12	(*a*) Describe all statistical methods, including those used to control for confounding	6,7
		(*b*) Describe any methods used to examine subgroups and interactions	7
		(*c*) Explain how missing data were addressed	7
		(*d*) *Cohort study*—If applicable, explain how loss to follow-up was addressed<br>*Case-control study*—If applicable, explain how matching of cases and controls was addressed<br>*Cross-sectional study*—If applicable, describe analytical methods taking account of sampling strategy	7
		(*e*) Describe any sensitivity analyses	

Continued on next page

For peer review only

**Results**			
Participants	13*	(a) Report numbers of individuals at each stage of study—eg numbers potentially eligible, examined for eligibility, confirmed eligible, included in the study, completing follow-up, and analysed	7
		(b) Give reasons for non-participation at each stage	7
		(c) Consider use of a flow diagram	7
Descriptive data	14*	(a) Give characteristics of study participants (eg demographic, clinical, social) and information on exposures and potential confounders	Table 1
		(b) Indicate number of participants with missing data for each variable of interest	Table 1
		(c) *Cohort study*—Summarise follow-up time (eg, average and total amount)	
Outcome data	15*	*Cohort study*—Report numbers of outcome events or summary measures over time	Table 1
		Case-control study—Report numbers in each exposure category, or summary measures of exposure	
		Cross-sectional study—Report numbers of outcome events or summary measures	
Main results	16	(*a*) Give unadjusted estimates and, if applicable, confounder-adjusted estimates and their precision (eg, 95% confidence interval). Make clear which confounders were adjusted for and why they were included	8
		(*b*) Report category boundaries when continuous variables were categorized	8
		(*c*) If relevant, consider translating estimates of relative risk into absolute risk for a meaningful time period	8
Other analyses	17	Report other analyses done—eg analyses of subgroups and interactions, and sensitivity analyses	8,9
**Discussion**			
Key results	18	Summarise key results with reference to study objectives	9
Limitations	19	Discuss limitations of the study, taking into account sources of potential bias or imprecision. Discuss both direction and magnitude of any potential bias	10
Interpretation	20	Give a cautious overall interpretation of results considering objectives, limitations, multiplicity of analyses, results from similar studies, and other relevant evidence	10,11
Generalisability	21	Discuss the generalisability (external validity) of the study results	11
**Other information**			
Funding	22	Give the source of funding and the role of the funders for the present study and, if applicable, for the original study on which the present article is based	13

*Give information separately for cases and controls in case-control studies and, if applicable, for exposed and unexposed groups in cohort and cross-sectional studies.

Note: An Explanation and Elaboration article discusses each checklist item and gives methodological background and published examples of transparent reporting. The STROBE checklist is best used in conjunction with this article (freely available on the Web sites of PLoS Medicine at http://www.plosmedicine.org/, Annals of Internal Medicine at http://www.annals.org/, and Epidemiology at http://www.epidem.com/). Information on the STROBE Initiative is available at www.strobe-statement.org.

For peer review only
